# Health position paper and redox perspectives - Disease burden by transportation noise

**DOI:** 10.1016/j.redox.2023.102995

**Published:** 2023-12-18

**Authors:** Mette Sørensen, Göran Pershagen, Jesse Daniel Thacher, Timo Lanki, Benedikt Wicki, Martin Röösli, Danielle Vienneau, Manuella Lech Cantuaria, Jesper Hvass Schmidt, Gunn Marit Aasvang, Sadeer Al-Kindi, Michael T. Osborne, Philip Wenzel, Juan Sastre, Ingrid Fleming, Rainer Schulz, Omar Hahad, Marin Kuntic, Jacek Zielonka, Helmut Sies, Tilman Grune, Katie Frenis, Thomas Münzel, Andreas Daiber

**Affiliations:** a Work, Environment and Cancer, Danish Cancer Institute, Copenhagen, Denmark; b Department of Natural Science and Environment, Roskilde University, Denmark; c Institute of Environmental Medicine, Karolinska Institutet, Stockholm, Sweden; d Division of Occupational and Environmental Medicine, Department of Laboratory Medicine, Lund University, Lund, Sweden; e Department of Health Security, Finnish Institute for Health and Welfare, Kuopio, Finland; f School of Medicine, University of Eastern Finland, Kuopio, Finland; g Department of Environmental and Biological Sciences, University of Eastern Finland, Kuopio, Finland; h Department of Epidemiology and Public Health, Swiss Tropical and Public Health Institute, Allschwil, Switzerland; i University of Basel, Basel, Switzerland; j Research Unit for ORL – Head & Neck Surgery and Audiology, Odense University Hospital & University of Southern Denmark, Odense, Denmark; k Department of Air Quality and Noise, Norwegian Institute of Public Health, Oslo, Norway; l Department of Medicine, University Hospitals, Harrington Heart & Vascular Institute, Case Western Reserve University, 11100 Euclid Ave, Cleveland, OH, 44106, USA; m Cardiovascular Imaging Research Center, Massachusetts General Hospital, Boston, MA, USA; n Division of Cardiology, Department of Medicine, Massachusetts General Hospital, Boston, MA, USA; o Department of Cardiology, Cardiology I, University Medical Center Mainz, Mainz, Germany; p German Center for Cardiovascular Research (DZHK), Partner Site Rhine-Main, Mainz, Germany; q Center for Thrombosis and Hemostasis, University Medical Center Mainz, Mainz, Germany; r Department of Physiology, Faculty of Pharmacy, University of Valencia, Spain; s Institute for Vascular Signalling, Centre for Molecular Medicine, Goethe University, Frankfurt Am Main, Germany; t German Center of Cardiovascular Research (DZHK), Partner Site RheinMain, Frankfurt, Germany; u Institute of Physiology, Faculty of Medicine, Justus-Liebig University, Gießen, 35392, Gießen, Germany; v Department of Biophysics, Medical College of Wisconsin, Milwaukee, WI, USA; w Institute for Biochemistry and Molecular Biology I, Faculty of Medicine, Heinrich Heine University Düsseldorf, Düsseldorf, Germany; x Leibniz Research Institute for Environmental Medicine, Düsseldorf, Germany; y Department of Molecular Toxicology, German Institute of Human Nutrition Potsdam-Rehbruecke, Nuthetal, Germany; z DZHK (German Center for Cardiovascular Research), Partner Site Berlin, Berlin, Germany; aa German Center for Diabetes Research (DZD), München-Neuherberg, Germany; ab Hematology/Oncology, Boston Children's Hospital and Harvard Medical School, Boston, MA, USA; ac Stem Cell Program, Boston Children's Hospital, Boston, MA, USA

**Keywords:** Environmental risk factors, Transportation noise, Non-communicable diseases, Stress hormones, Oxidative stress and inflammation, Adverse redox signaling

## Abstract

Transportation noise is a ubiquitous urban exposure. In 2018, the World Health Organization concluded that chronic exposure to road traffic noise is a risk factor for ischemic heart disease. In contrast, they concluded that the quality of evidence for a link to other diseases was very low to moderate. Since then, several studies on the impact of noise on various diseases have been published. Also, studies investigating the mechanistic pathways underlying noise-induced health effects are emerging. We review the current evidence regarding effects of noise on health and the related disease-mechanisms. Several high-quality cohort studies consistently found road traffic noise to be associated with a higher risk of ischemic heart disease, heart failure, diabetes, and all-cause mortality. Furthermore, recent studies have indicated that road traffic and railway noise may increase the risk of diseases not commonly investigated in an environmental noise context, including breast cancer, dementia, and tinnitus. The harmful effects of noise are related to activation of a physiological stress response and nighttime sleep disturbance. Oxidative stress and inflammation downstream of stress hormone signaling and dysregulated circadian rhythms are identified as major disease-relevant pathomechanistic drivers. We discuss the role of reactive oxygen species and present results from antioxidant interventions. Lastly, we provide an overview of oxidative stress markers and adverse redox processes reported for noise-exposed animals and humans. This position paper summarizes all available epidemiological, clinical, and preclinical evidence of transportation noise as an important environmental risk factor for public health and discusses its implications on the population level.

## Highlights

• Transportation noise is a significant environmental risk factor for many non-communicable diseases
• This position paper summarizes the clinical and epidemiological evidence of noise health effects
• Noise induces central pathomechanisms related to adverse redox signaling
• This position paper provides details on noise-mediated activation of oxidant sources and damage markers
• More human studies on noise exposure, redox biological changes, and health outcome are needed

## Graphical abstract

**Figure:**
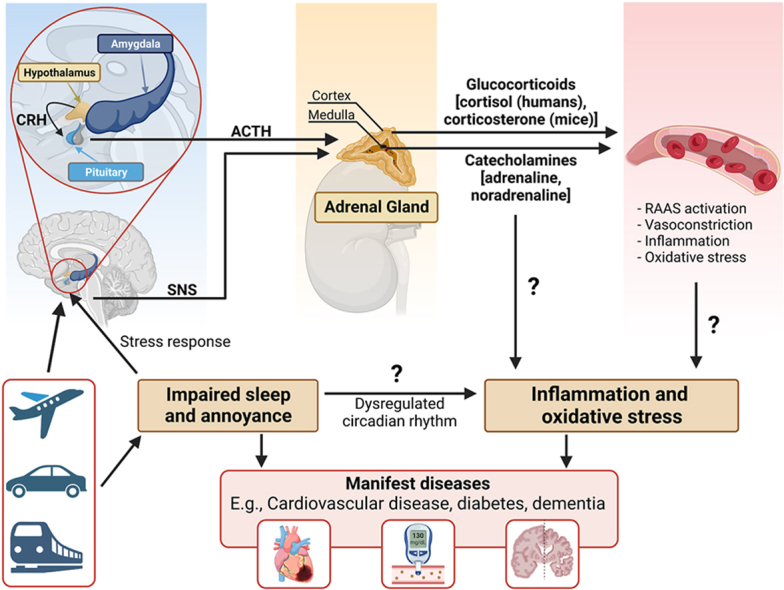

## Abbreviations

2-OH-E^+^: 2-hydroxyethidium
4-HNE: 4-hydroxynonenal
8-iso-PGF_2α_: 8-iso-prostaglandin F_2α_
8OHdG: 8-hydroxy-2’-deoxyguanosine (also 8-OH-(d)G)
ACTH: adrenocorticotropic hormone
AMPK: AMP-activated protein kinase
AT-II: angiotensin II
BH_4_: tetrahydrobiopterin
BMAL1: brain and muscle Arnt-like protein 1
BoD: burden of disease
CVD: cardiovascular disease
CI: confidence interval
CLOCK: circadian locomotor output cycles protein kaput
CRH: corticotrophin-releasing hormone
CRP: C-reactive protein
CRY: cryptochrome
ELISA: enzyme-linked immunosorbent assay
END: environmental noise directive
eNOS: endothelial nitric oxide synthase
ET-1: endothelin-1
FMD: flow-mediated dilatation
FOXO: forkhead box O protein (transcription factor)
DALYs: disability-adjusted life years
dB(A): decibel (A-weighted)
DHE: dihydroethidium
DMPO: 5,5-dimethyl-1-pyrroline-N-oxide
GBD: global burden of disease
GSH: reduced glutathione
HA: high annoyance
HO-1: heme oxygenase 1
HPA axis: hypothalamic–pituitary–adrenal axis
HPLC: high-performance liquid chromatography
HR: hazard ratio
HSD: high sleep disturbance
IHD: ischemic heart disease
IQR: interquartile range
IL: interleukin
iNOS: inducible nitric oxide synthase
K_ATP_: ATP-sensitive potassium channel
LA_eq_: equivalent A-weighted sound pressure level
LC-MS: liquid chromatography-mass spectrometry
L_den_: equivalent A-weighted sound pressure level over 24 h with a penalty of 10 dB(A) for nighttime noise (23.00–07.00) and a penalty of 5 dB(A) for evening noise (19.00–23.00)
L_den_Max: noise at the most exposed façade
L_den_Min: noise at the least exposed façade
l-NAME: N^G^-nitro-l-arginine methyl ester
LysM: lysozyme M
MACE: major adverse cardiovascular events
MAO: monoamine oxidase
MDA: malondialdehyde
MI: myocardial infarction
mPTP: mitochondrial permeability transition pore
NFκB: nuclear factor kappa B
NHL: non-Hodgkin's lymphoma
NIHL: noise-induced hearing loss
nNOS: neuronal nitric oxide synthase
NOS: nitric oxide synthase (isoforms 1 (neuronal), 2 (inducible), 3 (endothelial))
NOX: NADPH oxidase (e.g. isoforms 1, 2, 3, 4, 5)
NOX-2: NOX isoform 2 (phagocytic NADPH oxidase)
NRF-2: nuclear factor E2 related factor-2
OR: odds ratio
p47^phox^: cytosolic regulator of NOX2
p66^Shc^: SHC-transforming protein 1
PER: period
PET-CT: positron emission tomography-computed tomography
PKC: protein kinase C
PM_2.5_: fine particulate matter
RAAS: renin–angiotensin–aldosterone system
ROS: reactive oxygen species
RR: relative risk
SAPALDIA: Study on Air Pollution and Lung and Heart Diseases in Adults
SNS: sympathetic nervous system
SOD2: mitochondrial superoxide dismutase
SPL: sound pressure level
TNFα: tumor necrosis factor alpha
WHO: World Health Organization

## 1 Health impact of transportation noise

### 1.1 Introduction

Urban expansion and densification and increasing needs for transportation have led to a general rise in exposure to environmental noise from vehicles, trains, and aircraft. A recent assessment of the exposure to transportation noise in the European Union, as part of the Environmental Noise Directive (END), demonstrates the scale of the problem: over 113 million individuals, constituting approximately 20 % of the population, reside in areas exposed to transportation noise (L_den_) exceeding 55 dB [1]. However, this noise mapping only includes agglomerations with >100,000 inhabitants and areas along major roads, railways, and airports outside of urban centers, so this number is highly likely an underestimation [1].

In 2018, an expert panel appointed by the World Health Organization (WHO) published a report summarizing the evidence up to the year 2015 of the effect of environmental noise on various health outcomes [2]. They concluded that there was ‘high-quality evidence’ to support an association between road traffic noise and ischemic heart disease (IHD), with a relative risk (RR) of 1.08 (95 % confidence interval (CI): 1.01; 1.15) per 10 dB higher noise. For railway and aircraft noise, the quality of evidence was ranked as low to very low. When evaluating other cardiometabolic diseases as well as various other outcomes, such as sleep, birth outcomes, and mental and cognitive health, the WHO expert panel concluded that the evidence was of very low to moderate quality, primarily due to the scarcity of cohort and case-control studies on transportation noise and incident disease. Since 2015, available evidence has increased substantially, particularly from studies investigating the effects of road traffic noise on incident stroke and type 2 diabetes as well as cardiovascular mortality [[3], [4], [5], [6], [7]]. Also, newer studies have suggested that environmental noise may be a risk factor for diseases not evaluated by the WHO expert panel, such as heart failure, breast cancer, and tinnitus [[8], [9], [10], [11], [12]].

While mechanistic studies on noise-induced damage in humans are scarce, a substantial number of animal studies have provided deep mechanistic insights [13]. Preclinical research has identified the activation of inflammatory cells, the formation of reactive oxygen species (ROS), and oxidative damage as significant drivers of noise-associated health complications. Studies in animals have also confirmed human data on noise-triggered stress response pathways [14,15] and reduced sleep quality with dysregulation of the circadian clock [16]. These central disease-relevant pathophysiological mechanisms will be addressed briefly in the subsequent section.

With this position paper, we aim to provide an overview of the latest epidemiological research on the health effects of transportation noise. We also take a position on the urgent need for action for better population protection. Furthermore, we provide a detailed description of key publications within each specific outcome area (summarized in Table 1) to highlight important findings and exemplify high-quality study designs in estimating the health effects of transportation noise. The second part of the review highlights pathophysiological mechanisms linked with noise-triggered chronic disease, primarily based on evidence from experimental preclinical studies. The mechanistic part focuses on oxidative stress and adverse redox signaling, particularly in the cardiovascular system and the brain. Overall, we highlight the important contribution of noise to the exposome, which represents the sum of all environmental exposures with the associated biochemical changes and health outcomes across the entire lifespan [17].

**Table 1:** Summary of design and findings in key epidemiological studies.

Key epidemiological studies	Disease investigated	Summary of findings
**Pyko et al, 2023** [63]	Ischemic heart disease	Pooled analyses were performed based on nine cohorts from Denmark and Sweden, together including 132,801 subjects, with 22,459 and 7682 cases of ischemic heart disease (IHD) and myocardial infarction, respectively, identified during follow-up. The HR for IHD was 1.03 (95 % CI: 1.00, 1.05) per 10 dB L_den_ for both road and railway noise exposure. Higher risks were indicated for IHD excluding angina pectoris cases, with HRs of 1.06 (1.03, 1.08) and 1.05 (1.01, 1.08) per 10 dB L_den_ for road and railway noise, respectively. Corresponding HRs for myocardial infarction were 1.02 (0.99, 1.05) and 1.04 (0.99, 1.08). Increased risks were observed for aircraft noise but without clear exposure-response relations. A threshold at around 55 dB L_den_ was suggested in the exposure-response relation for road traffic noise and IHD.
**Thacher et al, 2022** [8]	Heart failure	A nationwide study covering Denmark consisting of 2.5 million individuals older than ≥50 years, of whom 79,358 cases developed heart failure during follow-up (2005–2017) found 10-y time-weighted road traffic noise at the most and least exposed façades to be associated with HRs (95 % CI) of, respectively, 1.039 (1.033; 1.045) and 1.087 (1.073; 1.101) per 10 dB. The exposure-response curve indicated elevated risks for L_den_Max from around 50 dB and up. People exposed to >45 dB of both road, railway and aircraft noise had highest HRs.
**Roswall et al, 2021** [4]	Stroke	In a pooled cohort of 135,951 participants from seven Swedish and two Danish cohorts with harmonized data on transportation noise, stroke, and confounders, 11,056 cases developed stroke during follow-up. Road traffic noise (L_den_, 5-year) was associated with a HR of 1.06 (1.03; 1.08) per 10 dB after adjustment for SES and 1.05 (1.02; 1.07) after further adjustment for lifestyle and BMI. Adjustment for air pollution did not change the HR. No clear associations were observed for aircraft and railway noise.
**Saucy et al, 2021** [106]	Acute CVD mortality	A case-crossover study including all deaths due to cardiovascular causes (N = 24,886) that occurred around Zurich Airport (Switzerland) between 2000 and 2015. The odds for nighttime cardiovascular mortality significantly increased with higher noise levels in the 2 h prior to the event (2h-L_Aeq_). With <20 dB 2h-L_Aeq_ as reference group, the OR at 40–50 dB 2h-L_Aeq_ was 1.33 (95 % CI: 1.05; 1.67), and >50 dB 2h-L_Aeq_ OR was 1.44 (95 % CI: 1.03; 2.04). P for trend was 0.01, indicative of a linear exposure-response relationship. No association was observed concerning daytime deaths.
**Vienneau et al, 2022** [6]	CVD mortality	A census-based, nationwide cohort study from Switzerland of 4.14 million individuals aged ≥30 years followed from 2000 to 2015, during which period 277,506 CVD deaths were accrued. Cause-specific mortality was studied. In multi-exposure models (including all noise sources and PM_2.5_) the HRs (per 10 dB L_den_) for road traffic were all elevated at 1.029 (1.024–1.034) for CVD and 1.034 (1.027–1.042) for MI mortality. HRs were similar for BP-related, IHD, and ischaemic stroke, and lower but still significant for heart failure, and stroke. Associations for railway noise were generally weaker, at 1.013 (1.010–1.017) for CVD and 1.021 (1.015–1.027) for MI mortality, with BP-related, IHD and stroke also statically increased. Aircraft noise was only clearly associated with MI at 1.040 (1.020–1.060) and ischemic stroke mortality at 1.065 (1.021–1.111). Most associations did not differ from linear, and often started below 40 dB L_den_ for road traffic and railway noise. Each outcome was also independently associated with higher levels of noise intermittency, evaluated using intermittency ratio (IR%), most strongly for heart failure (1.053 (1.050–1.055) for IR ≥ 75 % (4th quintile) vs. <25 % (reference, 1st quintile).
**Thacher et al, 2021** [115]**, Sørensen et al., 2023** [117]	Type 2 diabetes	A nationwide cohort study from Denmark of 3.56 million persons and >230,000 cases found noise to be associated with higher risk of type 2 diabetes, with HRs of 1.05 (1.04, 1.05) and 1.09 (1.08, 1.10) for road traffic noise at the most and least exposed façade, respectively, and 1.03 (1.02, 1.04) and 1.02 (1.01, 1.03) for railway noise at the most and least exposed façade, respectively. Exposure-response curves starting from 35 to 40 dB indicated no threshold below which noise was not harmful. A prospective cohort study based on 286,151 persons and 7574 type 2 diabetes cases found that lifestyle adjustment in analyses already adjusted for key sociodemographic covariates only resulted in small changes in risk estimates.
**Cantuaria et al, 2021** [132]	Dementia	In a Danish cohort of ≈2 million participants >60 years, 103,500 developed dementia during a 14-year follow-up period. A 10-year mean exposure to road traffic and railway noise at the most and least exposed façades was found to be associated with a higher risk of all-cause dementia. These associations showed a general pattern of higher HRs with higher noise exposure, and a levelling off or even small declines in risk at high noise levels.
**Sørensen et al, 2021** [10]	Breast cancer	A nationwide Danish cohort study of 1.8 million women of whom 66,006 developed breast cancer during follow-up. In fully adjusted models, road traffic noise at the most and least exposed façades were associated with HRs of 1.012 (1.002; 1.022) and 1.032 (1.019; 1.046), respectively, and railway noise at the most and least exposed façades were associated with HRs of 1.020 (1.001; 1.039) and 1.023 (0.993; 1.053), respectively. For road L_den_Min, a threshold at around 50 dB was indicated in the exposure-response relation.
**Cantuaria et al, 2023** [11]	Tinnitus	In a nationwide cohort based in Denmark of people older than 30 years, 40,692 persons were diagnosed with tinnitus. Exposure to 10-year mean road traffic noise at the least exposed façade was associated with a HR of 1.06 (1.04, 1.08), whereas for L_den_Max the corresponding HR was 1.02 (1.01; 1.03). Railway noise was not associated with tinnitus.
**Aasvang et al, 2023** [179]	Burden of disease	The burden of disease due to road traffic and railway noise was estimated in terms of Disability-adjusted Life years (DALYs) for the Nordic countries, Denmark, Finland, Norway and Sweden and their capital cities. The estimations were based on noise exposure data from the European Environmental Noise Directive (END) in addition to nationwide noise models which were available for Denmark and Norway. Noise annoyance, sleep disturbance and ischemic heart disease were included as the main health outcomes, using exposure-response functions from the WHO 2018 systematic reviews. Due to methodological differences when assessing noise exposure according to END, no comparable burden of disease estimates could be provided for the entire countries, only for the capital cities. For road traffic noise, the DALY rates for the capitals ranged from 329 to 485 DALYs/100,000 and increased with up to 17 % upon inclusion of stroke and diabetes. From 44 to 146 DALYs/100,000 could be attributed to railway noise. The DALY estimates based on nationwide noise data were 51 and 133 % higher than the END-based estimates, for Norway and Denmark, respectively, demonstrating a large underestimation of attributable burden based on END noise data.

### 1.2 Central pathomechanisms

**Fig. 1:**
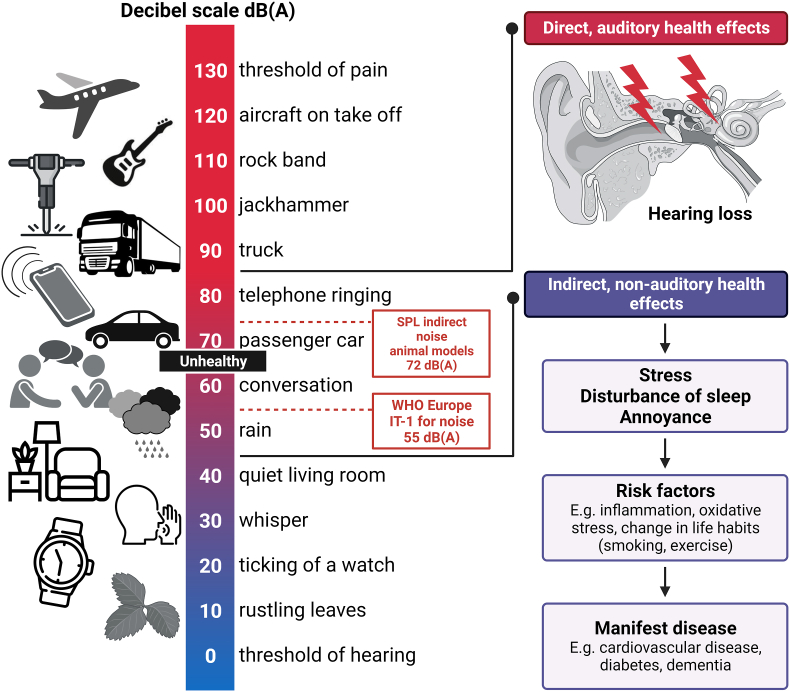
**Different noise sources and levels and their adverse health effects as envisaged by epidemiological data.** Sound pressure levels (SPL) of different noise sources leading to adverse health outcomes via the direct and indirect pathways suggested by Babisch. Modified from Münzel et al. [22] with permission of Elsevier. The icons in the figure were partially taken from Flaticon.com. The figure was created using BioRender.com.

While a link between the environment and various diseases was established decades ago, the field has continued to refine our understanding of risks that impact disease burden, including air [18] and noise pollution [13]. Specifically, environmental and lifestyle risk factors are intimately tied to cardio- and cerebrovascular disease [19]. Several studies have shown that noise below the level that induces direct physical damage can increase the risk of various diseases, most likely through the pathway proposed by Wolfgang Babisch in the ‘noise reaction model’ (Fig. 1) [20]. Babisch proposed that noise could work through an ‘indirect pathway’ to elicit subconscious stress responses and noise annoyance that in turn exacerbate risk factors and could lead to the development of cardiovascular disease (CVD), such as myocardial infarction (MI), heart failure, persistent hypertension, arrhythmia, and stroke [21,22]. Noise can also disturb sleep, ‘hijacking’ a pathway that increases the risk of ischemic heart disease (IHD) [23] and atrial fibrillation [24].

**Fig. 2:**
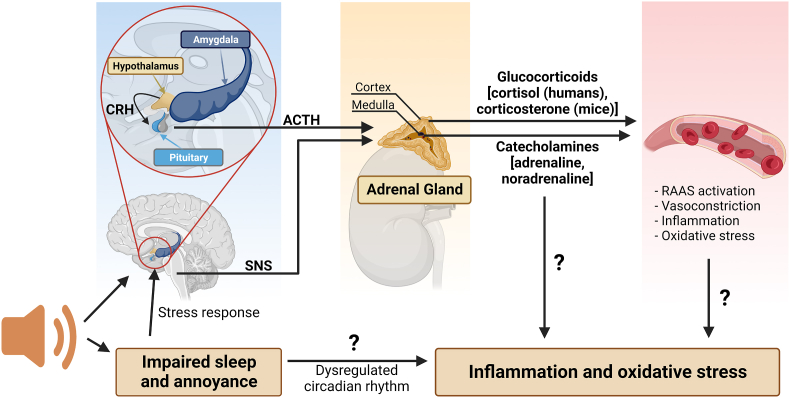
**Pathophysiology of noise-induced cardiovascular and brain disease.** Neuronal activation (arousal) induced by noise exposure triggers signaling via the hypothalamic–pituitary–adrenal (HPA) axis and sympathetic nervous system (SNS) [13,44]. The release of glucocorticoids and catecholamines in turn leads to the activation of other neurohormonal pathways (such as the renin–angiotensin–aldosterone (RAAS) system) as well as to dysregulated circadian rhythm (altered expression of central clock genes such as period 1, cryptochrome 1 and Bmal1/Arntl) and increased inflammation and oxidative stress, which can ultimately have adverse effects on cardiovascular function and molecular targets [29,45]. Alternatively, there may be a direct impact of noise-induced sleep disorders on inflammation and oxidative stress. The image was created using Biorender.com.

Arousal caused by noise activate physiological stress response systems, namely the hypothalamic–pituitary–adrenal (HPA) axis and the sympathetic nervous system (SNS). The mediators of these pathways are cortisol and catecholamines, respectively (Fig. 2), which can then subsequently activate the renin–angiotensin–aldosterone system (RAAS) and have immediate effects on the cardiovascular system, including increase in heart rate and vasoconstriction [25,26]. Although not yet proven in humans, there is some evidence that living in close proximity to major roadways is associated with higher left ventricular mass, which may be due to air pollutants or another component of roadway proximity, such as noise [27]. The connections between HPA, SNS, and RAAS activation and inflammation and oxidative stress in the vasculature and brain have been reviewed elsewhere [28,29]. One end-product of RAAS is angiotensin II, a potent (transient) vasoconstrictor and vascular regulator with well-acknowledged inflammatory and pro-oxidative properties. Angiotensin II activates circulating monocytes, which then increase circulating levels of interleukin (IL)-1β, IL-6, and reactive oxygen and nitrogen species [14,29,30]. Through this mechanism, stressors can lead to arterial hypertension and blunted endothelial function linked with increased oxidative stress and impaired nitric oxide bioavailability [31]. Over time, this can result in a super-sensitivity of vessels to stress hormone-induced vasoconstriction [32]. In addition, angiotensin II also causes cardiac hypertrophy and medial thickening in hypertensive mice, directly by effects on cell growth factors and indirectly by pressure overload [33,34], a property also shared by endothelin-1 [35,36]. Structural remodeling and hypertrophy induced by these vasoconstrictors contributes to the development of heart failure [37,38].

Notably, chronic oxidative stress and low-grade inflammation also represent pathomechanistic hallmarks of diabetes [39,40], cancer [41], and neurodegenerative diseases [42], making these adverse processes central disease-drivers in the majority of non-communicable diseases. The underlying mechanisms of noise-induced stress reactions, development of cerebrovascular inflammation, and oxidative stress are discussed in detail in the second part of this position paper (reviewed previously [29,43]). There, we highlight the important contribution of impaired circadian rhythm, stress response, inflammation, and oxidative stress to the effects of transportation noise on disease development (Fig. 2).

**Fig. 3:**
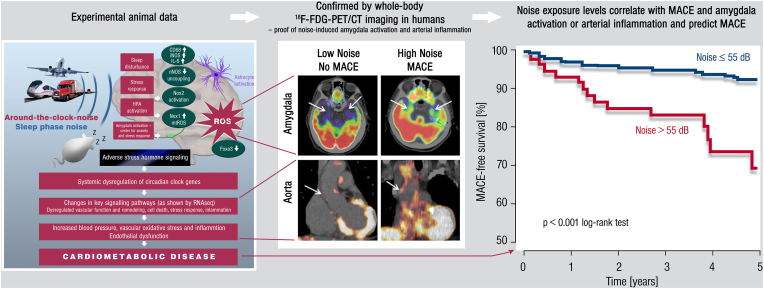
**Key data on health effects of noise through the brain-heart/vessel-axis.***Left panel:* Main results of animal studies regarding brain-heart/vessel interaction. *Middle and right panel:* Proof-of-concept translational study in humans demonstrating the association between transportation (road and aircraft) noise-induced cerebral (amygdala relative to cortical) metabolic activity and arterial inflammation increasing major adverse cardiovascular events (MACE) [47,48]. Reused with permission from Ref. [48].

A 2020 study was designed to address the neurobiological link between noise exposure, inflammation, and major adverse cardiovascular events (MACE). Stress-associated neural activity (as the ratio of amygdala to regulatory cortical metabolic activity) and the degree of arterial (aortic) inflammation was quantified in 498 healthy adults without active cancer or clinical CVD by evaluating clinical ^18^F-fluorodeoxyglucose positron emission tomography-computed tomography (PET–CT) imaging [46]. In this study, increased noise exposure at the individuals home address was independently linked with metabolic activity of the amygdala (relative to regulatory cortical activity), arterial inflammation, and a higher risk of MACE after accounting for air pollution, socioeconomic factors, and established CVD risk factors. Analyses indicated that higher noise exposure was associated with MACE via heightened amygdala activity and arterial inflammation (Fig. 3) [47,48]. Notably, the same pathway has also been implicated in the link between perceived stress and socioeconomic disparities (e.g., lower education or income) and CVD [49,50].

### 1.3 Health effects of exposure to traffic noise in humans

In the following section, we provide an overview of some of the key scientific advancements since 2015, focusing on cardiometabolic diseases as well as diseases that are emerging in a noise context, with emphasis on results from cohort and case-control studies. We will touch upon the importance of conducting a valid noise exposure assessment, having a sufficient number of observations, and applying an extensive confounder control, as these are prerequisites for achieving reliable results. As the shape of the exposure-response function for transportation noise and disease is crucial for health impact assessment, we provide detailed descriptions of key papers that report such data, estimated based on assessment of noise levels throughout the exposure span. Recent studies have investigated health effects using a “new” noise indicator - noise at the least exposed façade (L_den_Min) - in addition to noise at the most exposed façade (L_den_Max), corresponding to the noise indicator used in most previous studies. As people often select a bedroom facing away from a busy road (if possible), L_den_Min is hypothesized to be a proxy of bedroom noise exposure, thus better capturing exposure during sleep. This is important because health effects of noise are believed to be partially mediated through sleep disturbance [51,52]. We describe some of the key studies assessing effects of L_den_Min. A definition of these and other important noise metrics are provided in Textbox 1.

Textbox 1 **Key noise definitions and metrics**

**The sound pressure level** (SPL) is the pressure level of a sound measured using the logarithmic decibel scale (dB). Most studies estimate or measure A-weighted SPL (dB(A)). The A-weighting (where A relates to the international normalized frequency rating curve) is employed to accommodate varying sensitivity of the human ear across different sound frequencies.

**L**_**Aeq**_ is the A-weighted, equivalent ("eq") sound pressure level, corresponding to the average received sound energy (A-weighted) over time. Commonly used L_Aeq_'s include: **L**_**Aeq,24h**_ (covers an entire day, 24 h), **L**_**day**_ (07:00 to 19:00), **L**_**evening**_ (19:00 to 23:00), and **L**_**night**_ (23:00 to 07:00). The L_Aeq_ is often calculated as annual average noise levels**.**

**L**_**den**_ is the L_Aeq_ over 24 h with a penalty of 10 dB(A) for nighttime noise (23.00–07.00, **L**_**night**_) and a penalty of 5 dB(A) for evening noise (19.00–23.00, **L**_**evening**_). These penalties are used to capture higher sensitivity to noise exposure during the evening and the night. L_den_ is often calculated as average noise levels over 1-, 5- and/or 10-years in research studies. L_den_ and L_night_ are the noise indicators that are used in strategic noise mapping according to The Environmental Noise Directive, 2002/49/EC (END).

**L**_**den**_**Max**. L_den_ is commonly estimated at the most exposed façade of a building/residence. The term L_den_Max specifies that the L_den_ is estimated at the most exposed façade, and in many scientific papers and reports, L_den_Max and L_den_ are the same.

**L**_**den**_**Min** is the estimation of L_den_ at the least exposed façade of a building/residence. L_den_Min is a relatively new noise metric in environmental research. It is hypothesized to be a proxy of bedroom noise, thus better capturing exposure during sleep than L_den_Max.

Alt-text: Textbox 1

#### 1.3.1 Ischemic heart disease incidence

The most comprehensive human evidence on adverse health effects of transportation noise, besides annoyance and sleep disturbances, relates to IHD. IHD includes acute myocardial infarction (MI) and angina pectoris, which share a similar pathophysiology and contribute to heart failure. MI is the most common outcome studied in relation to transportation noise and has the advantage of clear diagnostic criteria and a high probability of hospital care, leading to very good coverage in patient registries.

The only epidemiological evidence on cardiovascular effects that was judged by the WHO in 2018 to be of high quality was the association between road traffic noise and incidence of IHD [2]. Seven longitudinal studies from Europe were included in the quantitative assessment, primarily based in large cities, such as Berlin, Bristol, Copenhagen/Aarhus, and Stockholm [2]. The weighted mean road traffic noise level in the reference category in the studies was 53 dB L_den_ and the association exceeding this level appeared approximately linear with a RR of 1.08 (95 % CI: 1.01; 1.15) per 10 dB L_den_. The majority of the studies focused on MI, thus, it is uncertain to what extent this risk estimate also applied to other types of IHD.

Several studies on road traffic noise and MI/IHD have been published after the WHO meta-analysis. One systematic review and meta-analysis on MI focused on 13 studies, including those in the WHO review, comprising a total of seven cohort studies, five case-control studies and one cross-sectional study [53]. Excluding one conference report, the overall RR per 10 dB L_den_ was 1.03 (95 % CI: 1.00; 1.05), with significant heterogeneity between the studies. More recent findings, not included in the two reviews, also indicated lower risk estimates than in the WHO review or no clear associations [[54], [55], [56], [57], [58]]. All but one of these studies were strictly registry-based and did not contain any information on lifestyle, e.g., smoking, increasing the risk of residual lifestyle confounding compared to the studies in the two reviews, which generally included such data. A particular issue in relation to confounding for road traffic noise concerns air pollution, i.e., fine particulate matter (PM_2.5_), which is a risk factor for CVD. Several of the studies on road traffic noise and MI/IHD were adjusted for air pollution which led to attenuation of the associations in some cohorts. However, a recent systematic review of 52 studies concluded that there was little evidence for a confounding effect of air pollution on CVD [59]. While the review also concluded that noise associations are mostly not confounded by air pollution, more studies investigating potential interactions between noise and air pollution are needed to investigate whether there are intertwined health effects and pathophysiological mechanisms, as suggested by other reviews [60,61]. A cumulative effect on risk for MI by noise, air pollution and lack of green space was recently published [62].

**Fig. 4:**
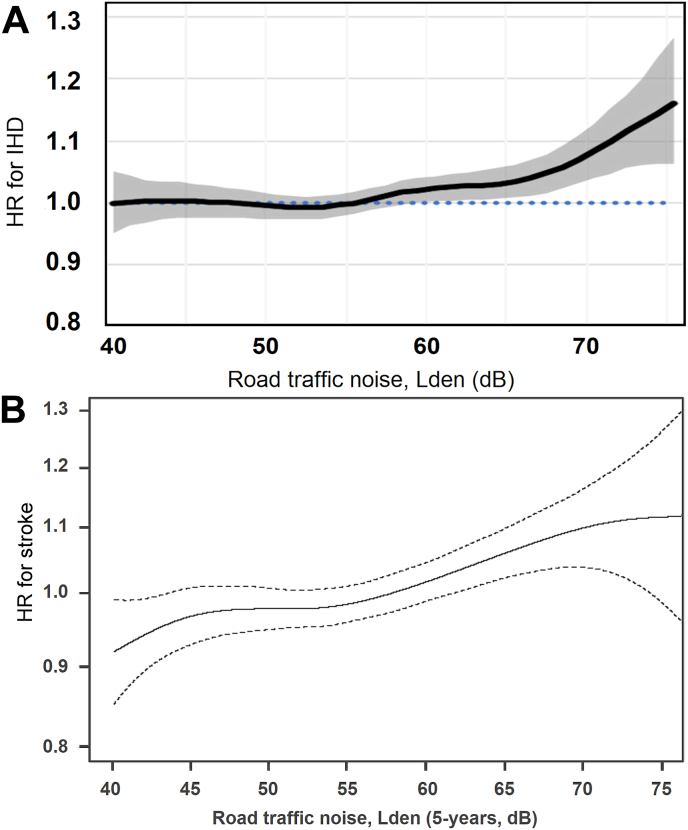
Association between 5-year mean exposure to road traffic noise and risk of incident ischemic heart disease (IHD) (**A**) and stroke (**B**) in pooled analyses based on nine cohorts from Denmark and Sweden including ≈130,000 participants. Reused from Refs. [4,63] with permission.

Most studies on road traffic noise and MI/IHD did not make a detailed evaluation of exposure-response relationships. However, this was assessed in a pooled analysis of nine cohorts from Denmark and Sweden [63]. Several cohorts in this pooled study were included in the two reviews mentioned above [2,53], but longer follow-up periods resulted in a substantially greater number of cases in the pooled analysis. The adjusted hazard ratio (HR) for IHD was 1.03 (95 % CI: 1.00; 1.05) per 10 dB L_den_ road traffic noise exposure during five years prior to the cardiovascular event. A higher risk was indicated for IHD excluding angina pectoris cases, with a corresponding HR of 1.06 (95 % CI: 1.03; 1.08), while it was 1.02 (95 % CI: 0.99; 1.05) for MI. A threshold of around 55 dB L_den_ was proposed in the exposure-response relation for road traffic noise and IHD (Fig. 4). Such a threshold in the exposure-response function may contribute to explaining the lower risk estimates in studies published after the WHO review, as these studies often had a lower proportion of persons exposed to high levels of road traffic noise [64].

**Fig. 5:**
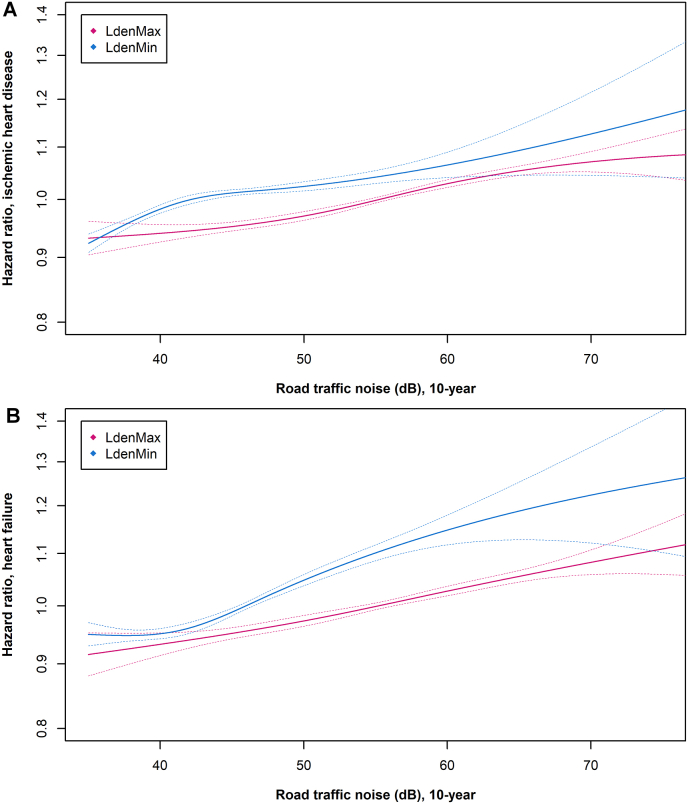
Association between 10-year mean exposure to road traffic noise at the most (pink line) and least (blue line) exposed façade and risk of (**A**) ischemic heart disease and (**B**) heart failure in a cohort covering entire Denmark (2.5 million persons ≥50 years). Graphs created *de novo* based on the population described in Ref. [8].

The studies on road traffic noise and MI/IHD were generally based on estimated noise levels at the most exposed façade (L_den_Max). Only one study investigated risks of MI and IHD in relation to estimated noise levels at the least exposed façade (L_den_Min) [8]. This study was based on a nationwide Danish cohort, using information from registries. The HR for IHD was 1.05 (95 % CI: 1.04; 1.06) per 10 dB 10-year mean road L_den_Min. Corresponding risk estimates for MI and angina pectoris were 1.03 (95 % CI: 1.02; 1.05) and 1.11 (95 % CI: 1.08; 1.14), respectively. The risk estimates for road L_den_Max were similar to those for L_den_Min, and the exposure-response relation indicated a threshold of around 50 dB (Fig. 5).

Few studies have addressed risks of MI/IHD concerning exposure to railway or aircraft noise, probably because these exposures affect a relatively small proportion of the general population, making risk estimates uncertain. In three large studies, risk estimates for MI related to railway noise were 1.02 (95 % CI: 1.01; 1.04) [65], 0.97 (95 % CI: 0.95; 0.99) [8] and 1.04 (95 % CI: 0.99; 1.08) [63] per 10 dB L_den_, respectively. These three studies also investigated effects of aircraft noise, suggesting an increased risk of MI. In a small fourth study no association with aircraft noise was observed [54]. Two studies indicated that combined exposure to all three kinds of transportation noise (road traffic, railway and aircraft) may bring a risk of IHD [8,66].

In conclusion, there is strong evidence that long-term exposure to road traffic noise is associated with an increased risk of incident IHD, including MI. However, the excess risk appears lower in recent studies compared to the estimate calculated for the WHO report, which may be ascribed to relatively fewer individuals exposed to high noise levels and thresholds in the exposure-response function. An increased risk probably also exists in persons exposed to railway and aircraft noise, but the data is too limited for precise risk estimation.

#### 1.3.2 Heart failure incidence

Heart failure is one of the leading causes of morbidity and mortality worldwide. It is characterized by symptoms such as shortness of breath, structural or functional cardiac abnormalities and by reduced cardiac output caused by either impaired systolic or diastolic function (in general, the heart's inability to pump blood efficiently). One consequence of chronic noise exposure is activation of the SNS, leading to an increase in blood pressure and elevated heart rate, which can induce structural vascular changes and over time result in heart damage [67,68]. To date, only a handful of studies have investigated the association between transportation noise and heart failure, and heart failure was not evaluated by WHO in 2018 [69].

Currently, six longitudinal studies have investigated the association between transportation noise and the incidence of heart failure [8,55,[70], [71], [72], [73]]. Studies yield consistent positive associations between road traffic noise and heart failure, ranging from 2 to 9 % higher risk per 10 dB [8,55,[70], [71], [72], [73]]. While the two studies on railway noise and heart failure both indicated positive associations [8,71], the two studies on aircraft noise and heart failure were contradictory: a Danish nationwide study reported a positive association [8] while a large German study found no overall association [71].

The most recent of the aforementioned studies was a nationwide cohort from Denmark with a study base of around 2.5 million persons above 50 years of age and 79,358 incident cases of heart failure. The study reported an association with a 4 % higher risk of heart failure per 10 dB road traffic noise [8]. A novel aspect and important strength of this study was the inclusion of noise at the least exposed façade (L_den_Min), which was associated with a higher risk for heart failure compared to noise at the most exposed façade (L_den_Max). This was the case for both road and railway noise (e.g., for road traffic noise the HRs per 10 dB were 1.04 (95 % CI: 1.03; 1.05) for L_den_Max and 1.09 (95 % CI: 1.07; 1.10) for L_den_Min). Thacher and colleagues also reported that combined exposure to multiple noise sources (road, rail, or aircraft) was particularly harmful, with a HR of 1.27 (95 % CI: 1.17; 1.37) in people exposed to all three noise sources.

Few studies have investigated the shape of the exposure-response function for transportation noise and heart failure. In the Danish nationwide study, a clear exposure-dependent association was seen for road traffic noise, with elevated risk for heart failure evident already at around 50 dB for L_den_Max and 45 dB for L_den_Min (Fig. 5) [8]. Lastly, Thacher et al. found that road and railway noise models were robust to adjustment for PM_2.5_.

Taken together, the studies published to date consistently point towards transportation noise as a risk factor for incident heart failure, particularly for road traffic noise. However, further well-designed longitudinal studies are still needed, especially to elucidate to what extent railway and aircraft noise affects the risk of developing heart failure.

#### 1.3.3 Stroke incidence

Stroke is one of the leading causes of death and disability worldwide [74]. When the evidence was compiled for the 2018 WHO noise guidelines, only one cohort study on the effects of noise on stroke incidence was available [69]. The Danish study found an HR of 1.14 (95 % CI: 1.03; 1.25) per 10 dB L_den_ increase in road traffic noise [75] and the WHO rated the evidence as being of moderate quality. At that time there were only a few ecological and cross-sectional studies that addressed the impact of railway and aircraft noise, and the evidence was rated as very low quality for both.

The number of studies on road traffic noise and incident stroke has increased substantially in recent years. In five of the new studies, confounder adjustment has been thorough (i.e., lifestyle factors and/or individual level socioeconomic status as well as air pollution have been accounted for). Two of the studies, based on data from nine pooled Scandinavian cohorts and the entire Danish population, respectively, found road traffic noise to be associated with a higher risk of stroke [4,5]. Studies based on cohorts in London [72], Norway and Oxford [76], and the United Kingdom as a whole [77] found no associations in the fully adjusted models. However, it should be noted that simplified noise exposure assessment approaches were applied in the studies which found no association. In studies with less complete confounder adjustment, two studies reported an association with stroke [56,78], whereas three other studies did not [54,58,79].

There have been only a few new longitudinal studies on railway and aircraft noise. Two studies reported no association between railway noise and stroke [4,5], whereas one study with less complete confounder adjustment did [78]. The same study did not find any association between daily mean aircraft noise level and stroke, but there were indications that nighttime noise events might be harmful. One study on aircraft noise had only five cases [54], rendering the results uninformative, and another study found an association at moderate but not high noise levels [4].

A pooled study of nine cohorts in Denmark and Sweden is a recent example of a study applying both valid noise exposure assessment, sufficient observations, and extensive adjustment for confounders [4]. This study included over 135,000 participants and 11,000 stroke cases and adjusted for individual and area-level confounders. It assessed exposure using the Nordic prediction method accounting for full residential history at address-level precision. The study found road traffic noise to be associated with a higher risk of stroke, with an HR of 1.06 (95 % CI 1.03; 1.08) for each 10 dB increase in L_den_, and the association remained after adjustment for air pollution. There was no difference between the effect estimates for two stroke subtypes (i.e., ischemic and hemorrhagic). Railway noise was not associated with stroke, and the results for aircraft noise were inconclusive.

The exposure-response function for road traffic noise in the pooled Scandinavian study was approximately linear from 40 dB to 80 dB (Fig. 4). Similarly, a large nationwide Danish study found that the association was seemingly linear at lower levels of noise (from 40 dB), although the effect seemed to level off at higher levels (roughly above 62 dB) [5]. HR in the study was 1.04 (95 % CI: 1.03; 1.05) per 10 dB road L_den_Max. The effect estimate for road L_den_Min was comparable.

In summary, the number of studies on road traffic noise and stroke incidence has substantially increased in recent years. Although large studies of high quality regarding exposure, confounders, and outcome assessment reported adverse effects, the inconsistent findings relating to road traffic noise and stroke call for more research based on high-quality prospective cohort studies. For railway and aircraft noise there are too few studies to draw conclusions. Of note, a cumulative effect on risk for stroke by noise, air pollution and lack of green space was recently published [80].

#### 1.3.4 Cardiovascular mortality

Chronic exposure to transportation noise and its effects on the body can influence the progression of CVD and ultimately lead to death. Reflecting the available body of evidence at the time, mortality studies in the 2018 WHO review [69] related only to IHD and stroke, in relation to road traffic [[81], [82], [83], [84]] or aircraft noise [[85], [86], [87]]. No studies were available on railway noise. For IHD mortality, the pooled estimates per 10 dB L_den_ were 1.04 (95 % CI: 0.97; 1.12) for aircraft noise and 1.05 (95 % CI: 0.97; 1.13) for road traffic noise. Only aircraft noise exposure showed a trend towards an association with stroke mortality: 1.07 (95 % CI: 0.98; 1.17). Overall, the number of studies was limited in number and scope with studies mainly from Europe. For aircraft noise, the majority were ecological studies and later judged to have ‘low-quality evidence’. For road traffic noise, however, the judgment was deemed ‘moderate-quality evidence’.

Newer mortality studies have included a broader range of specific CVDs. Those showing an association of incident CVD with noise were mainly the larger cohort studies (predominantly from Denmark and Switzerland), which not only followed participants for decades, but had the highest quality exposure assessment at the home's façade [6,[88], [89], [90]]. This has been demonstrated to be essential for minimizing exposure measurement error [91]. In these studies, the associations between noise and CVD mortality were also robust to air pollution adjustment [6,8,92,93]. Unique features of the Danish studies included: the long address record allowing exposure to be explored over different long-term averaging periods (e.g. as 1, 5, 10 and 23-year means depending on the study) [88,90] and exposure for both the most and least exposed façades [90,91]. Similarly, the Swiss studies offer unique insights into the timing of noise exposure over the 24-h day [52] and the influence of other noise characteristics such as intermittency [6,89].

A Danish cohort study, including roughly 53,000 individuals, reported the risk of all CVDs and stroke mortality to be 1.13 (1.06; 1.19) and 1.11 (0.99; 1.25), respectively, per IQR 10-year mean road L_den_Max and 1.10 (1.01; 1.21) for IHD for L_den_Min, after considering important lifestyle factors not often available in all large cohorts [90]. The Swiss National Cohort, effectively including all adults in Switzerland but lacking lifestyle factors, studied these relationships plus mortality from blood pressure-related disease, MI, and heart failure, finding small (2–4%) increased risks for each condition in relation road traffic noise (e.g., 1.03 (1.02–1.03) per 10 dB L_den_Max for CVD mortality) [6]. Railway noise was also associated with all CVDs, blood pressure-related, IHD, MI, and stroke mortality but not with heart failure. Higher levels of intermittency at night were independently associated with mortality. Another Danish study with detailed lifestyle data on ≈25,000 female nurses did not find significant associations between road traffic noise and all CVD, stroke or IHD (e.g., 1.10 (0.91–1.31) per 10 dB road traffic noise for stroke mortality) [88]. Two small studies on road traffic noise and all CVD mortality exclusively in men were conducted in Caerphilly, South Wales, UK (n = 2398) and Gothenburg, Sweden (n = 6304) and did not find any associations [94,95]. In addition, two large studies from the UK (n ≈ 340,000) and the Netherlands (n ≈ 340,000) found no associations with road traffic noise [77,96]. In the UK study, the association between road traffic noise and CVD mortality attenuated to null after adjusting for air pollution, and the Dutch study found no association with either road traffic noise, railway noise, or air pollution. The latter observation suggests that the study suffered some methodological constraints, as the link with air pollution and CVD is well-established.

Few newer studies have investigated CVD mortality in relation to aircraft noise; only two based on cohorts with individual-level data investigated CVD mortality in relation to aircraft noise: the US nurses cohort [97] and the Swiss National Cohort study [6]. Neither found an association with all CVD mortality, though the Swiss study did show increased risk for mortality specifically from MI and ischemic stroke (1.04 (1.02–1.06) and 1.07 (1.02–1.11) per 10 dB L_den_, respectively after co-exposure adjustment). Exposures were generally low in the US study, and the exposure contrast was small. Interestingly, in Switzerland, the association between aircraft noise exposure and CVD mortality were stronger and exhibited a linear increase from as low as 30 dB when focusing on the populations in the immediate vicinity of airports: 1.02 (1.01–1.03) and 1.06 (1.02–1.09) per 10 dB L_den_ for CVD and MI mortality, respectively [98].

In conclusion, road traffic noise shows associations with all CVD and IHD/MI mortality and is judged to be of moderate-high quality. Studies on railway and aircraft noise are still too few to judge, though indicate only a small increased risk for all CVD mortality, if any, based on moderate quality evidence.

#### 1.3.5 Short-term cardiovascular health effects of noise in a population setting

Investigating short-term or acute health effects of transportation noise on any health outcome in epidemiological studies is notoriously difficult due to a variety of methodological challenges. First, to study short-term effects, fine resolution time information on both the exposure and the outcome are necessary. Concerning MI, for example, exposures in the 2 h preceding the event are usually considered as possible triggers [99]. This means that to study transportation noise as a possible trigger for MI, hourly resolution noise exposure data and the exact time of the outcome event are required. Second, in many settings, noise follows regular patterns with variations in the exposure levels, primarily due to external factors influencing traffic activity, such as the time of the day, day of the week, and holidays. Since these factors also influence people's behavior and, therefore, are associated with the onset of many acute adverse health outcomes, disentangling possible acute health effects from such time trends is difficult. This mainly applies to road traffic noise, railway noise, and industry noise. Other sources, like wind turbine and aircraft noise, show a higher temporal variability, which offers opportunities to study acute health effects.

Multiple epidemiological approaches suited to studying the acute effects of exposure on transient risk changes for immediate onset outcomes exist. Time series analyses are commonly conducted in environmental epidemiology for aggregated data [100]. For data on individual level, self-matched designs, such as the case-crossover design or the more recently developed case time series design, are well-suited [101,102]. These designs have the additional benefit of adjusting for time-constant, individual-level covariates by design. So far, they have been predominantly applied to study health risks due to temperature or air pollution [103,104].

Only few methodologically robust studies on acute effects of transportation noise have been conducted and, therefore, we in this section also evaluate studies investigating acute effects of other noise-sources. In a Danish study, hospitalizations and deaths from stroke (16,913 cases) and AMI (17,559 cases) among Danes exposed to wind turbine noise between 1982 and 2013 were analyzed using a time-stratified case-crossover design [105]. Mean nighttime outdoor (10 Hz–10 kHz) and low frequency (10–160 Hz) indoor wind turbine noise was predicted for the four days preceding diagnosis and reference days. For outdoor wind turbine noise above 36 dB, there were indications of an association with stroke but not with MI. For low-frequency indoor noise between 10 and 15 dB and above 15 dB, odds ratios (ORs) (95 % CI) for MI were 1.27 (0.97; 1.67) and 1.62 (0.76; 3.45), respectively, when compared to indoor low-frequency wind turbine noise below 5 dB. For stroke, corresponding ORs (95 % CI) were 1.27 (0.95; 1.69) and 2.30 (0.96; 5.50).

One case-crossover study found evidence for short-term associations between aircraft noise exposure and CVD mortality based on an analysis of all cardiovascular deaths that occurred around the Zurich airport between 2000 and 2015 [106]. Nighttime noise 2 h preceding death among people exposed to 40–50 dB and >50 dB was associated with ORs (95 % CI) of respectively 1.33 (1.05; 1.67) and 1.44 (1.03; 2.04) compared to the reference of <20 dB with a significant exposure-response trend. No associations were observed for daytime deaths. This suggests that nighttime aircraft noise can trigger deaths by CVD. Among specific outcomes, associations indicated an increased risk for IHD, MI, heart failure, and arrhythmias.

A study around Heathrow Airport applied the same crossover approach on hospital admissions and deaths due to CVD [107]. Since only the date, and not time, of death was available, however, they could not investigate exposures directly before the events. The study found slight associations between emergency hospital admissions due to CVD and aircraft noise exposure on the previous late evening (22:00–23:00h, OR per 10 dB = 1.007 [95 % CI: 1.000; 1.013]) or in the early morning (04:30–06:00h, OR per 10 dB = 1.012 [95 % CI: 1.002; 1.021]) of the same day. No associations with cardiovascular deaths were observed. This is an example of the challenges when investigating the acute effects of noise in a population setting, and how important it is to have fine temporal resolution exposure and outcome data to do so successfully.

In conclusion, more high-quality studies on the acute health effects of transportation noise are needed. In light of the available methods and increasing availability of high-quality, fine temporal and spatial resolution noise models, the necessary tools to conduct such studies are available.

#### 1.3.6 Incidence of type 2 diabetes

Global diabetes prevalence has been on a steady rise for decades, surging from 108 million in 1980 to 422 million in 2014 [108]. Key risk factors include obesity, a sedentary lifestyle, and an unhealthy diet, and recent studies have suggested that also transportation noise may be a risk factor for type 2 diabetes [54,[109], [110], [111], [112], [113], [114], [115]].

In 2018, the expert group appointed by WHO identified only one high-quality study on transportation noise and diabetes [116], based on which they concluded moderate-quality evidence for an association [69]. Since then, nine cohort studies investigating the effects of transportation noise on the risk of incident diabetes have been published, consistently showing that noise, especially from roads, was associated with a higher risk of type 2 diabetes [54,[109], [110], [111], [112], [113], [114], [115]]. Based on these cohort studies, a 2023 meta-analysis found a joint risk estimate per 10 dB of 1.06 (1.03; 1.09) for road traffic noise (7 studies), 1.01 (1.00; 1.01) for aircraft noise (3 studies), and 1.02 (1.01; 1.03) for railway noise (2 studies) [3].

**Fig. 6:**
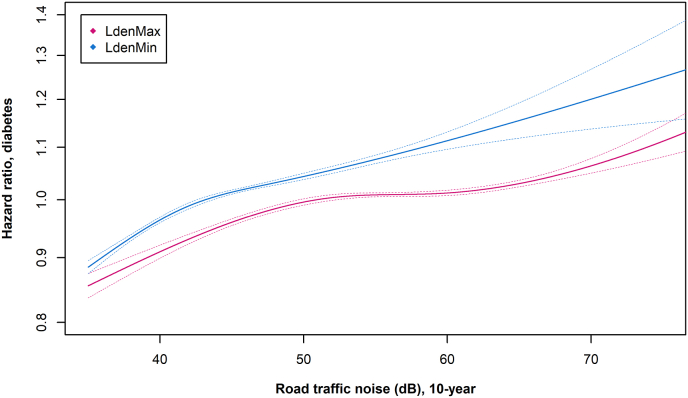
Association between 10-year mean exposure to road traffic noise at the most (pink line) and at the least (blue line) exposed façade and risk of type 2 diabetes in a cohort covering all of Denmark (3.56 million persons ≥35 years old). Graphs created *de novo* based on the population described in Ref. [3].

The study that added most weight into the meta-analysis on noise and diabetes [3], was a nationwide study in Denmark, with the inclusion of 3.56 million participants ≥35 years old and 233,912 incident cases of type 2 diabetes [115]. The study investigated the effects of long-term noise exposure (10-year mean) to road, railway, and aircraft noise, calculated based on detailed information on the moving history of all participants at address-level precision. For roads and railways, the study included both L_den_Max and L_den_Min. Lastly, the analyses adjusted for various individual- and area-level sociodemographic covariates, such as education, income, and occupation, and air pollution. The study found that road traffic and railway noise were associated with a higher diabetes risk. For road traffic noise, the association was strongest for L_den_Min, with HRs per 10 dB of 1.08 (1.07; 1.09) for L_den_Min and 1.03 (1.03; 1.04) for L_den_Max in fully adjusted models, indicating that effects of noise on sleep is an essential pathway in the development of noise-induced diabetes. The exposure-response curves for road L_den_Max and L_den_Min indicated the lack of lower “safe” noise level, as the risk increased throughout the exposure range from 35 to 40 dB and up (Fig. 6). If confirmed in future studies, this will add substantially to the estimated disease burden, as current health impact studies are based mainly on noise levels ≥55 dB L_den_.

A limitation of administrative studies, such as the above-described Danish study, is the lack of information on lifestyle factors. The degree of residual confounding from lifestyle in studies with access to only sociodemographic covariates was recently investigated in a Danish cohort study of 286,151 persons of whom 7574 developed diabetes during follow-up [117]. This study found a HR of 1.07 (95 % CI: 1.04; 1.10) per 10 dB L_den_Max in a crude model adjusted for age, sex, and year. Following adjustment for individual- and area-level sociodemographic covariates, the HR was reduced to 1.05 (95 % CI: 1.02; 1.08), indicating the importance of considering socioeconomic differences in noise studies. After further adjustment for lifestyle, more specifically smoking, consumption of fruit, vegetables and red meat, and physical activity, the HR was 1.04 (95 % CI: 1.01; 1.07). This suggests that residual confounding due to lifestyle covariates is not a major concern in registry-based studies with adjustment for key sociodemographic covariates, although these results need confirmation in future studies on other populations, both in relation to diabetes and other outcomes.

In support of noise as a risk factor for type 2 diabetes, four longitudinal cohort studies have indicated that transportation noise increases the risk of developing overweight [[118], [119], [120], [121]], which is a major risk factor for diabetes. Although the indicators of obesity investigated displayed some variation across the studies (body mass index (BMI), waist circumference, and weight gain), associations were generally observed between road traffic noise and markers of obesity. Interestingly, results on changes in waist circumference and central obesity were more consistent than results on changes in BMI [118,119]. This observation aligns with a noise-induced activation of the stress-response, as high concentrations of cortisol have primarily been associated with central obesity.

In conclusion, exposure to road traffic noise has consistently been shown associated with diabetes, whereas evidence is still lacking for railway and aircraft noise. Recent studies suggest that L_den_Min may be more relevant than L_den_Max in the development of diabetes and therefore future studies should include this measure. Importantly, a cumulative effect on risk for diabetes by noise, air pollution and lack of green space was recently published [122].

#### 1.3.7 Incidence and mortality from neurodegenerative diseases

As described above, substantial evidence linking transportation noise and cardiometabolic diseases has emerged in recent years. However, our understanding of the detrimental health effects of noise on the brain remains limited. Degenerative diseases of the brain and nervous system (e.g., Alzheimer's disease, and Parkinson's disease) affect millions of persons worldwide and are a public health priority due to their economic and societal burden [123,124].

Among well-known risk factors for neurodegenerative diseases, such as education and unhealthy lifestyle, environmental exposures like air pollution and noise have been suggested to affect the central nervous system [[123], [124], [125], [126]]. The number of studies investigating associations between transportation noise and dementia and cognition in adults is, however, limited. In the WHO guidelines from 2018, dementia was not evaluated due to lack of studies [2].

A systematic review from 2020 concluded that there was no clear evidence supporting an association between transportation noise and dementia or cognitive decline, given the few studies with high variation in outcome definition and study design [127]. Among the five studies included in this review, only two investigated transportation noise and incidence of dementia. Both studies, one from Sweden [128] and one from the UK [129], found that noise was not associated with a risk of dementia. Two cross-sectional studies included in this review, however, suggested that transportation noise can affect cognitive function in adults [130,131].

Since this review, four new studies on transportation noise and dementia have been published [88,[132], [133], [134]]. One was an American cohort study including 1612 participants, which found positive associations between road traffic noise and Alzheimer's disease, with a HR of 1.3 (95 % CI: 1.0; 1.6) per 11.6 dB (interquartile range, IQR) [133]. In a Canadian study investigating neurodegenerative diseases (i.e., non-Alzheimer's dementia, Alzheimer's disease, Parkinson's disease, and multiple sclerosis), traffic-related community noise was not associated with any of the outcomes [134]. Another study conducted in Denmark looked specifically at dementia-related mortality and found a HR of 1.12 (95 % CI: 0.90; 1.38) per 10 dB increase in 5-year mean L_den_Max [88]. The fourth and largest of these recent studies was a Danish nationwide cohort study including almost two million elderly [132]. Besides its large study population and long follow-up, this study presented some unique strengths compared to most studies on the topic. First, the exposure assessment was based on the exact address location and considered the complete address history before and throughout the entire follow-up period, which differs from other studies that estimated noise levels at postal code levels [129,134], and/or only assessed noise at one point in time [128,129,133]. Second, noise exposure was estimated at the most and least exposed façades, which allows for possible interpretations on noise exposure during sleep.

The nationwide Danish study found transportation noise from road traffic and railway to be associated with an increased risk for all-cause dementia and dementia subtypes. For Alzheimer's disease, the authors found a HR of 1.16 (95 % CI: 1.11; 1.22) for road L_den_Max ≥65 dB compared with <45 dB; and 1.27 (95 % CI: 1.22; 1.34) for road L_den_Min ≥55 dB compared with <40 dB. Road traffic noise, but not railway noise, was associated with vascular dementia. For all-cause dementia, exposure-response functions showed linear associations starting from 35 dB, with leveling-off or even small declines at high noise exposures.

Despite the limited number of studies investigating associations between transportation noise and neurodegenerative diseases, a growing body of evidence has demonstrated that transportation noise may also be detrimental to the brain and nervous system. Therefore, future studies investigating associations between transportation noise and these diseases are strongly recommended.

#### 1.3.8 Cancer incidence and mortality

Exposure to transportation noise has been associated with various risk factors for cancer, including oxidative stress, inflammation, disruption of the circadian rhythm, and change in lifestyle habits, such as smoking and alcohol intake (Fig. 1, Fig. 2) [14,67,135,136]. However, the effects of transportation noise on cancer have received only a little attention, with a total of 10–15 epidemiological studies to cover this highly diverse and prevalent disease outcome, including studies on breast and colon cancer [9,10,[137], [138], [139], [140]] and cancer mortality [7,88,90].

The most studied cancer outcome concerning transportation noise is breast cancer, which has been investigated in three Danish [9,10,137] and one German study [141]. While the three Danish studies investigated effects of long-term exposure to noise (10-year mean in two studies [10,137] and 24-year mean in one study [9], the German study only had information on noise exposure at time of cancer diagnosis. The results on breast cancer are inconsistent. A Danish cohort study of 29,875 women found both road traffic and railway noise to be associated with a higher risk of estrogen-receptor negative but not with estrogen-receptor positive breast cancer [137], which was partly supported by a large German case-control study (≈478,000 women) that indicated associations between exposure to high levels of aircraft noise only among women with estrogen-receptor negative breast cancer [141]. However, the German study found only weak indications of associations between road traffic and railway noise and the risk of breast cancer. Furthermore, a Danish cohort of 22,466 nurses found associations between road traffic noise and breast cancer only among women with estrogen-receptor-positive breast cancer [9]. The largest study of noise and breast cancer is a nationwide Danish cohort study of 1.8 million women, of whom over 66,000 developed breast cancer during follow-up [10]. The study had access to residential address history for all participants, with address-specific estimation of road traffic and railway noise at the most and least exposed façades. The authors reported that a 10 dB increase in road L_den_Min (10-year mean) was associated with an HR of 1.032 (95 % CI: 1.019; 1.046), whereas for road L_den_Max, only a slightly higher risk was observed (HR: 1.012; 95 % CI: 1.002; 1.022) in fully adjusted models, including socioeconomic status and use of hormone replacement therapy. This indicates that the effects of noise during sleep may be significant in developing breast cancer, potentially disturbing the circadian rhythm [136], which is a suspected risk factor for breast cancer [142]. The study also found railway noise associated with a slightly higher risk of breast cancer with HRs of 1.02 for both L_den_Max and L_den_Min. In contrast to previous studies, the nationwide Danish study found similar size HR among women with estrogen-receptor positive and estrogen-receptor negative breast cancer subtypes.

Another type of cancer that has received some attention in relation to transportation noise is colon cancer [[138], [139], [140]]. The studies conducted indicated that long-term exposure to road traffic noise (5- or 10-year time-weighted means) might be associated with a slightly higher risk of colon cancer with a HR per 10 dB increase of 1.011 (95 % CI: 0.997; 1.025) in a nationwide Danish cohort of 3.5 million participants and 36,000 incident cases [140] and a HR of 1.06 (95 % CI: 1.00; 1.12) in a population of 11 pooled Nordic cohorts totaling ≈155,000 persons and 2757 cases [139]. Long-term effects of road traffic noise on the risk of prostate cancer, non-Hodgkin's lymphoma (NHL), and childhood cancer have been investigated in only one study each, which suggested that high exposure to road traffic noise may be a risk factor for NHL [143], but not for prostate [144] or childhood cancer [145]. Lastly, a few studies have investigated associations between noise and overall cancer mortality, indicating associations between long-term exposure to road traffic noise (10- or 23-year time-weighted means) and overall cancer mortality with HRs ranging from 1.02 to 1.08 [7,88,90]. Interestingly, one of these studies investigated associations between L_den_Max and L_den_Min and found stronger associations with road L_den_Min (HR: 1.06; 95 % CI: 1.05; 1.07) compared to L_den_Max (HR: 1.03; 95 % CI: 1.02; 1.03), suggesting that effects of noise on sleep are especially relevant concerning overall cancer mortality [7].

In conclusion, much more research is needed to elucidate whether transportation noise is a risk factor for cancer. So far, focus has been on only a few cancer types, mainly breast and colon cancer. However, transportation noise may also increase the risk of other cancer types, given the suggested mechanisms behind noise-associated pathologies (section 2).

#### 1.3.9 Hearing loss and tinnitus incidence

Noise exposure can affect hearing through increased ROS that have effects on outer hair cells of the cochlea, especially in the 3–6 kHz region, resulting in sensorineural noise-induced hearing loss (NIHL) [[146], [147], [148], [149], [150], [151]]. The risk of NIHL increases if noise exposure exceeds the equivalent A-weighted sound pressure level (LA_eq_) of 85 dB(A) as repeated exposures for an extended period (Fig. 1) [147]. This is frequently seen following high occupational noise exposure and recreational noise [147,152]. Furthermore, there is a high risk of NIHL with frequent exposure to transient impulse-like sounds, such as shooting and explosions from military activities [151]. These sudden and transient sound exposures can be > 140 dB SPL and result in blast injuries of the sense of hearing immediately [153].

It is well-known that loud sound exposures above 85 dB(A) can result in temporary threshold shifts, where the hearing thresholds return to the pre-exposure threshold levels after some time [154]. Recurrent sound exposures can lead to permanent threshold shifts with permanent damage of the outer hair cells in the cochlea [155]. Rodent experiments have shown that sound exposure resulting in a temporary threshold shifts can lead to synaptopathy (damaged synapses between inner hair cells of the cochlea and the spiral ganglion neuron) [156,157]. This is referred to as hidden hearing loss because synaptopathy occurs even though the cochlea's outer hair cells are not damaged and, thereby, do not affect hearing thresholds [157,158].

Tinnitus is perceived by the affected individual as a phantom sensation of noise. There is a high risk of tinnitus in patients with NIHL and other types of hearing loss [[159], [160], [161]]. It has also been suggested that tinnitus can result from spiral ganglion neuron fiber loss due to synaptopathy [158,162].

While noise exposure at levels above 85 dB(A) can harm hearing and lead to tinnitus, much less is known about whether exposures below that level can affect the auditory system, such as transportation noise. The general understanding is that noise exposure below 80 dB(A) cannot harm hearing. However, a recent nationwide study from Denmark found that exposure to road traffic noise was associated with higher risk of tinnitus with adjusted HR of 1.06 (95 % CI: 1.04; 1.08) and 1.02 (95 % CI: 1.01; 1.03) per 10-dB increase in 10-year exposure to L_den_Min and L_den_Max, respectively [11]. The highest HRs were found among people without hearing loss and among those who had never been in a blue-collar job. This demonstrates that the cause leading to tinnitus may differ fundamentally from the well-known associations between hearing loss in general, particularly NIHL and tinnitus related to distress. Transportation noise is a known stressor and stress can increase the loudness of tinnitus and the distress caused by the condition [163]. Tinnitus symptoms are likely enhanced in stressful periods, where stress hormones can affect the limbic, reticular, and auditory systems, as negative thoughts towards tinnitus affect the ability to habituate to the symptoms [164,165]. Cantuaria et al. demonstrated the highest HRs for L_den_Min, a potential proxy for nighttime noise exposure [11,166]. Stress and tinnitus may form a vicious circle as sleep deprivation increases stress, which has negative impact on tinnitus [167]. Tinnitus can itself affect sleep initiation and the resumption of sleep if awakening occurs during night [168]. Of note, also the indirect noise pathway can induce auditory effects.

The mechanism regarding how environmental noise affects the auditory system is not well understood and requires further research. It is, however, unlikely that the mechanism is identical to the tinnitus associated with hearing loss in general.

#### 1.3.10 All-cause mortality

With increasing evidence that transportation noise has a systemic impact on the body and may thus affect additional fatal outcomes beyond CVD, several cohort studies on all-cause mortality have recently been published using long-term exposure assessment based on established prediction models and accounting for most relevant confounding factors, such as age, sex and socioeconomic variables (Table 2) [7,77,90,93,95,97].

**Table 2:** Characteristics of the identified original studies investigating the effect of transportation noise on all natural cause mortality.

Paper	Cohort^a^ (Country)^b^	Cause	Study population			Noise source	Exposure characterization	Adjustment for air pollution	Exposure metric	Relative risk (95 % confidence interval)^c^
			N	Sex/Age	Follow-up					
Grady (2023) [97]	NHS, NHSII (USA)	All natural cause	117,364	Female/mean 57.3 years	1994–2014	Aircraft	Aviation Enviro Design Tool	PM_2.5_	L_dn_^d^	Aircraft: 1.03 (0.94–1.12)
Sørensen (2023) [7]	DNC (DK)	All natural cause	2.6 million	Both/>50 years	2000–2017	Road	Nordic Prediction Method	PM_2.5_	L_den_	Road: 1.091 (1.087–1.095) Rail: 0.997 (0.964–1.032)^e^
						Rail				
Vienneau (2023) [6]	SNC (CH)	All natural cause	4.2 million	Both/>30 years	2000–2014	Road	SonBASE	PM_2.5_	L_den_	Road: 1.045 (1.041–1.050) All sources: 1.044 (1.039–1.048)
Hao (2022) [77]	UK Biobank	All-cause	342,566	Both/40–69 years	2006 (+app.. 9y)	Road	CNOSSOS-EU	–	L_Aeq,24h_	Road: 1.08 (1.04–1.12)
Klompmaker (2021) [169]	Dutch National Cohort (NL)	All-natural cause	10.5 million	Both/>30 years	2013–2018	Road	STAMINA	PM_2.5_ (road only)	L_den_	Road: 1.002 (0.999–1.006) per 7.5 dB^§^ Rail: 1.004 (1.001–1.007) per 9.4 dB^§^
Thacher (2020) [90]	DDCH (DK)	All natural cause	52,758	Both/50–64 years	1993–2016	Road	SoundPLAN	PM_2.5_	L_den_	Road: 1.08 (1.05–1.11) per 10.4 dB
Andersson (2020) [95]	PPS (SE)	All natural cause	6304	Male/47-45 years	1975–2011	Road	Nordic Prediction Method	NO_X_	L_Aeq,24h_	Road: 0.986 (0.906, 1.073)^f^

Abbreviations: N = Number of participants.

a DDCH = Danish Diet, Cancer and Health cohort, DNC = Danish Nurse Cohort, NHS/NHSII = Nurses' Health Study, PPS = Primary Prevention Study, SNC = Swiss National Cohort.

b CH = Switzerland, DK = Denmark, SE = Sweden, UK = United Kingdom, USA = United States of America.

c If not otherwise indicated, relative risks refers to a 10 dB increase related to the maximum façade value.

d The L_dn_ is the average equivalent sound level over a 24 h period, with a penalty added for noise during the nighttime hours of 22:00 to 07:00.

e The relative risk has been converted to per 10 dB (based on reported effect size per increment in original study).

f Derived from categorical analysis by means of a random effects meta-regression. Effect estimates per categories were weighted according to the inverse variance of the effect estimates and the weight of the reference category was estimated from the distribution of the sample size across all noise categories.

**Fig. 7:**
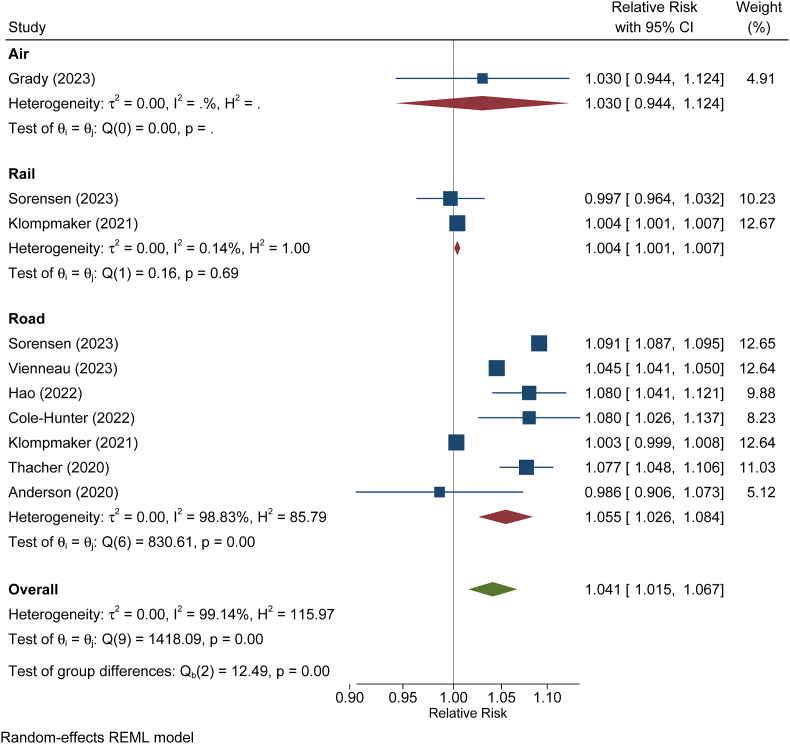
Meta-analysis of cohort studies on all-cause mortality in relation to transportation noise, stratified by source. Relative risks refer to a 10 dB increase in L_den_. Graph was created *de novo* from data of the indicated studies (also cited in the text).

Seven studies addressed associations with road traffic noise (Fig. 7). Thereof, four studies reported significant associations ranging between 4.5 and 8 % increase in mortality per 10 dB increase in road traffic noise and one study reported a significant association with railway noise [169]. One smaller study from Sweden did not observe any association with transportation noise. According to a random effect meta-analysis, the five European cohort studies addressing road traffic noise yielded a pooled relative risk of 1.06 (95 % CI. 1.03; 1.08) per 10 dB.

Sørensen et al. reported separate estimates for road and railway noise and observed no significant associations between railway noise and mortality if expressed as risk increase per 10 dB [7]. However, in this study, an increased relative risk for all 5-dB noise exposure categories above an L_den_ of 35 dB was observed compared to the reference category (<35 dB). However, the exposure-response function did follow a continuously increasing pattern and thus linearization of the curve resulted in absence of association. Grady et al. addressed only aircraft noise and did not observe any significant association [97]. In the U.S. study on aircraft noise [97], only 7 % of the population was exposed to >50 dB L_dn_. Consequently, a substantial part of the study population is expected to be exposed to considerably higher levels of road traffic noise than aircraft noise, which thus may have masked the association with aircraft noise. Vienneau et al. provided a relative risk for road traffic as well as for the energetic sum of railway, aircraft and road traffic noise on all-natural cause mortality [93]. The latter relative risk was very similar to the one for road traffic noise.

The lowest effect threshold was presented in some of the papers either by non-parametric splines or by categorical analysis. In terms of L_den_, significant associations were observed in Sørensen et al. above 35 dB for railway noise and above 45 dB for road traffic noise [7], and in Thacher et al. above 55 dB [90]. Vienneau et al. showed non-parametric splines for cardiovascular mortality [6], where associations were observed to become significant above 30 dB (railway), 38 dB (road) and 50 dB (aircraft noise). This indicates that new studies with large sample sizes and high-quality noise exposure modeling are able to demonstrate detrimental effects from noise even below the WHO guideline values. This conclusion is supported by studies on the incidence of other outcomes that also found low effect thresholds such as for IHD [63], stroke [5], heart failure [8] or diabetes [170].

#### 1.3.11 Burden of disease

To transfer scientific knowledge on noise and health to preventive and regulatory measures, it is important to quantify the attributable health impacts on the population. A key quantitative health impact assessment metric is Disability-Adjusted Life Years (DALYs), which includes both morbidity and mortality. The growing use of DALYs is primarily driven by WHO and the Global Burden of Disease (GBD) study [171], as they use this metric when estimating burden of disease (BoD) attributable to several risk factors in addition to a wide range of physical and mental disorder and disabilities.

In 2011, WHO estimated DALYs attributable to transportation noise in Western Europe for the first time [172], using noise exposure data assessed according to the Environmental Noise Directive, 2002/49/EC (END). High noise annoyance, high degree of sleep disturbance, IHD, cognitive impairment, and tinnitus were included as health outcomes in this BoD assessment by WHO. Since then, the European Environmental Agency has estimated environmental noise to be the second most important environmental risk factor, after air pollution, in driving disease burden in the EU [173]. Noise was associated with 22 million DALYs due to high annoyance, 6.5 million DALYs due to high sleep disturbance, 48,000 DALYs due to IHD, and 12,000 premature deaths (due to IHD) per year.

As described above, the knowledge in the field of noise and health has grown rapidly since the WHO systematic review was published in 2018 [2]. Updated knowledge of the causal association between noise and various health outcomes from high-quality studies is an important pillar in health impact assessment. Only a limited number of studies have estimated the disease burden due to environmental noise [[174], [175], [176], [177], [178], [179], [180]], and these studies often differ in methodological aspects, which makes comparison across areas and studies difficult. To estimate DALYs, several input parameters are required. In addition to selecting health outcomes with associated exposure-response functions, noise exposure distribution and health data are needed for the population for which the calculations will be performed.

A recent BoD study in the Nordic countries, Denmark, Finland, Norway, and Sweden, aimed at using a harmonized approach and comparable input data to estimate DALYs attributable to road traffic and railway noise [179]. This study also addressed the influence of methodological choices in the estimation of BoD. Noise exposure assessment according to END was used as the primary source of exposure. In addition, nationwide noise models were available for Denmark and Norway. Transportation noise contributed with a considerable disease burden in the Nordic capitals, between 300 and 500 DALYs/100,000 for road traffic noise and 40–150 DALYs/100,000 for railway noise. The estimated BoD attributable to road traffic noise was found to be in the same order of magnitude as for PM_2.5_ air pollution, as reported by GBD. Furthermore, the DALY estimates for road traffic noise were increased with up to 17 % when stroke and diabetes were included in addition to the high annoyance (HA), high sleep disturbance (HSD), and IHD. In addition, several important methodological findings were uncovered. First, the assessment based on noise exposure data according to END considerably underestimated the burden due to transportation noise at the national level. The study revealed considerably higher DALY rates attributable to road traffic noise when based on the nationwide models compared to END. Thus, the degree of coverage contributes considerably to the higher estimates for the nationwide models. Secondly, the study revealed different interpretations across the Nordic countries of the geographical areas to be included in the END noise mapping and the noise exposure assessment method. Thus, no comparable DALYs attributable to noise could be assessed for the Nordic countries, only for the capital cities using additional noise exposure data beyond what was reported to the European Commission according to END. Differences in definitions of agglomerations according to END across geographical areas and time have also previously been reported for European countries [181]. Lastly, by using lower cut-offs of L_den_ and L_night_ the DALY rates for HA and HSD increased by up to 40 % compared to the estimates based on the END mapping thresholds (L_den_ 55 dB and L_night_ 50 dB).

Another recent BoD study estimated DALYs from HA, HSD, IHD, stroke, and diabetes attributable to long-term transportation noise exposures in England for the adult population in 2018. It was concluded that ∼97,000 DALYs are lost due to road traffic, ∼13,000 due to railway, and ∼17,000 due to aircraft noise [180].

It is important to further update the scientific evidence and to develop harmonized methods to reliably quantify the BoD of environmental noise. Previous BoD studies have selected a few specific outcomes but none of them have considered new studies on e.g., all-cause mortality as discussed in the previous chapter (1.3.10). Thus, current BoD estimates are expected to substantially underestimate the impact of transportation noise. As transportation noise contributes considerably to the environmental BoD, inclusion of noise as an environmental risk factor in the GBD is strongly encouraged.

## 2 Pathophysiological mechanisms of noise exposure

### 2.1 General pathophysiological mechanisms

**Fig. 8:**
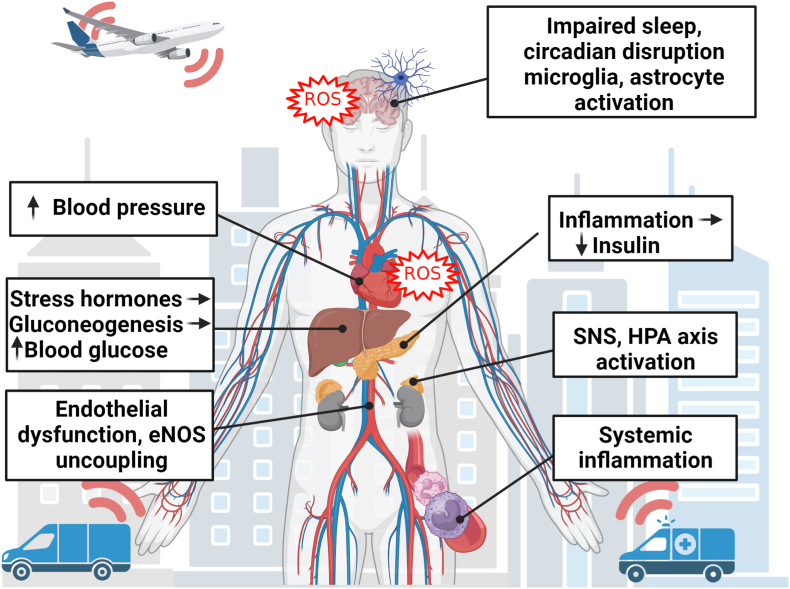
**Pathways activated by noise to trigger adverse health effects.** Noise perception starts in the brain, leading to neuronal activation associated with disruption of circadian rhythms (especially by nighttime noise causing sleep deprivation and fragmentation), neuroinflammation, and cerebral oxidative stress. Noise activates down-stream stress responses such as activation of the sympathetic nervous system (SNS) and the hypothalamic-pituitary-adrenal (HPA) axis, leading to stress hormone release such as catecholamines and cortisol with secondary activation of the renin-angiotensin-aldosterone system. This cascade converges in oxidative stress and inflammation associated with eNOS uncoupling, endothelial dysfunction, high blood pressure, and hyperglycemia, all well-known triggers of cardiovascular sequelae. Modified from Ref. [187] with permission. Copyright © 2021 the authors. The image was created using Biorender.com.

Decades of research investigating the detrimental health outcomes of noise exposure have identified ‘direct’ and ‘indirect’ pathways that contribute. The direct pathway describes the manner in which high-intensity noise produces mechanical damage in the inner ear and the downstream physiological responses to such an exposure (Fig. 8). The indirect pathway, on the other hand, was suggested by Babisch in 2002 [182], and describes how noise exposure at “sub-hazardous” intensities for the cochlea is able to elicit cognitive, emotional, and physiological responses. Both pathways contribute to stress responses, which lead to elevated levels of catecholamine, adrenocorticotropic hormone (ACTH), and cortisol secretion via SNS and HPA activation. Sound is perceived by the auditory cortex and the stress response is thought to be activated in hypothalamus but the exact sequence of signaling events, interplay between different brain regions and identity of activated neurons remains to be fully elucidated and are the subjects of ongoing investigations [183].

Chronic activation of stress systems contributes to peripheral and central adverse health effects [29]. Exposure to loud or unwanted noise can interrupt sleep, cause emotional stress, or disrupt daily activities. Reduced sleep quantity and quality, and chronic stressors can mimic many of the effects that are observed following noise exposure, including decreased melatonin production which result in disruption of the circadian and endocrine systems and an increased allostatic load [184]. Impaired sleep also increases leptin and ghrelin levels as well as appetite, coinciding with reduced insulin sensitivity [184]. Chronic activation of the HPA axis leads to high cortisol levels, which can heighten risk factors for CVDs, including increased blood pressure, vascular reactivity, and anxiety [185]. In concert, sympathetic activation can increase blood pressure as well as proinflammatory and procoagulant responses [186]. Overall, noise triggers neuroinflammation and cerebral oxidative stress, blood pressure increases, endothelial dysfunction, cardiovascular and systemic oxidative stress, inflammation and myelomonocytic infiltration of peripheral tissues, and dysregulation of circadian rhythms (Fig. 8) [13]. Mechanistically, these noise-induced disruptions activate the endothelin-1 (ET-1) pathway and RAAS, leading to vasoconstriction and a rise in circulating inflammatory markers including tumor necrosis factor-alpha (TNFα), interleukins IL-1 and IL-6, and C-reactive protein (CRP), and oxidative stress biomarkers [184]. In addition, ET-1 and RAAS activation contribute to medial thickening, structural remodeling and hypertrophy (as mentioned in section 1.2) promoting the onset of heart failure by noise exposure [37,38].

The original concept of “oxidative stress” was formulated in 1985 as “*imbalance of prooxidants and antioxidants in favor of the prooxidants*” [188]. In subsequent years, progress in redox research on the role of oxidants in redox signaling and redox regulation called for an update of the concept [189], which led to the updated definition of oxidative stress as “*an imbalance between oxidants and antioxidants in favor of the oxidants, leading to a disruption of redox signaling and control and/or molecular damage*” (reviewed in Ref. [190]). In order to account for the beneficial versus detrimental nature of oxidative stress, different subforms of oxidative stress were classified, ranging from physiological oxidative stress (eustress) to excessive and toxic oxidative burden (distress) [[190], [191], [192]]. Hydrogen peroxide is a central redox signaling agent in physiological oxidative stress (eustress) [193]. In the context of noise exposure, one could ask whether a physiological low level of noise is required, especially when in view of a comfortable social environment, to contribute to health-promoting eustress, such as the documented positive psychosocial effects of music. In contrast, high levels of (annoying) noise initiate detrimental distress. Whether prolonged absolute silence is beneficial or harmful needs to be examined.

**Fig. 9:**
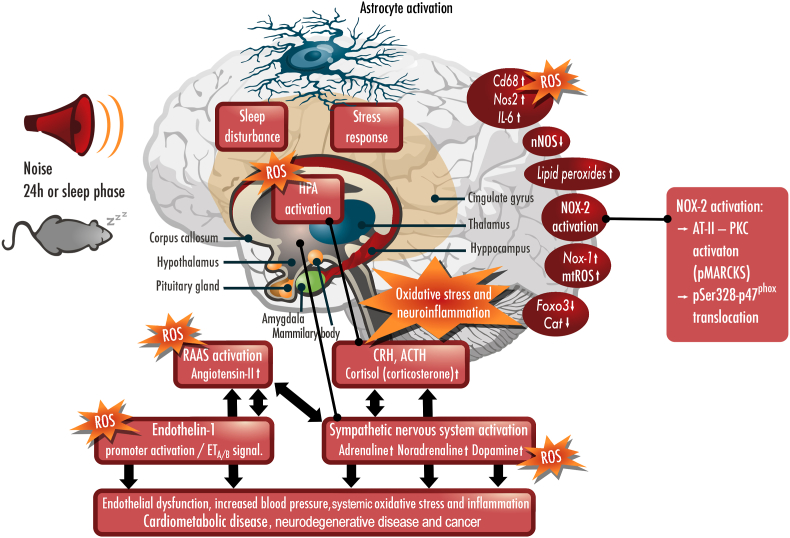
**Noise exposure induces neuronal activation with multiple targets for oxidative stress.** First-line neuronal events in response to noise exposure are sleep disturbance (when exposed during the sleep phase) and stress response reactions linked with activation of the hypothalamic-pituitary-adrenal (HPA) axis and the sympathetic nervous system. This leads to the release of stress hormones (glucocorticoids and catecholamines) and secondary activation of the cerebral (and systemic) renin-angiotensin-aldosterone system (RAAS) as well as endothelin-1 expression. These potent triggers of inflammation and oxidative stress will lead to activation of NOX-2 via protein kinase C (PKC) and p47^phox^ phosphorylation, expression of inflammation markers and increased lipid peroxidation in the brain. Moreover, noise caused down-regulation of neuronal nitric oxide synthase (nNOS), and loss of antioxidant genes such as catalase (*Cat*) and forkhead box O3 (*Foxo3*) transcription factor. All these changes induce a neuroinflammatory phenotype with cerebral oxidative stress. These stress hormones and vasoconstrictors lead to similar adverse changes in the cardiovascular (and pulmonary) system, which may promote the development of manifest diseases, including cardiometabolic disease, dementia, and cancer. The HPA axis, sympathetic nervous system, RAAS, ET-1 expression, and neuroinflammation are redox-regulated and vice versa can induce oxidative stress via NOX-2 activation and other sources. CRH, corticotrophin-releasing hormone; ACTH, adrenocorticotrophic hormone; CVD, cardiovascular disease. Reused from Ref. [45] with permission.

Oxidative stress from any source, including NOX2-derived or mitochondrial H_2_O_2_/O_2_^•−^ [194], can trigger ET-1 expression [195,196], which can then lead to a vicious circle that contributes significantly to the cardiovascular oxidative stress and damage [197]. In hypertensive rats, plasma angiotensin II (one product of RAAS) and ET-1 levels are positively correlated with blood pressure [198], and both are decreased following treatment with bosentan (ET_A/B_ receptor blocker), implying crosstalk between the two systems [199]. The connection between these two pathways has a potent physiological influence and triggering either pathway can produce a strong vasoconstrictive stimulus. Bulk RNA sequencing of heart, kidney, and aorta in a translational murine model of noise exposure indicated downregulation of antioxidant enzymes (superoxide dismutase 1, glutathione peroxidase 1) as well as the transcription factor Forkhead box protein O (FOXO). This implies that noise activates systems leading to oxidative stress (i.e., activation of inflammatory myeloid cells) at the same time as downregulating expression of the antioxidant enzymes [14,16]. Fig. 9 summarizes some of the oxidative stress-driven pathomechanisms linked to noise exposure that results in an increased susceptibility to various diseases [29].

Understanding the crosstalk between the stress response, oxidative stress and vasoconstrictor mechanisms has been vital in understanding how noise elicits detrimental health effects. Pre-clinical models using approaches that directly and indirectly study stress as a key component support the association [13,22,67,200]. Critically, glutamatergic signaling in the amygdala of rats [201] and heightened amygdala activity in humans [47,[202], [203], [204]], are indicative of stress-induced arousal, and appear to be enhanced following noise exposure. Corticosterone is increased in the plasma of noise-exposed rats and mice [29], implying activation of the HPA axis. Increases in plasma and kidney adrenaline and noradrenaline indicate sympathetic activation in noise-exposed mice [14] and rats [205,206]. Activation of these stress response systems corresponds with detrimental cardiovascular readouts in murine models of noise exposure, including hypertension [207,208], increased myocardial fibrosis [209], and atrial interstitial fibrosis [210]. Of note, enhanced stress hormone signaling has also been associated with a higher risk of cancer [211], and increased amygdala metabolic activity was reported to correlate with adiposity [202,212] and diabetes [213], in relation to noise exposure. The clinical correlates of this noise-induced stress response are summarized in Textbox 2. The subsequent sections shed light on the downstream molecular mechanisms.

Textbox 2 **Clinical correlates of noise-induced stress response.**

In line with an activation of the HPA axis, early studies identified elevated cortisol levels in humans exposed to transportation noise or intermittent pink noise^2^ [214,215]. These results were extended to noise-exposed children with findings of chronically elevated free cortisol in the first half of the night and serious disturbances of the circadian rhythm of cortisol concentrations [216]. Two clinical trials reported elevated morning cortisol levels in women living near airports [217] and higher evening cortisol concentrations in participants exposed to aircraft noise [218]. In subsequent studies, it was pointed out that noise-induced increases in cortisol levels may be associated with the degree of annoyance by noise [219] and autonomic arousals independent of sleep impairment [220]. In parallel to cortisol and following SNS activation, adrenaline, and noradrenaline levels were elevated by acute and chronic noise exposure [221]. A subsequent study indicated that nighttime noise is the driving force for elevated noradrenaline levels [222]. Acute nighttime noise also increased adrenaline associated with endothelial dysfunction [30] and has been associated with stress-induced cardiomyopathy (also known as Takotsubo syndrome) [223].

Alt-text: Textbox 2

#### 2.1.1 Noise-dependent adverse effects on the cardiovascular system

**Fig. 10:**
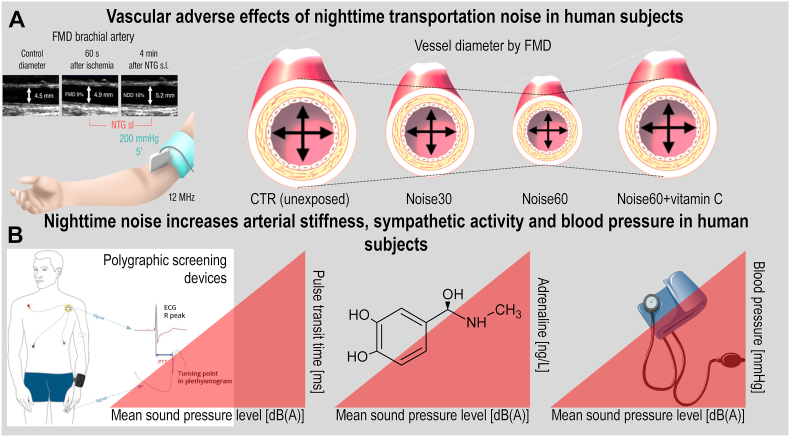
**Noise-induced health effects in human interventional field studies.** (**A**) *Left panel:* Method used to determine endothelial function via the flow-mediated dilation (FMD) technique. Following the baseline brachial artery diameter measurement, a blood pressure cuff is inflated for 5 min to supra-systolic blood pressure to stop forearm blood circulation. The release of the cuff after 5 min causes a strong reactive hyperemia and the increased forearm blood flow. Thus, shear stress on endothelial cells results in an endothelium-dependent vasodilation that is mainly dependent on the release of the endothelium-derived nitric oxide. FMD is measured by high-resolution B-mode ultrasound. Adapted from Ref. [241] with permission. *Right panel:* Schematic presentation of adverse effects of simulated nighttime aircraft or train noise on FMD of the brachial artery in response to postischemic hyperemia and the beneficial acute effects of the antioxidant vitamin C [30,234]. (**B**) Schematic picture of polygraphy screening devices (electrocardiogram (ECG) and sensor for fingertip plethysmography or tonometry; image a curtesy of SOMNOmedics GmbH, Randersacker, Germany). SNS activation and arterial stiffness are assessed by decreased pulse transit time, increased circulating adrenaline levels, and blood pressure increase [30,237]. The entire scheme was arranged for a review article [13] and reused with permission.

Investigation of the cardiovascular health effects of noise in humans dates back to the 1960s. An early study revealed that noise exposure led to the narrowing of peripheral blood vessels in individuals engaged in exercise [224]**.** Another study claimed that exposure to noise or music elicited variable cardiac output and minute flow and concluded that it was the stimulus' intensity rather than the nature of the sound caused the responses [225]. A study supporting this conclusion included 1005 German industrial workers, showing that workers in noisy industries were more likely to have problems of the peripheral circulation and heart as well as disturbed balance [226]. These studies describe the ‘direct’ pathway of the noise reaction scheme, but only account for noise exposure during waking hours. Also, factory workers exposed to high noise levels (L_Aeq_ > 80 dB(A)) were found to have significantly higher glutathione peroxidase levels, systolic and diastolic blood pressure, and DNA damage than office workers (L_Aeq_ 40–50 dB(A)) [227]. Exposure to one night of transportation noise in humans was sufficient to increase blood pressure the following day [228,229]. This is likely due to interference with blood pressure dipping by repeated nighttime autonomic arousal [230]. Another human field study found that one night of aircraft noise exposure (L_eq_ 46.3 dB^(A)^, peak level 60 dB^(A)^) reduced sleep quality, increased stress hormone levels, caused endothelial dysfunction, and decreased pulse transit time (reflecting SNS activation) in healthy individuals (Fig. 10) [30]. Notably, when exposed to noise while awake, feelings of “annoyance” appear to be linked to conditions such as anxiety and depression [231,232] as well as atrial fibrillation [24,233].

In two additional small human field studies, vitamin C was shown to alleviate endothelial dysfunction associated with one night of aircraft noise exposure or railway noise (Fig. 10) [30,234], suggesting that oxidative stress plays a key role in the underlying pathophysiology [235]. Healthy individuals subjected to either 30 or 60 train noise events during the night (average SPL of 52 and 54 dB(A)) resulted in reduced sleep quality and impaired flow-mediated dilatation (FMD) of the brachial artery compared to control individuals exposed to background noise (average SPL 33 dB(A)) [234]. Furthermore, the plasma proteome of these subjects appeared to shift toward a pro-thrombotic and pro-inflammatory state. Additionally, the SAPALDIA consortium reported that chronic exposure to nocturnal intermittent train or road traffic noise correlated with arterial stiffness, measured as pulse wave velocity [236]. These investigations and others underline the importance of sleep disruption as a cardiovascular risk factor. Importantly, these studies also indicated that noise can impact health even when subjects are apparently unaware. These human studies, pointing to the involvement of inflammation, oxidative stress or adverse redox signaling in noise-related CVD, are further supported by mechanistic animal studies described in the preceding sections, building upon previous reviews [13,22,67,200].

Given the high incidence of CVD and the fact that IHD was the first disease directly linked to noise exposure, it is important to investigate how noise can affect the risk and severity of CVDs. It should also be kept in mind that epidemiological studies have linked noise exposure with a higher risk of other major diseases, including diabetes, cancer, and dementia (see section 1). One of the few human studies investigating the impact of noise on CVD-associated pathways reported that one night of aircraft noise was enough to increase serum levels of 3-nitrotyrosine-modified proteins in patients with established coronary artery disease [16,237]. This is strong evidence for an increased oxidative stress. Endothelial dysfunction was also pronounced in these patients, suggesting that a compromised endothelium or pre-activated oxidative milieu could predispose to the harmful effects of noise. The exposure of healthy volunteers to one-night of noise (L_eq_ 45 dB(A)) also impaired diastolic heart function, as assessed through sequential echocardiography, compared with a control group (L_eq_ 37 dB(A)) [238]. Importantly, endothelial dysfunction is also an early marker of diabetes [239] and this relationship could partially explain the link between higher amygdala activity and the cardiometabolic effects of transportation noise [240].

**Fig. 11:**
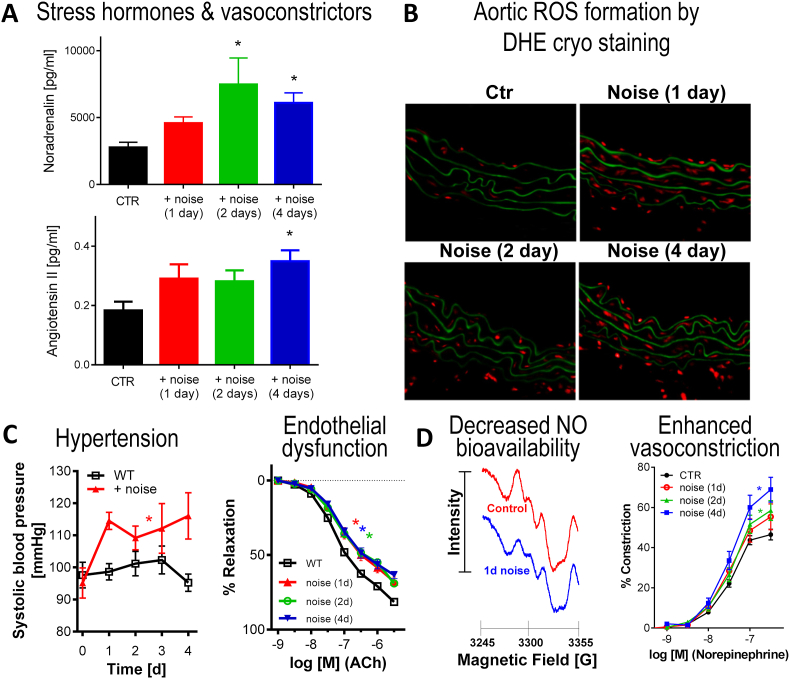
**Summary of vascular effects of noise exposure in healthy mice.** Noise increases serum levels of the catecholamine noradrenaline (=norepinephrine) and the vasoconstrictor angiotensin-II determined by ELISA (**A**) as well as vascular oxidative stress in the aortic wall of mice measured by dihydroethidium (DHE) staining (**B**). Consequently, noise increases systolic blood pressure determined by the tail cuff method and impairs endothelial function assessed by the isometric tension method using acetylcholine-dependent vasodilation (**C**). Bioavailability of the important vasodilator nitric oxide (^•^NO) was decreased in the aorta of noise-exposed mice (measured by electron spin resonance spectroscopy), whereas sensitivity to vasoconstrictors such as noradrenaline was increased (determined by isometric tension method) (**D**). Data are mean ± SEM, at least n = 6 mice/group. *, p < 0.05 versus unexposed CTR. WT, wildtype; CTR, control. Adopted from Ref. [14] with permission.

High-intensity industrial noise during longer periods was also found to induce hypertension in rhesus monkeys [207] and in rats [242]. Furthermore, rats exposed to very high levels of white noise (100 dB(A)) had impaired endothelium-dependent relaxation of the thoracic aorta, higher sensitivity to the vasoconstrictor agonist serotonin, and increased systolic blood pressure [243,244]. The latter studies used very high SPL levels that could incur physical damage, however, studies of “sub-hazardous” levels of noise exposure (<80 dB(A)) are rare. More recent studies investigated the effects of aircraft noise on cardiovascular biomarkers by exposing mice to around the clock lower SPL (e.g., aircraft noise with a L_eq_ of 72 dB(A) and peak level of 85 dB(A) for 24 h for 1, 2 and 4 days) [14,16]. These identified a significant noise-induced increase in stress hormone levels, blood pressure, and vascular and cerebral oxidative stress, all associated with impaired endothelial function and diminished vascular nitric oxide levels (Fig. 11). While the protocol of noise exposure in experiments with animals has differed quite markedly between studies, the physiological consequences have been consistent and comparable to the results reported in humans.

In animal studies, RNA-sequencing has revealed noise-induced dysregulation of gene networks associated with endothelial and vascular signaling and even potential risk marker genes [14]. Importantly, mice exposed to white noise (similar exposure time and mean SPL as to aircraft noise) did not show these cardiovascular effects, implying that noise characteristics (such as frequency or pattern) rather than the SPL determine the extent of cardiovascular damage [14]. Since white noise represents a continuous “swoosh” including a broad range of frequencies, it may be even the pattern of aircraft noise based on the intermittent irregular crescendo and decrescendo sound levels of the starting and landing events. Aircraft noise exposure during sleep was substantially more detrimental to the cardiovascular system than exposure during the awake phase, and cardiovascular damage was almost entirely prevented by *Nox2* deletion, pointing to the crucial role of inflammatory cells in mediating noise-induced cardiovascular effects [16]. In mice, endothelial dysfunction and blood pressure increases were established very rapidly i.e., on the first day of noise exposure, and persisted over 4 weeks of continuous noise exposure, indicating no apparent adaptation [245]. However, when noise exposure ceased, vascular dysfunction and oxidative stress in conductance vessels returned to normal within 4 days, whereas the damage to microvessels of the brain, envisaged by ROS formation and impaired relaxation persisted [246].

Importantly, noise exacerbated blood pressure increases and endothelial dysfunction in mice with pre-existing hypertension (induced by angiotensin-II infusion) [247] and aggravated cardiovascular damage in three models of diabetes (unpublished data, Mihalikova et al.). Moreover, exposure to aircraft noise for 4 days primed the cardiovascular system in favor of an inflammatory phenotype with enhanced H_2_O_2_/O_2_^•−^ formation and infiltration of pro-inflammatory immune cells. The latter resulted in exacerbated damage of the heart and impaired cardiac function in mice subjected to MI by ligation of the left anterior descending artery [248]. Exposure of animals to noise prior to MI also increased cardiac mitochondrial O_2_^•−^ formation, impaired mitochondrial respiration and increased pro-inflammatory cytokines in the heart. In addition, noise pre-exposure also caused endothelial dysfunction, and more pronounced increases in vascular ROS levels. This correlates well with observations from the population-based Gutenberg Health Cohort Study as subjects with incident MI at follow up revealed elevated CRP at baseline and worse left ventricular ejection fraction (LVEF) when they had a history of high noise exposure and subsequent annoyance at enrolment [248]. The most studied effects of noise on the development of CVD and death due to CVD in humans are summarized in sections 1.3.1, 1.3.2, 1.3.3, 1.3.4. For more mechanistic insight and human studies on adverse cardiovascular effects of noise please refer to Refs. [13,22,200].

#### 2.1.2 Noise-dependent adverse effects on the brain

**Fig. 12:**
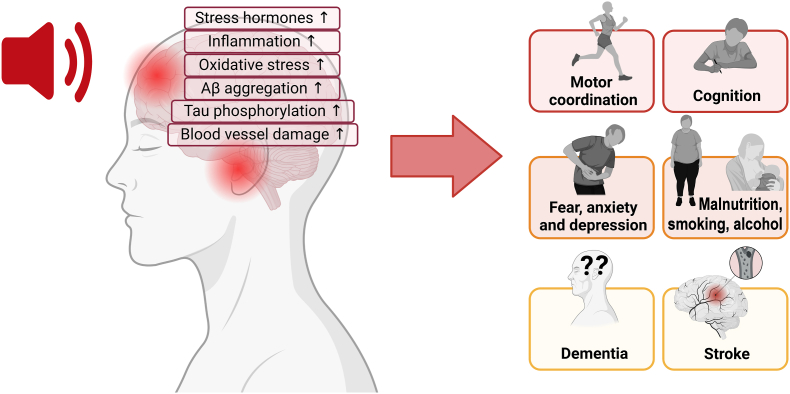
**View of the brain–body interaction in response to noise stress**. Activation of the HPA axis and the sympathetic system triggers the release of different stress hormones (dopamine, noradrenaline, adrenaline, and cortisol), leading to oxidative stress and inflammation and the modulation of behavioral and neuronal processes, (e.g., physical exercise [264] or smoking) [135]. Tau phosphorylation and Aβ accumulation by noise promote dementia and stroke. Modified from Ref. [125] with permission and created with BioRender.com.

Noise can elicit a variety of responses that ultimately culminate in neuropsychiatric disorders (Fig. 12), including neuroanatomical changes [249,250]. In animal studies, very high-intensity noise pulses (≈200 dB) were found to increase the expression of the proto-oncogenes c-Fos and c-Myc. This happened in the cortex, thalamus, and hippocampus as rapidly as 2 h after exposure. While c-Myc levels returned to control levels after seven days, c-Fos remained elevated for at least 21 days. Additionally, β-amyloid precursor protein (APP) levels increased, creating a phenotype indicative of human traumatic brain injury and Alzheimer's disease [251]. While this study provided early evidence of noise-provoked damage in the brain, the intensity of noise was very high and not representative of average noise exposure in everyday life. Other studies using lower-intensity noise have, however, generally supported that noise produces damage within the brain [125]. Cheng et al. used a murine noise exposure model of 80 dB SPL, 2 h/day for 1–3 weeks, and found that noise could cause structural and functional changes in the auditory cortex and hippocampus [252]. They additionally suggested that while the auditory cortex was affected by a realistic level of noise, it appeared that the hippocampus (a non-auditory brain structure) was more vulnerable, meaning that there are aspects to how noise ‘propagates’ within the brain that are poorly understood. It is becoming evident, however, that nonauditory symptoms do arise, and noise-induced stress has been found to impair cognition and motor coordination and to cause changes in feeding behavior, fear, and anxiety, possibly arising due to metabolic and anatomical changes in neurons [253,254]. Feeding behavior seems to be particularly susceptible to change following stress. Indeed, humans [[255], [256], [257]] and animals [[258], [259], [260]] prefer more pleasurable food following exposure to stress, and noise is reported to impair eating and lactation behavior [255,261]. Chronic stress may elicit depressive disorders, and recent epidemiological studies have indicated that transportation noise may be associated with depression and other mental disorders [127,262,263], which was also supported by preclinical mechanistic data [249].

It has previously been mentioned that β-APP levels increased because of high-intensity noise exposure [251], indicating a potential link between noise exposure, stress, and neurodegenerative disease. There are standard mechanistic links between these diseases. Alzheimer's disease and dementia are both exacerbated by chronic inflammation and oxidative stress [265], likely through activation of protein kinase C (PKC) and protein kinase A [115], which can then hyperphosphorylate tau and lead to the aggregation of amyloid plaques [266]. APP, the protein from which amyloid beta (Aβ) is cleaved, is a transmembrane protein with a cholesterol-binding domain and is sensitive to oxidation by ROS [267], leading to alterations in membrane fluidity and lipid composition [268], and consequently, the growth of insoluble amyloid plaques that prevent or disrupt neuronal signaling and pruning [269].

There is an essential overlap in pathophysiological mechanisms between Alzheimer's and dementia with those of noise-elicited stress. Oxidative imbalance is a hallmark feature of noise exposure models in both humans and rodents, though neuroinflammation and oxidative stress have only been recorded in the brains of rodents [16]. Wistar rats subjected to 4 weeks of white noise (100 dB(A)) accumulated Aβ_40_ and Aβ_42_ in the hippocampus, which persisted for up to two weeks after noise cessation. These rats also manifested persistent elevations in glial fibrillary acidic protein (GFAP) staining, which indicates astrocyte activation, as well as increases in TNFα and the receptor for advanced glycation end (RAGE) products, indicating that both inflammatory and oxidative processes were likely taking place in the brain of exposed rats [270]. Thirty days of noise exposure has also been reported to cause tau phosphorylation in the hippocampus [271] and increased CRH. These studies are meaningful proofs-of-concept that noise can interact with critical pathways for the pathogenesis of Alzheimer's disease. It should also be noted that these experiments typically studied quite young animals (∼8 weeks). The ability to clear amyloid plaques is reduced with age [272], suggesting that a more severe phenotype could be observed in older populations.

Associations between transportation noise and the risk of Alzheimer's disease have only been investigated in a few studies (e.g., Ref. [132]), suggesting transportation noise as a significant risk factor for neurodegenerative diseases (described in detail in section 1.3.7.). There are also studies indicating that noise exposure can impair cognition [273]. This is likely to occur through the stress hormone-dependent mechanism previously described, as plasma corticosterone was significantly increased in rats following 1, 15 or 30 days of 4-h of 100 dB noise exposure [274]. The latter effects were coincident with increases in superoxide dismutase expression and lipid peroxidation. These rats also had changes in dendritic spines count in the hippocampus and prefrontal cortex and deficiencies in their working and reference memory [275,276]. Other studies have produced similar results by demonstrating a reduction in dendritic processes in the hippocampus of noise-exposed rodents, leading to impairment in memory as well as oxidative stress [270,277]. Increased dopamine levels in the brain following noise stress [[278], [279], [280]] also point to an oxidative influence in these symptoms, as dopamine can be metabolized by monoamine oxidase (MAO) to generate H_2_O_2_. Hydrogen peroxide can activate further ROS sources, which perpetuates the production of other oxidative species and leads to ROS-mediated changes in the morphology of cerebellar Purkinje cells [280]. A neuroinflammatory phenotype involving astrocyte and microglial activation and subsequent oxidative stress was reported in mice following moderate-intensity noise exposure for four days (Fig. 9) [281]. These symptoms were more severe in mice with pre-existing hypertension and primarily associated with noise exposure during sleep [16,247]. *Nox2* knockout mice were protected from these effects, underlining the important role of ROS (H_2_O_2_/O_2_^•−^) and phagocyte dysregulation in perpetuating the damage in the brain following noise exposure [16,187]. Accordingly, it is not surprising that noise has been associated with a higher risk of stroke, especially ischemic stroke (see section 1.3.3). Since stroke is a vascular disease, the same pathophysiological mechanisms are active as described for vascular/endothelial damage in the preceding section, with a central role of inflammation and oxidative stress, both key determinants of stroke development and pathophysiology [282,283].

Concerning the permeability of the blood-brain barrier, there is some mechanistic evidence indicating that noise exposure results in its disruption, and substantial peripheral immune infiltration in the brains of noise exposed mice was recently observed [281]. For example, the exposure of pigs to low-frequency but high-intensity noise (140 dB(A)) increased permeability of the blood-brain barrier due to leaky tight junctions [284]. The most important human correlates for noise effects on the brain are reported in Textbox 3, and more mechanistic insights and human studies on neuropsychiatric effects of noise were summarized previously [125,285].

Textbox 3 **Clinical correlates of noise-dependent adverse effects on the brain.**

Noise has been associated with cognitive impairment and memory deficits in children and adults. However, our understanding of the harmful effects of noise on manifest diseases in the brain remains limited due to a lack of high-quality studies [133,286,287]. Similarly, although a few epidemiological studies have indicated that transportation noise may be a risk factor for depressive and anxiety disorders, more research is needed to establish the association [231,288,289]. Interestingly, a prospective study covering entire Denmark found both road traffic and railway noise to be associated with a higher risk of dementia, especially Alzheimer's disease [132], similar to results obtained in a small US study [133]. Several studies have found that transportation noise is associated with a higher risk of stroke [4,290]. In addition, human studies reported an association of chronic noise exposure with overweight [119,120,291], which may in part be attributed to different feeding behavior by dysregulated neuronal signaling.

Alt-text: Textbox 3

#### 2.1.3 Impaired sleep

Another significant health impact of nighttime noise is sleep disturbance [200,292]. It is well established that insufficient sleep profoundly impacts upon mental health [293,294]. Furthermore, sleep fragmentation and deprivation in general, and in response to nocturnal noise, are established cardiovascular risk factors [295,296]. Several studies have found nighttime aircraft noise to be associated with hypertension in people living near airports [228,297,298], other studies, however, failed to confirm this [299]. The underlying pathomechanisms may be related to circadian dysregulation of metabolic, endocrine [300], and immune pathways [301]. Sleep restriction [302] and fragmentation [303] also induce endothelial dysfunction and potentiate cerebral oxidative stress [304], likely due to increased NADPH oxidase (mainly NOX-2) activation. Similarly, chronic aircraft noise has also been associated with learning and memory impairment in children [286], possibly due to inappropriate activation of NOX-2 [305]. There is also translational evidence that this enzyme is a critical component of noise-induced adverse cerebral and cardiovascular complications; mice with a genetic deletion of NOX-2 (gp91phox-) were almost completely protected from noise [16]. NOX-5 could be another candidate for noise-induced ROS formation in humans but was so far not studied.

The overlap in the pathophysiological mechanisms of noise and impaired sleep is supported by several human studies. For example, a field study of 75 healthy adults subjected to overnight aircraft noise demonstrated that noise impaired sleep quality, increased adrenaline, and subsequently worsened endothelial function - as determined by FMD [30]. Further, these effects were noise exposure-dependent, clearly linked to the “indirect pathway”. Human studies have been supplemented by translational work, including a study in mice by Carreras et al. that demonstrated endothelial dysfunction and arterial hypertension following 20 weeks of sleep deprivation/fragmentation [303]. Furthermore, the vascular walls showed structural alterations, with disturbed elastic fiber arrangement and aggregated foam cells and macrophages, and the sleep-deprived mice expressed lower levels of mRNA encoding the senescence markers telomerase reverse transcriptase (TERT) and cyclin A, the tumor suppressor p16^INK4^ as well as higher levels of IL-6 [303]. In other animal studies, sleep fragmentation was linked with insulin resistance, NADPH oxidase activation [306], and increased oxidative stress [307], which mirror pathomechanistic elements of noise exposure. Similarly, links between sleep deprivation, increased oxidative stress, manic-like behavior, and memory impairment have also been made in mice [[308], [309], [310]], all triggered by HPA and SNS activation. Sleep was also shown to protect against atherosclerosis [311].

Overall, there is a remarkable overlap in symptoms and readouts of oxidative homeostasis and inflammatory activation between sleep fragmentation/deprivation and noise exposure, as highlighted by mechanistic mouse studies of noise-induced cerebral and cardiovascular damage (reviewed in Refs. [13,22]). Significantly, in mice, noise exposure during the sleep phase contributed to the bulk of the cardiovascular and cerebral damage, with only minor contributions from exposure during the waking phase [16]. RNA sequencing of noise-exposed mouse kidney, heart, and aorta homogenates also revealed that FOXO3 signaling could be the molecular crux of circadian disruption following poor sleep quality, as the transcriptional trigger for noise-induced vascular damage via oxidative stress and inflammation. The role of the impaired circadian clock for noise-induced adverse health effects is explained in more detail in section 2.3.3.

Infrasound refers to frequencies below 20 Hz, while low-frequency sound covers 20–200 Hz. These types of sound come from many environmental sources, including machinery like compressors and ventilation systems, as well as traffic noise. Research shows that wind turbines can generate low-frequency noise exceeding 20 dB inside nearby homes [312], and emerging evidence has linked low-frequency sound with health effects. This is important given the future shift towards renewable energy and its implications for population health. Wind turbine noise has been associated with some negative health effects, particularly annoyance and sleep disturbance, in those living close to wind farms [313,314]. The noise level for wind turbines is associated with multiple factors apart from proximity e.g., wind speed (and its variations) and other meteorologic factors (e.g., wet weather, fog). Overall, evidence indicates that the level of audible noise from wind turbines increases annoyance in nearby residents [315,316] with some studies reporting that annoyance to wind turbine noise is higher than that for traffic-related noise [314,317]. Self-reported sleep disturbance also appears to increase with proximity to wind turbines. One study from Canada has shown that the aggregate annoyance from wind turbines is linked with multiple factors beyond noise, including vibrations, visual impact and shadow flicker, blinking warning lights, and those factors (in addition to noise) explained two thirds of the reported annoyance variability [318]. However, studies using objective measures of sleep have not consistently detected any effect of wind turbine noise on sleep quality or duration. The evidence for impacts on cardiovascular health, mental health, cognitive function and metabolic processes is limited and inconsistent. A sham-controlled trial of infrasound exposure did not pick up any relationship between infrasound and the health factors examined such as somatic and psychiatric symptoms, sound-sensitivity, sleep quality, cognitive performance, and structural MRI [319]. Research on health impact of infrasound is challenging given the ubiquitous nature of infrasound (e.g., from wind, ocean waves, and earth vibrations), and the difficulty in differentiating the actual effects of infrasound from just sensing its presence.

#### 2.1.4 Noise-dependent adverse effects on the intestine via the gut microbiome

**Fig. 13:**
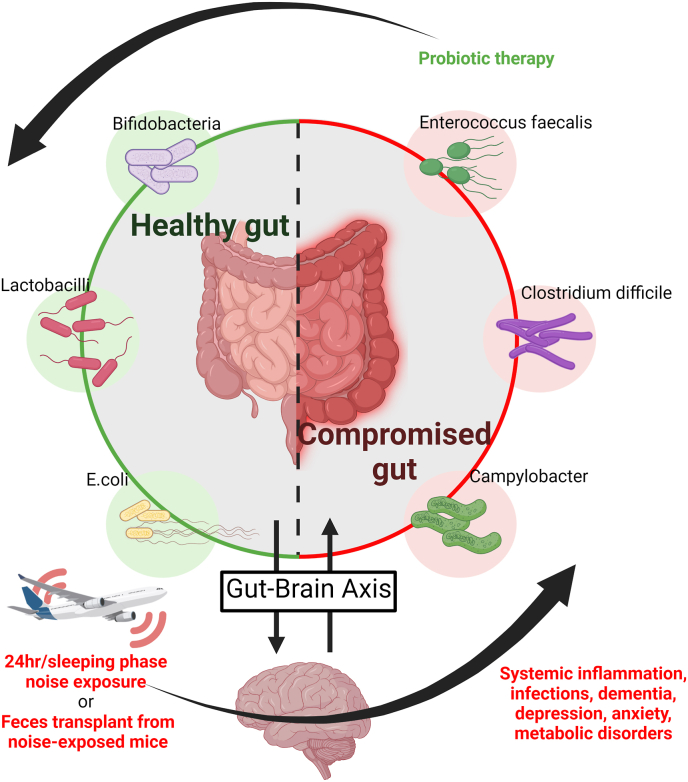
**Noise and the microbiome.** The gastrointestinal microbiome is connected to neuropsychiatric processes via the gut-brain axis, affecting neuropsychiatric disorders, whereas mood and neuropsychiatric health may also affect intestinal inflammatory disease [322,323]. Noise causes neuronal activation with subsequent stress hormone release and has been associated with annoyance, depression, and dementia. Accordingly, noise triggers alterations of the gut-brain axis, leading to a shift to harmful bacteria in the intestine associated with cognitive impairment and Aβ accumulation in a murine model of Alzheimer's disease [324]. Noise also disrupts the equilibrium of intestinal pro-oxidative and antioxidant mechanisms associated with low-grade systemic inflammation in mice [325] and generally causes an imbalance of health-compromising versus -promoting bacteria together with impaired mental health. As a proof-of-concept, these adverse health effects of noise mainly were corrected by probiotic therapy [328]. In contrast, feces transplantation from noise-exposed to unexposed mice induced the above-mentioned health complications [324]. Image was created using Biorender.com by modifying the central scheme from https://de.freepik.com/vektoren-premium/menschlicher-doppelpunktvektor-der-guten-bacterial-flora-illustration_3804027.htm. Reused from Ref. [187] with permission. Copyright © 2021 the authors.

It is now widely accepted that the gut microbiome influences critical biological processes. Disruption therein can influence inflammation and redox signaling in the gastrointestinal tract and, thereby, impact cardiometabolic health (Fig. 13) [320,321]. Gut health appears to influence mood and behavior via a gut-brain axis that can also affect the development of psychiatric disorders and intestinal inflammatory disease [322,323]. Few studies have directly addressed the interaction between noise and the gut microbiome. One study, however, reported that exposure to noise for 4 h during the sleep phase of mice over a period of 30 days, caused alterations in the gut-brain axis (e.g., intestinal tight junction proteins and neurotransmitters) [324]. This study used a mouse model for Alzheimer's disease and reported both cognitive impairment and the accumulation of Aβ, supporting previous studies (see section 2.1.2) linking noise with neuropsychiatric disease. However, the authors also reported decreased levels of the neurotransmitters serotonin and gamma-aminobutyric acid (GABA), increased readouts of inflammation, and impaired tight junction protein expression (claudins, occludin) coupled with changes in the balance of intestinal flora by 16S ribosomal RNA sequencing [324]. Additionally, feces from the noise-exposed mice resulted in an Alzheimer's-like phenotype when transplanted into unexposed mice. Taken together, these results could indicate a mechanism where stress or poor-quality sleep because of noise compromises the intestinal barrier, which then disrupts normal homeostasis in the gut and creates a feedback loop within the gut-brain axis Fig. 13). A second study also reported changes in pro-oxidative and antioxidant pathways and inflammation following noise exposure in mice [325], compounded with similar reports in another rat study that also found evidence that glucose metabolism was disturbed following 30 days of noise exposure [326]. These stressed rats also exhibited elevated glycogen and triglycerides in the liver and IL1β and TNFα in the intestine, indicating a disturbance in both metabolism and inflammation. Another report described a shift in gut species from health-promoting actinobacteria to health-compromising proteobacteria, that was also accompanied by increases in TNF-α and IL-1β and changes in body weight [327].

Apart from proinflammatory and metabolic effects, anxiety-like behavior has also been reported in rats exposed to noise in conjunction with increased corticosterone; once again pointing to increased stress as the ignition for these symptoms [328]. Interestingly, probiotic treatment alleviated these symptoms by restoring the functional interaction of the gut-brain axis (Fig. 13). Treatment of noise-exposed rats with *Lactobacillus rhamnosus GG* prevented cognitive deficits and systemic inflammation by modulating the gut-brain axis (e.g., restoration of behavior and corticosterone levels) [329]. Additionally, a recent study found higher gut microbial diversity in sparrows living in a noisy environment, suggesting that urban sparrows have higher bacterial wealth than their rural counterparts, which was also associated with increased corticosterone and decreased food intake [330]. While studies directly connecting noise, gut health, and disease symptoms are sparse, the results generally agree with mechanisms and symptoms reported in other models, suggesting that the effects of noise are perpetuated throughout the body by nonspecific and broad effects. It also suggests that disturbances in one system (i.e., gut) could exacerbate dysregulation in another system (i.e., vasculature) to promote pathogenesis [331]. In summary, the gut microbiome plays an important role in immune system (de)activation in response to different noise patterns [332].

#### 2.1.5 Noise exposure, aging, and age-related diseases

Substantial experimental evidence in animals supports the role of noise exposure for accelerated aging, and both experimental and clinical studies clearly show the key role of noise exposure promoting age-related diseases. Indeed, the aging process is greatly modulated by the environment. Accordingly, common pathophysiological mechanisms, including mitochondrial oxidative stress, impaired nitric oxide signaling, endothelial dysfunction, and inflammation, have been found in the context of noise exposure [227,333,334] as well as in age-related diseases, such as Alzheimer's and Parkinson's diseases, renal dysfunction, retinopathy, and CVDs [45,285,335]. Furthermore, noise exposure can induce cognitive deficit, especially in spatial learning and memory performance [265,336,337], and chronic noise was found to lead to overproduction of amyloid β and tau hyperphosphorylation in the hippocampus and prefrontal cortex in senescence-accelerated mouse prone 8 (SAMP8) mice [338].

The auditory system is the one most affected by noise exposure upon aging. Indeed, synaptopathic noise (100 dB) accelerates cochlear aging [339]. Noise exposure is considered a major cause of age‐related hearing loss, called presbycusis [340], mainly by related to cochlear synaptic loss [156,341] and sensory cell degeneration of the outer hair cells at the high frequency end of the cochlea. Presbycusis seems to result from damage to mitochondrial DNA and subsequent mitochondrial dysfunction [340], and noise enhances the age-related oxidative stress in the cochlea by increasing superoxide production and lipid peroxidation [342].

### 2.2 Sources of oxidative stress and detection of reactive oxygen species

Clinical and epidemiological data also show an association between typical oxidative stress markers, such as lipid peroxidation products, 3-nitrotyrosine or oxidized DNA/RNA bases, with all significant non-communicable diseases of cardiovascular [[343], [344], [345]], metabolic [39,40] or neurodegenerative origin [42,346] as well as with different forms of cancer [41,347]. Especially ischemic heart disease is tightly linked to mitochondrial H_2_O_2_/O_2_^•−^ formation and oxidative tissue damage [348]. Since oxidative stress is a hallmark of most non-communicable diseases, it is important to know more about the sources of H_2_O_2_/O_2_^•−^, and to gain insight into the mechanisms of oxidative damage, thereby providing better understanding of noise-induced diseases described in the first part of this position paper.

#### 2.2.1 NADPH oxidases

**Fig. 14:**
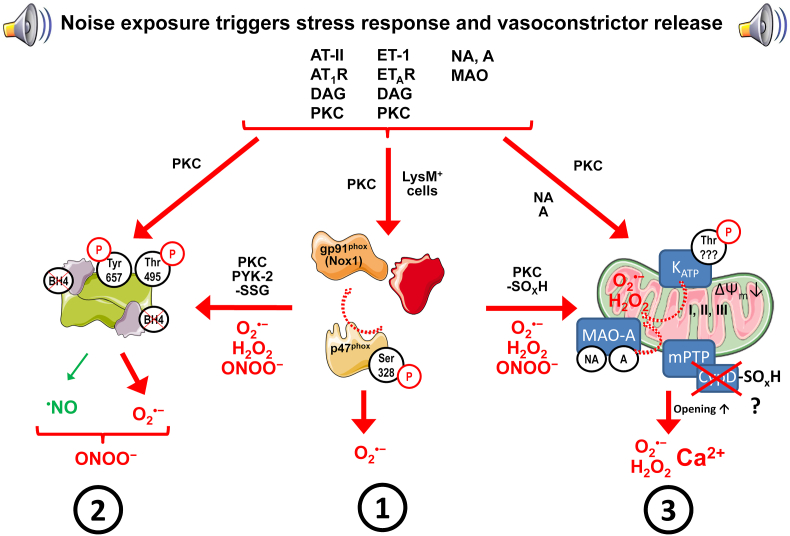
**Oxidative stress pathways activated by noise.** Noise causes stress hormone release (catecholamines and cortisol) and downstream endocrinal activation of vasoconstrictors activating common disease pathways, such as oxidative stress. Angiotensin II (AT-II) and endothelin-1 (ET-1) lead to the formation of diacylglycerol (DAG), a potent activator of protein kinase C (PKC), via their receptors. (1) PKC via phosphorylation of p47^phox^ at serine 328 causes activation of the phagocytic NADPH oxidase (NOX-2) and potentially NOX-1. The expression of NOX-2 is upregulated by noise-triggered immune cell infiltration (lysozyme M-positive (LysM^+^) cells) and systemic inflammatory conditions. NOX-2 (and NOX-1, especially in the brain) produces superoxide (O_2_^•−^) and via dismutation also hydrogen peroxide (H_2_O_2_). NOX-4 was not changed by noise and NOX-5 (relevant for humans) was not studied so far. (2) Dysfunction of endothelial nitric oxide synthase (eNOS) is mediated by noise-dependent activation of PKC and phosphorylation of threonine 495. Alternatively, NOX-2-dependent ROS formation may activate PKC [349] and protein tyrosine kinase 2 (PYK-2) [350,351], causing adverse phosphorylation at tyrosine 657 and threonine 495. Uncoupling of eNOS may be induced by noise-driven oxidative depletion of tetrahydrobiopterin (BH_4_) and S-glutathionylation (-SSG) of eNOS by ROS originating from NOX-2 [352]. Semi-uncoupled eNOS may represent a potent source of peroxynitrite. (3) Noise also leads to mitochondrial ROS formation, generating both O_2_^•−^ and H_2_O_2_. Noradrenaline (NA) and adrenaline (A) originating from sympathetic activation are substrates of monoamine oxidases (MAO) that produce H_2_O_2_. PKC seems to activate the mitochondrial K_ATP_ channel by phosphorylation of a threonine residue with subsequent depolarization of the mitochondrial membrane (ΔΨ_m_↓) and O_2_^•−^ formation from respiratory complexes I, II and III. Mitochondrial H_2_O_2_/O_2_^•−^ and calcium are released to the cytosol upon the mitochondrial permeability transition pore (mPTP) opening (e.g., by thiol oxidation of the regulatory subunit cyclophilin D (CypD) [353]). K_ATP_ channel activation and mPTP opening can also be stimulated by redox-crosstalk with H_2_O_2_ (probably also O_2_^•−^ via peroxynitrite) derived from NOX-2 [197]. So far, there is no evidence for the role of xanthine oxidase in noise's non-auditory (indirect) effects. This scheme contains images from Servier Medical Art by Servier, licensed under a Creative Commons Attribution 3.0 Unported License.

NOX-2 (gp91phox), which was referred to frequently in the previous sections, is the enzymatic weapon used by myeloid phagocytes to combat immune insults. This enzyme allows actors within the innate immune system to produce toxic oxidants to destroy engulfed pathogens-a critically important action in host defense. However the same machinery can also be inappropriately deployed and activated in response to sterile inflammation; a dysregulation that contributes to endothelial dysfunction [354,355], hypertension [356,357], IHD [358], and atherosclerosis [359]. Whereas the involvement of different NADPH oxidase isoforms (e.g., NOX-1, NOX-4, NOX-3, and DUOX-2) in noise-induced hearing loss is well established [[360], [361], [362]], only a few studies have provided information about the role of NADPH oxidases in the non-auditory (indirect) pathology. Upon exposure to noise, NOX-2 protein and *Nox2* mRNA levels are consistently upregulated in the murine aorta and heart [14,248,281]. Also, a more pronounced activation state of NOX-2 was reported for noise-exposed mice, which was driven by angiotensin-II dependent diacylglycerol-mediated PKC activation with subsequent Ser328 phosphorylation of p47^phox^; the cytosolic regulator of NOX-2 (Fig. 14) [16]. Accordingly, the oxidative burst in whole blood of noise-exposed mice, which is mainly driven by phagocytic NADPH oxidase in leukocytes, was more pronounced than in unexposed mice [16]. The latter finding was further supported by slightly higher serum levels of soluble NOX-2-derived peptide (sNox2-dp, an ELISA-based measure of NOX-2 activation) in mice upon noise exposure [245].

Evidence of oxidative stress is readily detectable in the aorta, heart and brains from mice exposed to noise. Importantly, mice with a genetic deletion of *Nox2* are protected from this oxidative stress as well as from the subsequent microvascular dysfunction in the cerebral microvessels and leukocyte-endothelial cell interaction [16]. Moreover, proteomic analyses demonstrated no noise-induced increase in inflammatory signaling in plasma from *Nox2* knockout mice, a situation that was markedly different in samples from *Nox2* expressing mice [363]. The enzyme can be targeted pharmacologically and the NOX-2 inhibitor, GSK2795039, quenched ROS signals in cerebral cryo-sections of noise-exposed mice [16], which overall points to this enzyme fueling the proverbial oxidative fire that arises. Further support for a central role of NOX-2 in noise-mediated pathophysiology comes from a study showing an additive upregulation of NOX-2 protein in noise-exposed hypertensive mice, mirrored by additive increases of aortic and cardiac superoxide formation [247]. A similar add-on effect of noise on NOX-2 expression and activity was also reported in mice that had experienced MI before noise exposure [248]. It was also shown that NOX-2-expressing cells were primarily responsible for noise-induced cardiovascular and cerebrovascular damage through a selective ablation protocol targeting cells expressing lysozyme M (LysM) (e.g., monocytes and macrophages) by overexpression of an inducible diphtheria toxin receptor [281]. By low dose treatment with diphtheria toxin these inflammatory cell subsets can be specifically killed, thereby removing the most prominent NOX-2 expressing cells. As a result, blood pressure, endothelial function, and oxidative stress parameters were not adversely affected in mice without LysM^+^ cells exposed to noise.

In the brains of noise-exposed mice, aggravated NOX-2 activation was observed in the form of phosphorylation of the significant cytosolic regulator of NOX-2, p47^phox^, at serine 328, as well as activation of PKC, as measured by phosphorylation of the myristoylated alanine-rich C-kinase substrate (MARCKS) [15,16]. NOX-2 protein expression was also increased in cerebral micro-vessels [364]. In addition, *Nox1* mRNA expression was increased in the brains of noise-exposed mice [16], an observation mirrored by enhanced 3-nitrotyrosine, ROS formation, and impaired microvascular function in cerebral arterioles [363]. *Nox1* upregulation was also found in isolated lung endothelial cells [14]. In contrast, no upregulation of vascular NOX-4 in mice exposed to noise with low SPL (<80 dB(A)) was reported, although a tendency of higher NOX-4 levels in the brains of noise-exposed mice was noted [16].

Studies have also observed that noise-exposed mice had increased ET-1 expression in the aorta and that ET-receptor signaling was exacerbated as envisaged by more pronounced ET-1-dependent vasoconstriction [14,16]. Endothelin-1 exacerbates oxidative burden by directly inducing NOX-2 expression [365,366] and directing ET-receptor-dependent NADPH oxidase-derived O_2_^•−^ formation and subsequently H_2_O_2_ by dismutation. This second action can be illustrated by ex vivo ET_A_-receptor blockade of vascular cells [365,367,368] or white blood cells [36], resulting in reduced NOX-dependent O_2_^•−^ and H_2_O_2_ formation, as is also the case in hypertension [[369], [370], [371]]. Alternatively, catecholamines can activate astrocytes, microglia, and consequently NOX-2 [372].

#### 2.2.2 Mitochondria

Though NOX enzymes are essential generators of O_2_^•−^, H_2_O_2_ and other ROS, they are not their sole source. Mitochondria are well-known producers of O_2_^•−^ and H_2_O_2_ and an important pharmacological target for treating IHD [373,374] but may have on impact on development of hypertension as well [375,376]. Interestingly, a study has found that some of the oxidative burden observed after noise exposure may be ascribed to mitochondria: rats exposed to low-frequency noise (≥90 dB(A), <500 Hz) had cardiac fibrosis, enlarged cardiac mitochondria and reduced connexin 43 content, indicating mitochondrial damage [377]. Mitochondrial connexin 43 content affects ROS formation [378,379]. Mitochondrial swelling, matrix dilution, cristolysis, and DNA damage have been reported in response to very loud noise (100 dB(A)) and linked to high noradrenaline levels, MAO activity, and disturbed mitophagy [380,381], possibly negatively impacting permeability transition (e.g., mPTP) and calcium handling [382].

Two different isoforms of MAO exist, namely MAO-A and MAO-B, both of which are located at the outer mitochondrial membrane (reviewed in Ref. [383]). Species- and cell type-dependent expressed MAO isoforms differ. Taking the heart as example, in rats MAO-A predominates at adulthood while in adult mice MAO-B dominates. Interestingly, in rat hearts, MAO-B activity also predominates up to an age of 2–3 weeks, most likely since MAO-B expression increases under mechanical strain as compared to the quiescent situation [383]. Human hearts contain both MAO isoforms, but with more, albeit moderate, expression for MAO-A in cardiomyocytes. The two MAO isoforms have common substrates, such as dopamine but also specific substrates: MAO-B can metabolize 1-methyl histamine, produced by the histamine-N-methyltransferase, while MAO-A metabolizes serotonin (or 5-hydroxytryptamin, 5-HT) and catecholamines. Interestingly, MAO-A contributes to serotonin- but not norepinephrine-dependent damage of rat ventricular myocytes [384]. MAO requires flavin adenine dinucleotide as a cofactor that is reduced by the reaction and subsequently re-oxidized by molecular oxygen, generating hydrogen peroxide. MAO can also form reactive aldehydes, such as 4-hydroxynonenal, as byproduct of catecholamine metabolism through cardiolipin peroxidation inside mitochondria in primary cardiomyocytes. Deleterious effects of 4-hydroxynonenal are physiologically prevented by its rapid metabolism [385], furthermore facilitated by the activation of mitochondrial aldehyde dehydrogenase 2 [386]. An increased expression/activity of MAO occurs during aging and with different cardiovascular diseases. While the underlying mechanisms of MAO upregulation are still unclear, one potential factor contributing to increased MAO expression/activity might be increased substrate availability.

An increased sympathetic tone increases plasma norepinephrine and epinephrine concentrations. Serotonin concentrations are increased during different disease states (for review, see Ref. [387]) and part of the increase has been attributed to altered platelet function [388]. Histamine co-localizes with norepinephrine in neurons [389] and is enclosed in cytoplasmic granules of mast cells, which lie adjacent to blood vessels and between cardiomyocytes [390], and mast cell degranulation might occur under stress conditions [391]. Activation of MAO contributed to development of endothelial dysfunction [392] and irreversible cardiomyocyte injury *in vitro* [393] and *in vivo* [394], also the latter was restricted to males only.

In the cochlea, loud noise (100–120 dB(A)) also activates SHC-transforming protein 1 (SHC1, p66^Shc^), a mitochondrial source of oxidative stress. Cochlear vascular dysfunction and transient noise-induced hearing loss subsequently arose [395]. p66^Shc^ is involved in the regulation of vascular tone [396], also during aging [397], with little effect on irreversible cardiac damage under stress conditions [398] (also reviewed in Refs. [399,400]).

Some studies have reported that noise exposure <80 dB(A) can lead to mitochondrial ROS formation in the brains of noise-exposed mice [16] as well as higher superoxide formation rates in cardiac mitochondria [247]. An additive increase in mitochondrial superoxide levels was also seen in the hearts of noise-exposed mice with MI in conjunction with impaired mitochondrial respiration and oxygen handling [248]. Of note, whereas cerebral ROS formation (most probably superoxide) upon 1 or 2 days of noise exposure was fully responsive to NOX-2 inhibition or genetic deletion of Nox2, the ROS signal after 4 days of noise was still visible in the absence of NOX-2 activity, suggesting that mitochondrial ROS formation may play a role following chronic noise exposure [16]. Potential mechanisms of mitochondrial O_2_^•−^ and H_2_O_2_ formation in response to noise are summarized in (Fig. 14). Catecholamines released upon noise-induced sympathetic activation could lead to hydrogen peroxide formation by MAO, enzymes that are potent mitochondrial H_2_O_2_ sources using noradrenaline or adrenaline as substrates [401]. Another pathway of noise-induced mitochondrial O_2_^•−^ and H_2_O_2_ formation may consist of the PKC-dependent activation of the mitochondrial ATP-sensitive potassium channel (K_ATP_) channel by phosphorylation at a threonine residue [402,403] with subsequent depolarization of the mitochondrial membrane potential leading to higher superoxide formation rates from respiratory complexes I, II, and III [197]. Finally, ROS-induced mPTP opening by thiol oxidation of the significant regulator cyclophilin D [353] may represent a mechanism for how noise could promote the release of mitochondrial calcium, O_2_^•−^ and H_2_O_2_ to the cytosol, activating redox- and calcium-sensitive kinases such as PKC [197,404]. However, it is unclear to what extent this mechanism contributes to noise-mediated pathophysiology.

#### 2.2.3 Uncoupled nitric oxide synthases

Due to the excessive superoxide formation in noise-exposed animals, endothelial NOS (eNOS) in the aorta (and nNOS in the brain) uncouples, which means that it transforms into a source of O_2_^•−^ and H_2_O_2_ rather than, or in addition to ^•^NO source (Fig. 14) [45,405]. NOS uncoupling was previously demonstrated in tissues of noise-exposed mice by dihydroethidium staining in the presence of the eNOS inhibitor N^G^-nitro-l-arginine methyl ester (l-NAME) [16,247,281]. eNOS is redox-sensitive because of its reliance on a readily oxidizable cofactor, tetrahydrobiopterin (BH_4_). Without BH_4_, eNOS cannot produce ^•^NO, but instead produces O_2_^•−^ [355]. The concomitant formation of ^•^NO and O_2_^•−^ by uncoupled eNOS generates peroxynitrite, which in term reacts with proteins to result in their tyrosine nitration i.e. the appearance of 3-nitrotyrosine-positive proteins in the vascular wall of conductance and resistance vessels [14,248,363]. eNOS uncoupling diminishes ^•^NO bioavailability in the aortas of noise-exposed mice as determined by the direct quantification of ^•^NO using electron spin resonance spectroscopy [14] or via plasma nitrite levels [245,247]. eNOS activity is also regulated by the phosphorylation of the enzyme and a reduction in the phosphorylation of an activity promoting site (Ser1177) was also reported in hypertensive mice exposed to 7 days of aircraft noise [247]. Uncoupling of eNOS by noise at the same time as activating Ser1177 phosphorylation seems contradictory but may represent a futile counter-regulatory process. Increased plasma nitrite levels in noise-exposed rats have also been attributed to the action of inducible NOS [206], as is common in inflammatory conditions. Taken together, dysregulation or uncoupling of eNOS is a central event in the pathophysiology of CVD and is closely correlated with impaired endothelial function (see section 2.1.1). eNOS S-glutathionylation was also increased in the aorta and heart of noise-exposed mice [14]. The latter effect was not observed in *Nox2*–deficient mice [16] and was aggravated in noise-exposed hypertensive or ischemic/reperfused mouse hearts [247,248]. eNOS activity can be inhibited by its phosphorylation on inhibitory sites that shut down ^•^NO and O_2_^•−^ production completely but no study has yet addressed the impact of noise on these mechanisms. Still, the kinases that phosphorylate the inhibitory sites in eNOS (i.e., PKC, protein tyrosine kinase 2 (PYK-2)) are redox-activated, so an involvement of this mechanism upon noise exposure is highly probable [45]. As a direct effect of loud noise in the cochlea of guinea pigs, inducible and endothelial NOS levels were upregulated, which likely contribute to nitrosative and oxidative stress [406].

**Fig. 15:**
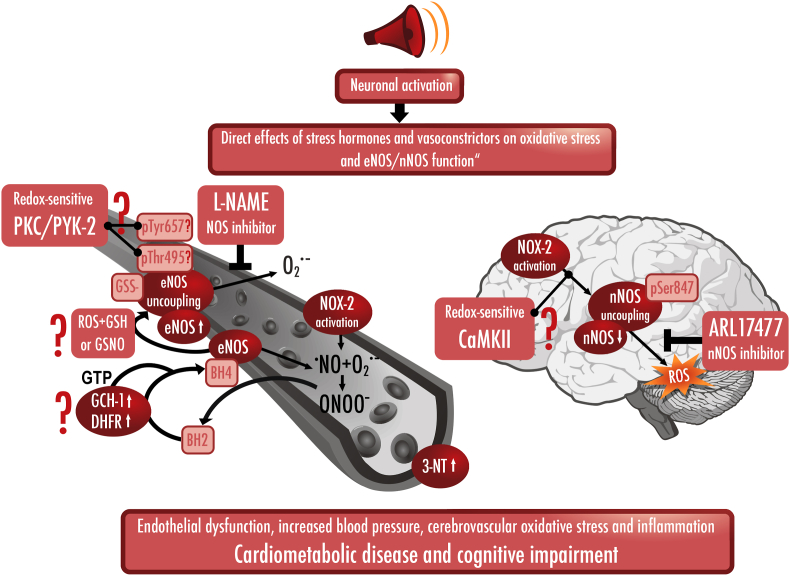
**Noise exposure causes eNOS and nNOS uncoupling.** Superoxide formation induced by noise (e.g., NOX-2 activation) causes oxidative break-down of ^•^NO, leading to peroxynitrite formation explaining increased protein tyrosine nitration (also iNOS-derived ^•^NO contributes) as well as impairment of ^•^NO/cGMP signaling. Despite upregulation of eNOS and the tetrahydrobiopterin (BH_4_)-generating enzymes GTP-cyclohydrolase 1 (GCH-1) and dihydrofolate reductase (DHFR), diminished ^•^NO bioavailability was observed in the aorta of noise-exposed mice. BH_4_ is an essential cofactor of eNOS (but also of iNOS), and oxidative depletion by ROS (e.g., peroxynitrite) to dihydrobiopterin (BH_2_) causes uncoupling of all NOS isoforms. BH_4_ levels were not measured upon noise exposure, but upregulation of GCH-1 and DHFR obviously cannot compensate for the loss of function of eNOS in this setting. Therefore, eNOS was found to be dysfunctional or uncoupled, supported by S-glutathionylation (GSS-modification) by either peroxynitrite or H_2_O_2_/reduced glutathione (GSH) or S-nitros(yl)ated glutathione (GSNO) reaction. eNOS S-glutathionylation is an accepted marker of eNOS uncoupling, and uncoupled eNOS after noise exposure was detected at the molecular level by endothelial ROS formation that was sensitive to inhibition by the NOS inhibitor l-NAME. Although not measured in tissues of noise-exposed animals so far, the inactivating phosphorylation of eNOS at threonine 495 or tyrosine 657, mediated by redox-activated protein kinase C (PKC) [349] and protein tyrosine kinase 2 (PYK-2) respectively [350,351], would be conceivable under noise-induced oxidative stress conditions. In the brain, oxidative stress induction (e.g., NOX-2 activation) by noise caused nNOS uncoupling as envisaged by cerebral ROS formation that was sensitive to inhibition by the selective nNOS inhibitor ARL-17477 as well as phosphorylation at serine 847 (previously reported for uncoupled nNOS). Phospho-Ser847 in nNOS is introduced by calcium/calmodulin-dependent protein kinase (CaMKII) activated by ROS, e.g. H_2_O_2_. The adverse redox regulation of eNOS and nNOS by noise-induced oxidative stress promotes the development of cardiometabolic disease and cognitive impairment previously reported for noise exposure in clinical/epidemiological studies. Reused from Ref. [45] with permission.

The NOS enzyme that is preferably expressed in neuronal tissues, nNOS, appears to respond slightly differently to noise exposure than eNOS. Noise-exposed mice presented with downregulation and uncoupling of nNOS, an event that did not occur in *Nox2* knockout mice [16]. Murine cerebral nNOS was also phosphorylated on serine 847 [16], which is an inhibitory site [407], possibly indicating an uncoupled nNOS enzyme [408]. This site is also redox-sensitive via calcium/calmodulin-dependent protein kinase [408]. Some support for the uncoupling of nNOS in the noise-exposed brain comes in the form of ex vivo inhibition with ARL-17477, which partially blocked the oxidative stress signal in cerebral tissue of noise-exposed mice [16]. The presence of noise-induced O_2_^•−^ in the tissue may deplete vasodilatory ^•^NO, resulting in a neuroinflammatory phenotype and loss of the protective antioxidant transcription factor *Foxo3,* exacerbating the oxidative imbalance and potentiating endothelial dysfunction in the brain [16,281]. These initial steps can produce a pro-oxidative/inflammatory phenotype explaining the observed impairment of cognitive development (memory/learning) of school children exposed to high noise levels [286], similar to the learning and memory impairment reported in adults [305]. An impact of dysregulated nNOS on impairment of cognitive and memory function seems feasible in light of direct effects of neuronal ^•^NO on these processes or indirectly by the regulatory role of ^•^NO on glutamate signaling Fig. 15 illustrates the mechanisms influencing the NOS coupling status in noise-exposed rodents.

#### 2.2.4 Detection of reactive oxygen species

##### 2.2.4.1 Superoxide

The most used probe for the *in vivo* and *in vitro* detection of superoxide radical anion (O_2_^•–^) is dihydroethidium (DHE, also known as hydroethidine, HE) [409,410]. This probe forms an O_2_^•–^-specific red fluorescent product, 2-hydroxyethidium (2-OH-E^+^). It should be noted, however, that 2-OH-E^+^ is not the only fluorescent product of DHE oxidation, and ethidium (E^+^) is another fluorescent product that is formed during the oxidation of DHE by other oxidants. Also, heme proteins, including cytochrome *c*, are known to efficiently oxidize DHE to ethidium (E^+^) and several dimeric products [411]. Therefore, increased red fluorescence is expected during apoptosis and is associated with mobilization of mitochondrial cytochrome *c*. This implies the need to selectively detect and quantify 2-OH-E^+^, typically accomplished using chromatographic techniques (HPLC with fluorescence, electrochemical or mass spectrometric detection) allowing separation and detection of different oxidation products [412,413]. Derivatives of DHE targeted to mitochondria (MitoSOX Red, MitoNeoD) or the extracellular space (hydropropidine) have been also reported [[414], [415], [416]]. The chemistry of those probes resembles the chemistry of DHE, and therefore the same recommendations regarding the detection of the O_2_^•–^-specific product apply [410,411,417,418].

Lucigenin has been used for chemiluminescent O_2_^•–^ detection for more than two decades [419]. While some studies showed a good correlation with the production of 2-OH-E^+^ from DHE [420] without such a parallel assay, the lucigenin-derived chemiluminescence may be difficult to interpret, as the probe reacts very slowly with O_2_^•–^. It may act as a redox cycler in the presence of flavoproteins, resulting in O_2_^•–^production [421]. Nitroblue tetrazolium and more recently water-soluble tetrazolium (WST-1) probes are being used as colorimetric stains for superoxide, as they form formazan-type reduced products, easily detectable by spectrophotometry [422,423]. This type of probes is typically used for cellular and cell-free assays *in vitro*. The major limitation is the possibility of superoxide independent reduction of tetrazolium salts to formazans, requiring further studies on the involvement of superoxide in the reduction of the probes [424]. Also, the redox cycling activity of nitroblue tetrazolium to produce superoxide has been reported and should be considered when using the probe [425]. While still awaiting a complete chemical characterization and biological validation, additional promising chemical probes for O_2_^•–^, including triflate-, phosphonate- and more recently tetrazine-based sensors, have been developed [[426], [427], [428]].

A further method to measure superoxide is by electron spin resonance [429]. Superoxide is able to form a characteristic spin adduct with 5,5-dimethyl-1-pyrroline-N-oxide (DMPO) forming DMPO-OOH, which is easily detectable by electron spin resonance. Problems of this method are the slow reaction of superoxide with DMPO, the low concentrations of the spin adduct formed (also due to its degradation by biological antioxidants such as vitamin C or glutathione) and the relatively complicated analytical equipment required.

##### 2.2.4.2 Hydrogen peroxide

Detection of H_2_O_2_ is typically accomplished using peroxidase-dependent probes, including reduced fluorescein and rhodamine probes (DCFH, DHR), and Amplex Red [427,430]. Peroxidase-dependent assays may be used to analyze extracellular H_2_O_2_ or in cell-free systems, including isolated mitochondria, and Amplex Red is the recommended probe [431]. The use of DCFH and DHR probes should be avoided, as those probes do not react directly with H_2_O_2_, can be oxidized by cytochrome *c* mobilized during apoptosis [432], and may produce O_2_^•–^ during their conversion to the fluorescent product [421,433]. Using boronate-based probes may allow the detection of H_2_O_2_, ONOO^−^ or other boronate-reactive oxidants [[434], [435], [436]]. With proper experimental design and/or detailed profiling of the oxidation/nitration products, the oxidants involved may be identified [437,438]. The advantages of boronate probes include direct reaction with the oxidants, resistance to peroxidatic oxidation, and a wide range of detection modalities, including bioluminescence and other *in vivo*-compatible techniques [439,440].

##### 2.2.4.3 Other ROS

Other probes typically used for general assessment of oxidative stress and burst include luminol and analogs, such as L-012 probe [441]. Although initially assumed to be specific for superoxide, such probes are prone to peroxidase-catalyzed oxidation, and may produce superoxide in such systems [442]. As inflammation may lead to increased expression of peroxidases (e.g., myeloperoxidase), increased oxidation of such probes should not be used as a sole indication of increased ROS level but should be accompanied by additional assays to determine the role of peroxidases and preferably the identity of the oxidant(s) involved. The constant development of new redox probes and assays opens an exciting opportunity to decipher the role of specific ROS in traffic noise-induced pathologies. An overview on recommended methods of ROS detection can be found in Refs. [443,444].

### 2.3 Redox-related pathophysiological mechanisms

#### 2.3.1 Stress response

**Fig. 16:**
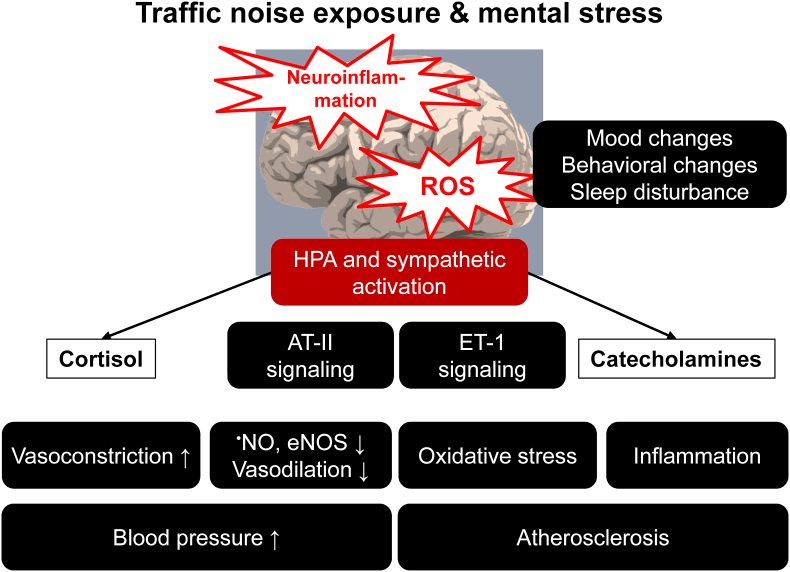
**Noise triggers a neuronal stress response, leading to oxidative stress, inflammation, and vasoconstriction.** Traffic noise results in mental stress that causes activation of the sympathetic nervous system and the hypothalamic-pituitary-adrenal (HPA) axis with subsequent release of catecholamines (e.g., adrenaline, noradrenaline) and cortisol, a glucocorticoid. This process is accompanied by neuronal activation via inflammation and oxidative stress (exacerbated ROS formation, mostly O_2_^•−^ and H_2_O_2_), which promotes neuropsychiatric and sleep disorders. Sympathetic and HPA axis activation leads to adverse signaling via catecholamines, angiotensin II (AT-II), endothelin-1 (ET-1), and glucocorticoids, leading to an inflammatory phenotype, oxidative stress, downregulation of eNOS and diminished nitric oxide (^•^NO) bioavailability, and vasoconstriction. These adverse signaling events contribute to increased blood pressure, atherosclerosis, and higher cardiovascular risk. Redrawn and modified from Refs. [29,43] with permission.

As described in section 2.1, a primary general mechanism that noise operates through is the activation of a stress response with subsequent induction of vascular and cerebral inflammation and oxidative stress by the upstream pathophysiological mechanisms shown in Fig. 2, Fig. 9, Fig. 16 (reviewed previously [28,29]). The response to noise is immediate, not requiring long duration of exposure to elicit a physiological response (e.g., exposure for 30 min (85 dB(A)) has been found to increase ACTH and corticosterone in a dose-dependent manner) [445,446]. Furthermore, noise-induced activation of the stress response in rats (80–100 dB(A), 8 h/day in 20 days) involved increased levels of plasma corticosterone, adrenaline, noradrenaline, and ET-1, coinciding with elevated levels of malondialdehyde, a readout of oxidative stress, and increased heart rate and arterial blood pressure [206]. Rats exposed to moderate noise (70 or 85 dB(A), 6 h/day for 3 months) were found to have a dose-dependent increase in corticosterone levels and lipid peroxidation accompanied by morphological changes in the heart and inflamed areas of the pericardium and dilated veins (70 dB(A)), with even greater changes in the 85 dB(A) group [205]. Additionally, rats chronically exposed to noise had upregulated *Crh* and *Crhr1* (corticotropin-releasing hormone and its receptor) mRNA levels in the amygdala [447].

The interplay between stress hormones and vasoconstrictors offers a rationale for the disruption of vascular tone triggered by noise exposure (Fig. 16). When noise exposure occurs during sleep, it leads to sleep fragmentation and excessively short sleep intervals [448], culminating in psychological stress. The increased level of stress hormones and disruption of the circadian rhythm ignites cerebral oxidative stress, involving heightened angiotensin II signaling and activation of NOX-2 [310]. Such factors collectively contribute to the inflammation of the brain's microvasculature. Animals subjected to noise exposure exhibit elevated levels of circulating angiotensin II as well [14,449]. SNS activation driven by NOX-2-induced oxidative stress proceeds to activate both HPA and RAAS [450,451] and, in turn, catecholamines initiate oxidative stress through pathways such as the promotion of MAO activity [401] or activation of astrocytes, microglia, and NOX-2 [372]. In supporting the concept of a RAAS–ROS–SNS axis concept, administrating of a NOX inhibitor reduced blood pressure, angiotensin II, and noradrenaline levels in hypertensive mice [452]. In contrast, inhibition of type 1 angiotensin II receptor and blockade of angiotensin-converting enzyme decreased oxidative stress within the heart and vasculature [453,454]. In mice, exposure to aircraft noise (72 dB(A) over 4 days) increased the expression of ET-1 in the aorta, a potent vasoconstrictor that triggers NOX-2 activity [14,16,365], which is, in part, dependent on RAAS [198].

These findings supply molecular and pathophysiological insights that address the appearance of endothelial dysfunction and hypertension observed in animal models exposed to (aircraft) noise. Central to this process is NOX-2-triggered oxidative stress and inflammation, alongside the disruption of circadian rhythm due to sleep fragmentation and deprivation. The robust support from animal data underlines the pivotal role of stress response pathways in the detrimental cardiovascular and cerebral consequences of noise exposure in humans. It offers detailed molecular mechanisms regarding the sequence of events within the brain and the stress response axis.

#### 2.3.2 Inflammation

**Fig. 17:**
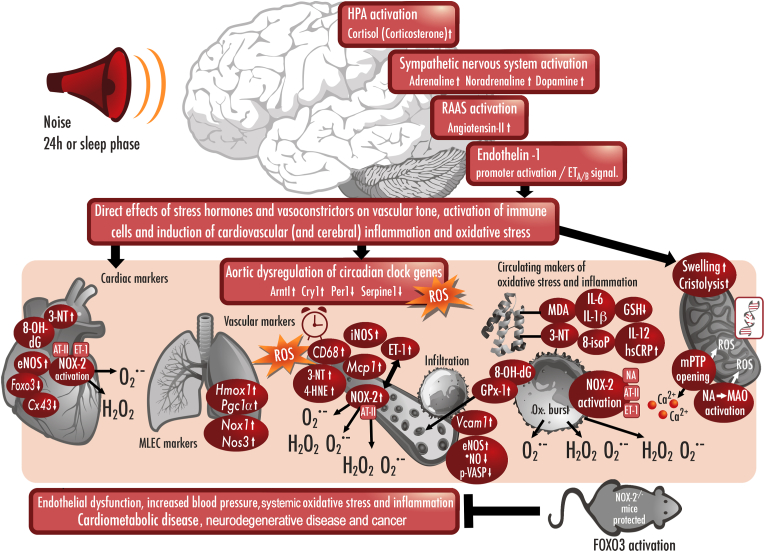
**Linkage between exposure to noise and inflammation and oxidative stress.** First-line neuronal events in response to noise exposure are sleep disturbance (when exposed during the sleep phase) and stress response reactions via activation of the hypothalamic-pituitary-adrenal (HPA) axis and the sympathetic nervous system. This leads to the release of stress hormones (glucocorticoids and catecholamines) and secondary activation of the cerebral (and systemic) renin-angiotensin-aldosterone system (RAAS) as well as endothelin-1 expression. These potent triggers of inflammation and oxidative stress activate NOX-2 via protein kinase C (PKC) and p47^phox^ phosphorylation in the brain, increase expression of markers of inflammation, lipid peroxidation, and cause downregulation of neuronal nitric oxide synthase (nNOS) and loss of antioxidant genes, such as catalase (*Cat*) and forkhead box O3 (*Foxo3*) transcription factor. These changes induce a neuroinflammatory phenotype with cerebral oxidative stress. These stress hormones and vasoconstrictors lead to similar adverse changes in the cardiovascular (and pulmonary) system and increase the risk of cardiometabolic and potentially other non-communicable diseases, such as diabetes or cancer. The HPA axis, sympathetic nervous system, RAAS, endothelin-1 expression, and neuroinflammation are redox-regulated, and vice versa, can induce oxidative stress via NOX-2 activation and other sources. AT-II, angiotensin-II; CRH, corticotrophin-releasing hormone; ACTH, adrenocorticotrophic hormone. Reused from Ref. [45] with permission.

Inflammation has been associated with acute stress and sleep disturbance, noise exposure, and injury [455]. This occurs through the immediate activation of the SNS upon exposure to a stimulus, followed by activation of the HPA axis within minutes [455]. The subsequent release of stress hormones gives rise to systemic and tissue-specific inflammation, with elevated levels of IL-6, IL-1β, proinflammatory monocytic infiltration into tissues [43,456], and oxidative stress [29]. Stressors such as noise-induced sleep deprivation can induce cerebral oxidative stress orchestrated through angiotensin-II signaling and NOX-2 activation, producing microvascular and neuronal inflammation [310], likely from a microglial source. Consequently, it is plausible that noise-induced O_2_^•−^ and H_2_O_2_ generation fosters an inflammatory profile in the heart, blood vessels, brain, and other organs. Crucial mediators of inflammatory responses, such as the NLR family pyrin domain containing 3 (NLRP3) inflammasome and high-mobility group box 1 protein (HMGB1) are activated under conditions of oxidative stress through redox switches and redox-sensitive transcription factors like nuclear factor kappa B (NFκB) [457,458], which likely underpins noise-triggered inflammation in exposed mice [14,16,247,281,363]. This oxidatively-fueled inflammation could potentially explain the shift towards a pro-atherothrombotic phenotype in the plasma proteome of healthy human subjects exposed to train noise [234]. Epigenetic alterations promoting immune cell activation, CRP expression [459,460], and inflammatory coronary atherosclerosis related to heightened stress-associated neural activity involving the amygdala [47,202,203], could also stem from this complex interplay. In two studies, noise-induced inflammation was prevented in mice with *Nox2* deletion [16,363]. Furthermore, antioxidant pharmacological activation/induction of the nuclear factor E2 related factor-2 (NRF-2)/heme oxygenase 1 (HO-1) axis [461], probiotic therapy [329], and treatment with the antibiotic minocycline [462] all been shown to inhibit noise-induced inflammation. The hypothesized mechanisms behind noise-induced inflammation are presented in Fig. 17.

As previously discussed, noise initiates neuroinflammation and Alzheimer's disease pathology in rodent studies [270], which is in line with results from rodent studies with exposure to low-level noise (73 dB(A)). This leads to increased levels of circulating cytokines (IL-6, IL-1β), aortic *iNOS,* monocyte chemotactic protein 1 (*MCP-1* or *CCL-2),* cluster of differentiation 68 (*CD68*) mRNA levels, cardiac *TNF-α, IL-6, IL-1β,* interferon γ (*IFN-γ*)*, MCP-1,* cell adhesion molecules such as vascular cell adhesion molecule 1 (*Vcam-1*)*,* and vascular infiltration of immune cells (Fig. 17) [14,16,248]. This was accompanied by neuroinflammation characterized by astrocyte activation and higher cerebral *CD68, IL-6* and *iNOS* levels (Fig. 9) [16] and upregulated expression of *Vcam-1, NFκB* (*CD40L*, NLRP3 and thioredoxin interacting protein (TXNIP) by trend) [245,281]. Other circulating cytokines and chemokines also seemed upregulated in mice exposed to low-level noise as measured using a cytokine array [15]. The aggravated systemic inflammation in noise-exposed animals is also reflected by enhanced oxidative burst by whole blood leukocytes [16,245,463]. In addition, there is evidence that noise additively increases markers of inflammation in mice with pre-established hypertension [247], on top of experimental MI [248], or with particulate matter co-exposure [15]. In addition, heat stress and noise may synergistically increase inflammation [464].

**Fig. 18:**
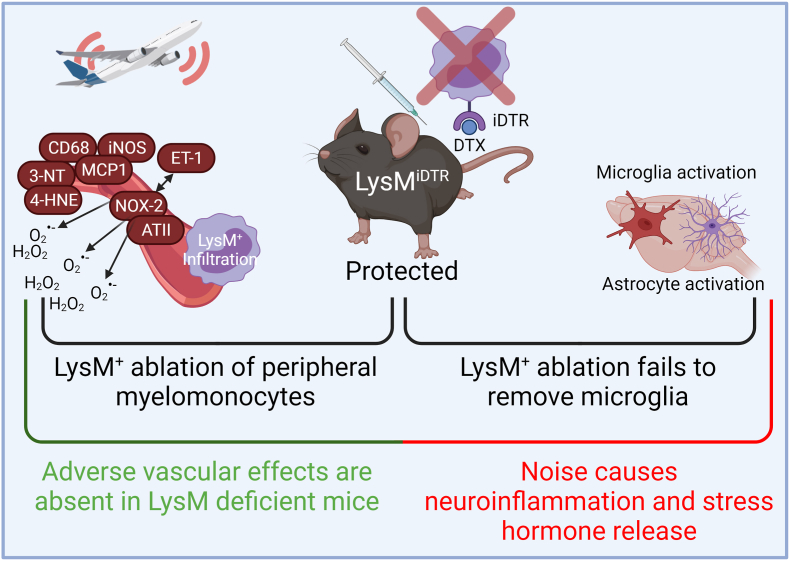
**Role of LysM-positive myelomonocytic cells for noise-induced inflammation and damage.** Genetic ablation by treatment of mice with LysM-positive cell (myelomonocytic) transgenic expression of an inducible diphtheria toxin receptor (LysM^iDTR^) with low dose diphtheria toxin [465]. Mice free of LysM-positive cells showed no noise-dependent vascular infiltration of monocytes, macrophages, or granulocytes. They preserved endothelial function, normal blood pressure, and no aortic oxidative stress, indicating that LysM-positive cell ablation protects the vasculature from noise-induced damage. In contrast, microglia in the brain of LysM^iDTR^ mice were not ablated by diphtheria toxin, and noise-induced neuroinflammation, cerebral oxidative stress and release of stress hormones were not prevented. The image was created using Biorender.com. Modified from Ref. [187], based on data in Ref. [281] with permission. Copyright © 2021 the authors.

Molecular support for a noise-induced vital crosstalk between the brain, the heart, and the vessel was provided through a selective ablation protocol targeting cells expressing lysozyme M (LysM) [465]. Peripheral mononuclear phagocytes (monocytes and macrophages) are characteristically LysM^+^, while microglia lack or have minimal LysM. As a result, in the LysMCre^iDTR^ model, administration of diphtheria toxin kills and removes peripheral mononuclear phagocytes but not microglia. LysM^+^ cell-deficient mice were protected from noise-induced rise in blood pressure, endothelial dysfunction, and oxidative stress in non-central tissues (Fig. 18) [281]. Conversely, mice with ablated monocytes/macrophages exhibited an intensified stress response in the brain, as evidenced by elevated plasma corticosterone levels and a neuroinflammatory phenotype. Flow cytometry of noise-exposed murine brains revealed a significant increase in activation markers for microglia - CD68, CD86, and major histocompatibility complex class II (MHC-II). These markers, however, did not return to baseline even with the genetic ablation of LysM^+^ cells, further substantiating the conclusion that microglia are LysM-negative (illustrated in Fig. 18) [281]. This intriguing contrast implies a potential impact of noise on the blood-brain barrier, a phenomenon also reported in hypertension [466]. Additionally, the presence of a pro-oxidative and pro-inflammatory environment seemed to influence the activation state of astrocytes within the brains of noise-exposed mice.

Loud noise has also been found to induce systemic inflammation (e.g., in the skeletal muscles) [467]. High levels of noise (100 dB in mice and 120 dB in rats) were shown to activate SHC-transforming protein 1 (SHC1, p66^Shc^), a mitochondrial source of H_2_O_2_, which was associated with higher levels of markers of oxidative stress, inflammation (vascular endothelial growth factor (VEGF), interferon γ (IFN-γ) and IL-1α were upregulated; IL-10 and ciliary neurotrophic factor (CNTF) were downregulated), and ischemia in the cochlea, all of which were prevented by *Shc1* deletion [395]. Moreover, numerous pro-inflammatory cytokines and chemokines were found to be upregulated in an array analysis, which nicely correlates with the central role of inflammation for noise-induced hearing loss [468,469].

Cross-sectional studies have indicated that exposure to traffic noise may lead to elevated levels of IL-12 (a myeloid cytokine) and high-sensitivity C-reactive protein (hsCRP) coupled with reductions in natural killer cell populations and activity [460,470]. However, results are not consistent [220,471]. The SAPALDIA study noted that DNA methylation was enriched in pathways corresponding to inflammation, cellular development, and immune responses following prolonged exposure to source-specific transportation noise and air pollution [459]. A study from Germany found that extended exposure to nighttime traffic noise was associated with subclinical atherosclerosis, particularly in individuals displaying early arterial calcification [472,473]. These findings indicate a potential link between amplified recruitment and/or activation of immune cells by noise and compromised cardiovascular function.

A human study that leveraged clinical ^18^F-fluorodeoxyglucose positron emission tomography-computed tomography (PET–CT) imaging in 498 individuals without active malignancy or clinical CVD offered additional insights into the immune consequences of noise exposure that contribute to CVD [47]. This study extended prior work showing that a neuroimmune pathway involving heightened stress-associated neural activity (as amygdala metabolic activity relative to regulatory cortical activity) linked chronic stress and socioeconomic stressors to CVD to show that noise exposure was also an important and independent driver of this pathway [49,50]. Notably, increased noise exposure associated with heightened metabolic activity of the amygdala (relative to regulatory cortical activity), arterial inflammation, and a greater risk of MACE (HR 1.341, 95 % CI 1.147–1.567, per 5 dB(A) increase). These associations remained robust even after multivariable adjustments for potential confounders including air pollution, socioeconomic status, and CVD risk factors. Further investigation via mediation analysis showed a sequential mechanism through which elevated noise exposure was associated with MACE, involving heightened amygdala activity and arterial inflammation [47,48]. Of note, an additive impact of exposure to increased noise and air pollution on arterial inflammation and MACE risk was reported that seems to synergize at the level of the arteries (noise enters the brain while air pollution activates leukopoietic tissues) [474]. Moreover, stress-associated neural activity has been further linked to atherosclerosis in several separate cohorts by showing a relationship with coronary artery disease complexity, non-calcified coronary plaque burden, and coronary fat attenuation index and a greater risk for recurrent stroke in patients with prior stroke [[475], [476], [477], [478]]. Collectively, these findings identify an important pathway that contributes to the development of CVD as a result of noise exposure.

#### 2.3.3 Circadian clock

**Fig. 19:**
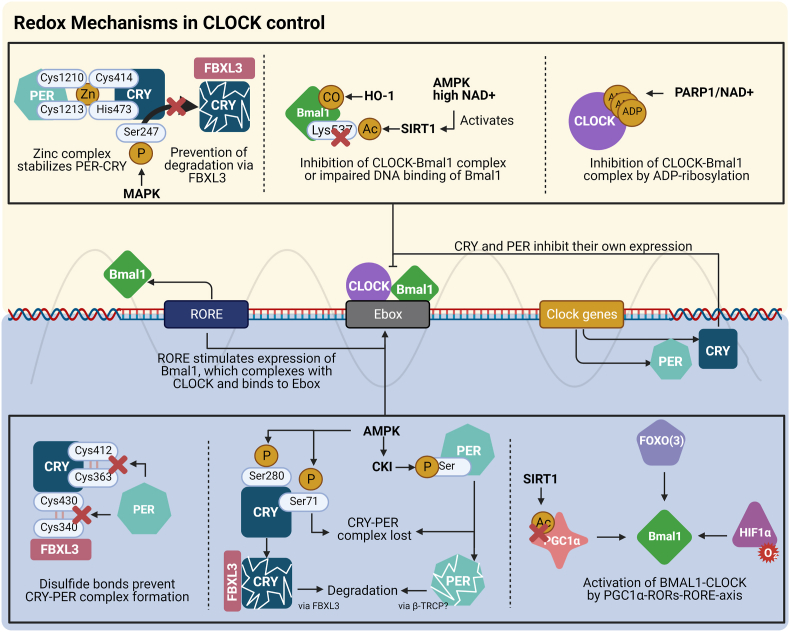
**Proposed mechanisms of redox regulation of the circadian clock.** The circadian clock is affected by various redox-sensitive processes that ultimately lead to repression (top, yellow) or activation (bottom, grey) of the central transcription factor complex BMAL1/CLOCK. Redox-sensitive cysteine thiol groups (C363 and C412, *bottom left*) and a zinc-sulfur center (C1210 and C1213 of PER2, C414 and H473 of CRY1, *top left*) were identified in mammalian CRY1 and PER2 that act as redox switches (via disulfide bond formation) controlling CRY-PER interactions and thereby the activity of the CLOCK/BMAL1 complex [487,495,496]. The scheme also contains other redox-sensitive pathways in the regulation of circadian rhythm such as redox-sensitive kinases AMP-activated protein kinase (AMPK) or mitogen-activated protein kinase (MAPK). AMPK phosphorylates S71 and S280 to affect the affinity of CRY1 for the E3 ligase FBXL3 and thereby CRY stability [497,498]. AMPK via casein kinase I (CKI) phosphorylates PER to cause proteasomal degradation via β-transducin repeat-containing protein (β-TRCP) [499,500] (*bottom middle*). The MAPK phosphorylates S247 to affect CRY-dependent transcriptional repression of BMAL1/CLOCK [497,501] (*top left*). Furthermore, stress-response proteins such as poly (ADP-ribose) polymerase (PARP-1) [500,502] (*top right*), HO-1 (*top middle*), hypoxia-inducible factor 1α (HIF-1α) [503], peroxisome proliferator-activated receptor gamma coactivator 1α (PGC-1α) [499], FOXO3 [504,505] (*bottom right*) and the histone deacetylase sirtuin 1 (SIRT-1) [499,506,507] (*bottom right and top middle*) affect the circadian clock by modifying the transcriptional activity of BMAL1/CLOCK. Importantly, the expression of several antioxidant and O_2_^•−^ and H_2_O_2_-producing enzymes is controlled by the circadian clock and thereby contribute to cellular redox homeostasis [508,509]. Summarized from the respective references in this legend using BioRender.com. Reused from Ref. [489] with permission.

The circadian clock regulates crucial biological functions like sleep, body temperature, appetite, and cognitive processes. It operates cyclically (over the day) releasing hormones, most notably cortisol, melatonin, ACTH, testosterone, renin, aldosterone, angiotensin, and catecholamines [479]. Disruption to this circadian rhythm can be induced by high (nighttime) noise exposure burden [13,136,200] or sleep pattern disturbances like those in shift workers [[480], [481], [482]]. It is a suspected risk factor for many diseases, including CVD, breast cancer [483,484], and psychiatric disorders [485,486]. The circadian rhythm is generally under redox control; direct redox modifications of circadian components cryptochrome (CRY), period (PER), and F-box/leucine-rich-repeat protein 3 (FBXL3) involve thiol oxidation/reduction and the formation or disruption of zinc-sulfur complexes. Maintenance of the appropriate oxidative status allows the circadian rhythm to govern the proper binding of these components to the essential regulators of circadian control—circadian locomotor output cycles protein kaput (CLOCK) and brain and muscle Arnt-like protein 1 (BMAL1) complex, as depicted in Fig. 19 [487]. This presents a point of intersection between the effects of noise and regular cellular timekeeping – if noise can efficiently disrupt the oxidative balance, it can potentially disturb the circadian rhythm. Redox-sensitive kinases, histone deacetylases, stress-response proteins, and transcription factors can be influenced by ROS, thereby impacting the clock system [207,251]. On the other side it is the nature of noise, which can disrupt sleep and interfere directly with timekeeping [488]. A comprehensive overview of the impact of various environmental stressors, including mental/social isolation stress, air pollution, heavy metals, and pesticides, on the circadian clock and its adverse redox regulation has previously been published [381].

Only a few studies have explicitly addressed noise effects on the circadian rhythm. However, other environmental cues and stressors of the cyclical regulation [381,489] (e.g., light, food intake, and temperature), have been found to interfere with the circadian rhythm. A study in mice subjected to continuous aircraft noise exposure for 4 days (72 dB(A)) showed downregulation of *Per1* and *REV-ERB-α/β (Nr1d1/2)* or *RORα*, along with the upregulation of *Bmal1, Cry1, Cul1, Prkag1/2,* poly (ADP-ribose) polymerase (*Parp1*)— in total, more than 30 circadian genes displayed altered expression levels in the aorta and kidney compared to unexposed controls [16]. Additionally, the downregulation of forkhead-box-protein O3 (*FoxO3*), a central transcription factor regulating circadian genes in vascular tissue, was noted. Pharmacological activation of FoxO3 using bepridil successfully countered noise-induced oxidative stress in the aorta and the resulting endothelial dysfunction [16]. In a separate study examining the transcriptomics of neurons within the inferior colliculus, a brain structure vital for sound processing, distinct profiles between day- and nighttime exposure appeared in clock genes [490]. Furthermore, a phase shift was reported for corticosterone levels in feces of noise-exposed mice, indicating a dysregulated circadian rhythm [491], which may differ in different mouse strains [492]. Noise exposure alters clock gene expression (*Per1, Per2, Bmal1,* and *Rev-Erbα*) in the cochlea and the inferior colliculus, having direct implications for noise-induced hearing loss, but may also be relevant for dysregulated circadian rhythms in other brain regions and remote organs [493]. A differential effect of daytime versus nighttime noise exposure on several inflammatory cytokines with higher peak levels after daytime noise has also been observed [494].

#### 2.3.4 Noise-induced changes of epigenetic pathways

Gene expression is critically and dynamically controlled by epigenetic changes determining the physiological response to environmental factors. As such, it is no surprise that many such epigenetic alterations have been identified in the development and progression of atherosclerosis and correlate with its severity [510,511]. Importantly, epigenetic changes to the genome are generally redox-regulated [[512], [513], [514]]. For this reason, it is speculated that noise-induced oxidative imbalance could dysregulate the landscape of gene expression via interruption of these epigenetic regulations. The methylome of CVD and risk is currently an important research topic [515], which carries over into the noise field. Changes in overall methylation were reported in the brains of noise-exposed rats, demonstrating that noise exposure can interfere with transcriptional signals [516]. The Swiss SAPALDIA cohort study, based on 1389 participants, reported noise-induced alterations of DNA methylation patterns indicating inflammatory activation and immune response [459]. Downstream of epigenetic changes, studies have reported alterations in coding gene expression in murine aorta, heart, and kidney in response to noise, as detected by RNA sequencing [14,16]. Studies on hearing loss have reported similar results [517,518]. Noncoding RNA and microRNA expression have also been reported to change in response to noise, an important caveat when considering that these are increasingly recognized as playing a role in health and disease [519,520]. For example, increased expression of miR-134/183 occurred in the central amygdala following acute stress exposure [521], both of which have been reported to be increased in patients with coronary artery disease and depression. Numerous microRNAs, which regulate/respond to antioxidant defense or pro-oxidative proteins, are reported to be affected by environmental exposures [519,520]. Epigenetic effects observed in human and animal studies on hearing loss and in non-auditory models have also been reviewed [522].

A new emerging concept promotes early life (fetal) reprogramming by various exposures to explain the large impact of environmental exposures on disease development in later life [[523], [524], [525], [526]]. For example, dietary factors during pregnancy, such as overnutrition or malnutrition, severely affect the risk of the offspring developing a metabolic disease or CVD during later life [[527], [528], [529]]. This also holds true for noise pollution, which affects the risk of disease in human and animal offspring via prenatal epigenetic reprogramming [530].

### 2.4 Quality assessment of applied methods for detection of reactive oxygen and nitrogen species and associated oxidative damage

#### 2.4.1 Redox biomarkers reported for noise exposure

The postulated key role of ROS formation for noise-induced pathophysiology is supported by a broad range of oxidative stress markers and read-outs detected in noise-exposed animals (Fig. 17). Classical markers comprise 3-nitrotyrosine-, malondialdehyde- or 4-hydroxynonenal-positive proteins, 8-isoprostane as well as 8-hydroxy-(deoxy)guanosine (8-OH-(d)G) in different tissues and plasma/serum (reviewed in Refs. [45,531]). In addition, eNOS S-glutathionylation and uncoupling of nNOS were found in noise-exposed mice. These indirect oxidative stress markers were accompanied by direct measurement of O_2_^•−^ formation by high-performance liquid chromatography (HPLC)-based quantification of 2-hydroxyethidium and of H_2_O_2_ and peroxynitrite by various other fluorescence staining/chemiluminescence-based techniques. Important redox biomarkers are listed and scored according to their reliability and usefulness in biological samples in Table 3. Most of these biomarkers were also described for various CVD conditions [345,532], neurodegenerative disease [42,346], metabolic disorders [39,40], and different forms of cancers [41,347]. When looking at the table, it becomes evident that redox biomarkers were not measured frequently in noise-exposed human subjects, which warrants future efforts into this direction.

**Table 3:** Redox biomarkers reported in non-auditory noise exposure studies focusing on the circulation and brain regions or other remote organs.

Redox biomarker	Method of detection/quantification^a^	Quality score^b^	Ref.
3-Nitrotyrosine	Plasma and cardiac levels of 3-NT-positive proteins by dot blot analysis – also abolished in *Nox2* knockout mice and only visible upon sleep phase noise. Additive increase by noise/AT-II infusion and suppression by HO-1/NRF-2 activation by hemin and DMF. Aortic levels of 3-NT-positive proteins by IHC with additive increase by noise/AT-II infusion. 3-NT levels increased in plasma of noise-exposed humans.	++	Plasma and heart [14,16,245,247,461]; Aorta [14,247,281,463]; Humans [16]
Malondialdehyde	Plasma and cardiac levels of MDA-positive proteins by dot blot analysis – also abolished in *Nox2* knockout mice and only visible upon sleep phase noise and prevention by LysM^+^ cell ablation. Brain MDA levels increased in rats by noise by ELISA or by thiobarbituric acid assay.	+	Plasma [14,16,281]; Heart [245]; Rats [206]; Brain [571,574]
4-Hydroxynonenal	Aortic levels of 4-HNE-positive proteins by IHC. Plasma levels of 4-HNE-positive proteins by dot blot analysis - diminished by HO-1/NRF-2 activation by hemin and DMF.	++	Aorta [14,463]; Plasma [247,461]
8-Hydroxy-(deoxy)guanosine	Aortic levels of 8-OH-(d)G-positive DNA/RNA by IHC.	++	Aorta [463]
Lipid peroxides and lipids	8-isoprostane levels increased in plasma of noise-exposed humans by ELISA. Decreased unsaturated fatty acids in plasma of mice by LC/MS analysis. Increased plasma protein carbonyls in rats after noise by DNPH derivatization and Western blot.	+++	Plasma humans [16]; Plasma mice [363] and rats [205,574]; Brain rats [275]
eNOS S-glutathionylation and uncoupling	Aortic and cardiac eNOS S-glutathionylation by immunoprecipitation and Western blot analysis. Aortic eNOS uncoupling by endothelial DHE staining with or without l-NAME. Endothelial ROS (most likely O_2_^•−^) formation and eNOS S-glutathionylation higher in sleep phase versus awake phase noise exposure and additive increase by noise/AT-II infusion and prevention by LysM^+^ cell ablation. Acute vitamin administration improved impaired endothelial function measured by FMD in humans.	++	Aorta and heart [14,16,247,281]; Human [30,234]
nNOS uncoupling	Uncoupling of nNOS by phosphorylation of serine 847 b y Western blot analysis. Also cerebral ROS (most likely O_2_^•−^) detection by DHE staining was normalized by l-NAME or specific nNOS inhibitor ARL-17477.	+	Brain [16]
ROS detection by DHE staining	Aortic and cerebral ROS (most likely O_2_^•−^) formation by DHE staining. Signal lower in *Nox2* knockout mice and additive increase by noise/AT-II infusion and prevention by LysM^+^ cell ablation. Cerebral mtROS (O_2_^•−^ and H_2_O_2_) formation by mitochondria-targeted DHE (mitoSOX) staining increased by noise and not diminished in *Nox2* knockout mice. Aortic and cerebral ROS (O_2_^•−^ and H_2_O_2_) levels higher in sleep phase versus awake phase noise exposure – in brain diminished by NOX-2 inhibitor GSK2795039. Cerebral mtROS (O_2_^•−^ and H_2_O_2_) formation by mitoSOX staining higher in sleep phase versus awake phase noise exposure. Aortic, cardiac and cerebral ROS (O_2_^•−^ and H_2_O_2_) signals diminished by HO-1/NRF-2 activation by hemin and DMF. Retinal and mesenteric ROS (O_2_^•−^ and H_2_O_2_) formation by DHE staining - prevention by LysM^+^ cell ablation.	+	Aorta and brain [15,16,245,247,248,281,461]; Heart [461]; Retinal/mesenteric microvessels [281]
Superoxide detection by HPLC	Aortic and cerebral superoxide formation by HPLC-based quantification of 2-hydroxyethidium. Signal lower in *Nox2* knockout mice by trend (aorta) and significant (brain/cortex). Aortic and cerebral superoxide levels higher in sleep phase versus awake phase noise exposure. Aortic and cardiac mitochondrial superoxide formation by HPLC-based quantification of 2-hydroxyethidium and mito-HE. Additive increase by noise/AT-II infusion and prevention by LysM^+^ cell ablation and suppression by exercise, fasting and AICAR (AMPK activator).	+++	Aorta and brain [16,245,567]; Aorta and cardiac mitochondria [245,247,248,281,567]
Oxidative burst	Whole blood leukocyte-dependent ROS (O_2_^•−^ and H_2_O_2_) formation upon stimulation with phorbol ester dibutyrate or zymosan A by the luminol analog L-012-enhanced chemiluminescence. Signal abolished in *Nox2* knockout mice and was aggravated in Ogg1 knockout mice with impaired repair capacity of 8-OH-(d)G lesions. Cell-specific ROS (O_2_^•−^ and H_2_O_2_) by flow cytometry in different myelomonocytic cell subsets.	+	Blood [16,245,248,463]
NOX activation	Cardiac NOX activity by NADPH-dependent stimulation in membrane fractions using lucigenin-enhanced chemiluminescence. Phosphorylation of p47^phox^ at serine 328 and of myristoylated alanine-rich C-kinase substrate (MARCKS) (also in lung), the substrate of protein kinase C, by Western blot analysis.	–	Heart [14]; Brain [16]; Lung [15]
Upregulation of NOX isoforms	Aortic, pulmonary and myelomonocytic NOX-2 upregulation by Western blot analysis and immunohistochemistry (IHC) or *Nox2* mRNA by RT-PCR - prevention by LysM^+^ cell ablation. Upregulation of *Nox1* mRNA in lung endothelial cells and of NOX-1 protein by trend in the lung. Cerebral upregulation of *Nox1* mRNA only by sleep phase noise.	+	Aorta and LEC [14,245,247,281,463]; Brain [16]; Lung [15]; Aorta and PBMCs [248]
*Nox2* deletion	Vascular functional impairment, inflammatory phenotype and metabolic parameters in response to noise normalized by the knockout.	++	[16,363]
*Hmox1* upregulation	Upregulation of *Hmox1* mRNA in lung endothelial cells. Upregulation of *Hmox1* mRNA and HO-1 protein by trend in the heart but in contrast decreased bilirubin levels (by oxidative break-down?).	+	LEC [14]; Heart [461]
Diminished aortic ^•^NO formation	Aortic ^•^NO bioavailability was decreased as measured by electron paramagnetic resonance spectroscopy-based Fe(DETC)_2_ spin trapping. DETC means diethyldithiocarbamate.	+	Aorta [14]
Altered plasma nitrite levels	Plasma nitrite/nitrate increased by noise as measured by commercial kit (due to iNOS induction). Plasma nitrite diminished by trend in mice by noise as measured by HPLC-based assay - additive significant decrease by noise/AT-II infusion.	+	Rats [206]; Mice [245,247]
Altered levels of SODs	Plasma and cerebral SOD activity diminished by noise in rats by ELISA or nitroblue tetrazolium assay. Cardiac SOD2 expression diminished by Western blot analysis in mice.	+	Plasma [206,571,574]; Heart [247]
Downregulation of glutathione peroxidase 1 (GPx-1) and catalase and others	Downregulation of various antioxidant genes in aorta by RNA sequencing. Diminished expression of catalase mRNA in brain was abolished in *Nox2* knockout mice. Cerebral downregulation of catalase mRNA only by sleep phase noise. Plasma GPx-1 upregulation by ELISA in workers with high noise exposure. Cerebral GPx activity diminished by commercial kit.	+	Aorta [14]; Brain [16,571]; Human plasma [227]
GSH levels	GSH levels decreased by noise.	++	Rats [275]
DNA damage	Blood DNA strand breaks by comet assay higher in workers with high noise exposure. peripheral blood mononuclear cell (PBMCs) 8-oxoguanine glycosylase (OGG-1)-sensitive DNA strand breaks by comet assay higher in noise-exposed humans. Serum DNA damage in rats by ELISA. Heart and adrenal gland DNA strand breaks by comet assay higher in rats after loud noise.	+	Human blood [227]; Human PBMCs [333]; Rat [380,605,606]
Decrease in ΔΨ_m_	Impaired mitochondrial membrane potential by TMRM in noise-induced hearing loss.	–	Cochlea [607]

a If not stated redox biomarkers were detected in noise-exposed mice.

b Quality scores, based on the recommended detection modalities (see 2.4.2.): +++, highly recommended/state-of-the-art; ++, recommended; +, of potential value, may lack the specificity and/or straightforward interpretation; –, not recommended if used alone.

#### 2.4.2 Quality of the noise-relevant redox markers

Rigorous application of the assays used to assess the extent of oxidative stress/oxidative damage and meaningful interpretation of the experimental results require a knowledge of the principles of the assays used, their limitations and the factors controlling the detected signal intensity. Below, is a short description of the assays used to assess noise-induced oxidative stress, with the major limitations identified and the recommendations of the experimental approaches to be used.

##### 2.4.2.1 ROS detection

Oxidative stress is described as an imbalance between the oxidant (ROS) production and scavenging, resulting in increased steady-state levels of the oxidants with concomitant increased oxidative modification of biomolecules. Therefore, the detection of ROS, e.g. O_2_^•−^ and H_2_O_2_, is one of the most direct assays for oxidative stress. As ROS is an umbrella term for multiple chemical species of different chemical reactivities and biological roles, whenever possible, the identity of the detected ROS should be established, and different chemical probes and assays must be applied to different types of ROS [533,534]. It should be emphasized that all ROS are short-lived in the biological setting, and any detection attempt requires the application of the appropriate probe at the time of ROS production. Measurement of ROS using appropriate redox probes can be carried out in a wide range of experimental models, from enzymatic assays, cellular organelles, cultured cells *in vitro*, and isolated tissues to live animals [427,430,535]. The analytical methods for the most commonly measured ROS, specifically O_2_^•−^ and H_2_O_2_, are described in section 2.2.4 just after description of the sources of O_2_^•−^ and H_2_O_2_ being active in noise exposure conditions.

##### 2.4.2.2 Ex vivo determination of the expression and activity of enzymatic ROS generating and scavenging systems

An experimentally more straightforward but less direct approach to estimate the position of the redox balance *in vivo* is to measure ex vivo the expression and activity of the enzymes, which are known to be involved in ROS generation and/or metabolism. Among the major sources of ROS that may contribute to oxidative damage are NADPH oxidases (source of O_2_^•−^ and H_2_O_2_), mitochondrial electron transport chain (source of O_2_^•−^ and H_2_O_2_), xanthine oxidase (source of O_2_^•−^ and by dismutation also H_2_O_2_), MAO (source of H_2_O_2_), nitric oxide synthases (source of O_2_^•−^, H_2_O_2_ and peroxynitrite), and myeloperoxidase (source of HOCl). Among the ROS detoxifying/metabolizing enzymes, the most assayed are superoxide dismutases, and others specialized in the degradation of H_2_O_2_ (or in some cases other peroxides) such as catalase, glutathione peroxidases, and the components of peroxiredoxins/thioredoxins pathway. The assays applied may involve the determination of the enzyme expression at the transcriptional and/or protein level and establishing the status of their posttranslational modification, known to affect the enzymatic activity (e.g., phosphorylation of NADPH oxidase 2 complex assembly components, glutathionylation and phosphorylation of NOS enzymes, acetylation of mitochondrial superoxide dismutase (SOD2)), and bioavailability of cofactors (e.g., BH_4_ for NOS enzymes). However, monitoring the enzymatic activity is the preferred approach, and intact pieces of tissues (e.g., blood vessels), tissue homogenates, isolated organelles (e.g., mitochondria), or membranes have been used to assess such activity. It is essential to supply the enzymes with appropriate substrates for constant activity over the incubation/measurement period and to use specific inhibitors to confirm/establish the identity of the enzyme. Typically, a kinetic assay to determine the reaction rate is preferred, as opposed to an end-point measurement.

##### 2.4.2.3 Biomarkers of oxidative stress

Separate from ROS measurements and enzymatic activity assessment, another important experimental approach is to measure products of modification of biomolecules by cellular oxidants [345,536,537]. This is typically associated with (but not limited to) oxidative damage to cell components. The major advantage of using of oxidative stress biomarkers is the assessment of endogenous products of the action of ROS and no need to apply any chemical probe to the model used. This opens a potential for non-invasive assessment of such biomarkers in body fluids, allowing a straightforward expansion of such studies to humans. The four major biomolecule classes known to be affected by cellular ROS are small molecule antioxidants (e.g., reduced glutathione (GSH)), lipids, proteins, and DNA.

**Glutathione and ascorbate oxidation.** Measurement of GSH and/or GSH/glutathione disulfide (GSSG) ratio has been widely used to assess the occurrence of redox stress in tissues. Both enzymatic assays and GSH detection using fluorescence probes or HPLC/liquid chromatography-mass spectrometry (LC-MS)-based analyses were reported [538]. Chromatographic techniques offer high selectivity and sensitivity and are preferred [539]. Care should be taken to avoid GSH degradation/modification during sample storage and processing. Conversion of another small molecule antioxidant, ascorbate, to its oxidation products, ascorbyl radical and/or dehydroascorbic acid, has also been utilized to assess oxidative stress *in vivo* [540,541].

**Lipid peroxidation products.** Analysis of the end products of lipid peroxidation is commonly applied to assess the extent of oxidative stress *in vivo* [542,543]. At the same time, enzymatic lipid peroxidation is catalyzed, e.g., by lipoxygenases. The most commonly detected products of lipid peroxidation include malondialdehyde (MDA), 4-hydroxynonenal (4-HNE) and isoprostanes [544]. Various detection methods have been applied, but their detection by LC-MS-based techniques is recommended as highly specific, resulting in the highest confidence in signal assignment to any specific product among the techniques used [545]. For determination of the extent of chemical lipid peroxidation, a specific product of oxidation of arachidonic acid, 8-iso-prostaglandin F_2α_ (8-iso-PGF_2α_), has been proposed as the most reliable biomarker and has been extensively used [546]. The possibility of forming the same product in enzymatic reaction catalyzed by prostaglandin-endoperoxide synthases (PGHS) led to the proposal to profile different oxidation products of arachidonic acid and use the 8-iso-PGF_2α_/PGF_2α_ ratio for the determination of the relative contribution of chemical and enzymatic pathways to the total detected pool of 8-iso-PGF_2α_ [547]. It should be emphasized that LC-MS-based analyses are recommended and enable profiling of the different lipid peroxidation products in a single run.

**Post-translational modification of proteins.** Many amino acid residues in proteins are prone to oxidative modification, which may form relatively stable and specific end products of potential value as biomarkers of oxidative stress [548,549]. Among the most common modifications serving such a purpose are newly formed protein carbonyls, tyrosine nitration (a marker of peroxynitrite and/or myeloperoxidase/NO_2_^−^/H_2_O_2_) and chlorination (a marker of HOCl), formation of dityrosine links and protein hydroperoxides (markers of one-electron oxidizing species), protein glutathionylation and oxidation of thiols to sulfenic, sulfinic and sulfonic acids (markers of thiol oxidizing agents, including H_2_O_2_, ONOO^−^, and HOCl), oxidation of methionine to methionine sulfoxide (a marker of H_2_O_2_, HOCl, one-electron oxidants), and formation of protein carbonyls (a general marker of protein amino acid oxidation). It should be noted that some modifications listed may also be formed in enzymatic systems, including cysteine and methionine residues oxidation [550]. Protein glutathionylation may result from the reaction of GSH with oxidized/nitrosated protein thiols or vice versa. Protein carbonyls may be formed due to the reaction of proteins with the electrophilic products of lipid peroxidation, including 4-HNE or protein glycation. Many modifications mentioned may be detected using specific antibodies, either by immunoblotting or via ELISA. In specific cases, probes for specific modification can also be used (e.g., dinitrophenylhydrazine, DNPH, for protein carbonyls). Still, LC-MS-based detection and quantification is recommended as it offers the most rigorous analysis of the modification type and detection of multiple modifications in a single protein [548]. The combination of chemical labeling of specific modification sites with standard enrichment methods (e.g., antibody-, biotin-, click chemistry-based) enables high confidence in the identification of the proteins modified and analyses of the type(s) and site(s) of modification. Due to the possibility of intramolecular electron/charge transfer, the site of the detected protein modification may differ from the site of initial interaction with the oxidant.

**Nucleic acid oxidation.** The intracellular oxidizing environment may also result in oxidative modification of the nucleic acids, DNA and RNA [551]. While the comet assay widely monitors cellular DNA damage, it lacks specificity to cellular oxidants. Measurement of the extent of conversion of 2’-deoxyguanosine to 8-hydroxy-2’-deoxyguanosine (8OHdG) is the most widely accepted experimental approach to monitor DNA oxidation [552]. The measurement requires DNA isolation and digestion, followed by determining 8OHdG, either by ELISA or LC-MS/MS. Based on the multi-laboratory assay comparison, ELISA-based quantification of 8OHdG is discouraged, while mass-spectrometric analyses are recommended [553].

**Induction of cellular antioxidant response.** Upon exposure to oxidants or electrophiles, the cell may adapt by boosting its potential to detoxify such species, for example, by increased expression of antioxidant enzymes. One of the pathways linking oxidative/electrophilic stress to the abovementioned adaptive response includes NRF-2 protein and its nuclear target, antioxidant/electrophile response element (ARE/EpRE) [554]. Therefore, markers of ARE activation, including the expression of the downstream protein targets at the gene and protein level, have been used as markers of oxidative stress [555]. It should be noted, however, that such an adaptive response may result in the resolution of oxidative stress. Thus, the accurate interpretation of the data may be difficult. Furthermore, plasma levels of cellular antioxidants (both small molecule and enzymatic) may result from the damage of specific tissues, not necessarily related to oxidative stress.

Besides the induction of direct antioxidative defense mechanisms, such as superoxide dismutases or glutathione system enzymes, oxidative stress is often accompanied by an induction of various repair systems. This includes the systems responsible for the detoxification of harmful intermediate oxidation product (e.g., 4-HNE) [556,557], or degradation and repair systems for damaged macromolecules. In particular the components of the proteasomal system are under the control of the Nrf2 system [558] or are induced under oxidative stress/inflammatory conditions [559]. However, there are no systematic studies on the role of these repair systems under noise conditions in the cardiovascular system. Proteotoxic stress was investigated in the cochlear cells [560,561], also interesting due to the existence of extremely long-living proteins in the cochlear. Therefore, the impact of noise on antioxidative repair systems is still an open question.

##### 2.4.2.4 Additional considerations

**Probe availability/biodistribution.** Under most conditions, redox probes can intercept only a fraction of the pool of any given oxidant due to the competition with intra-/extracellular targets/scavengers of the oxidant. Therefore, the probe's tissue level is one factor controlling the amount of the oxidant intercepted and, thus of the detectable product formed. The bioavailability of the probe should be experimentally verified, and measured for each sample due to the possible differences between the treatment groups and the variations between individual animals. Some probes, including DHE, are rapidly oxidized in the blood due to high reactivity towards heme proteins, and site/tissue-specific probe administration by direct injection may be a preferred approach. Expressing the results as the ratio of the product formed to the detected probe level may help address the differences in probe availability. Similarly, the concentration of various biomolecules being oxidatively modified should be considered when assessing the biomarkers of oxidative stress, as those may be modulated by the diet used and changes in metabolism. This may be reflected in raised levels of the biomarkers, even when the level of oxidants remains unchanged. Again, “normalizing” the data to the level of the biomolecules undergoing oxidative modification may be used to address such variability.

**Metabolism and biodistribution of probe-derived products and biomarkers.** The concentration of any analyte (redox probe, the product formed, any biomarker) at any given time in a specific site/tissue is a product of the rate of its formation and/or uptake and the rate of its degradation/efflux. Therefore, the potential pathways of the loss of the analyte should be considered, as their modulation may be misinterpreted as a change in the rate of production of the analyte of interest. For example, decreased activity of the proteasomal system may result in increased accumulation of the post-translationally modified proteins.

**Determination of oxidative stress in humans.** Given the large variety of oxidants formed, considering the various locations of formation and the various kinetics of reactions and transportation of oxidized products into the circulation it is widely accepted that in an ideal setting a set of different parameters should be determined to get a clear result about oxidative processes [345,[562], [563], [564]]. This avoids also the influence of some non-oxidative pathways on the results.

### 2.5 Antioxidant interventions

Various studies have provided molecular support for the beneficial effects of antioxidant interventions against noise-induced damage, including a reduction of systemic oxidative stress (e.g., aortic superoxide formation as measured by HPLC analysis of 2-hydroxyethidium) by genetic deletion of the *Nox2* gene (*Nox2*^*−/−*^) or pharmacological inhibition (GSK2795039) of the NOX-2 protein [16]. Noise-exposed *Nox2* knockout mice also had normal endothelial function. Impaired FOXO3 signaling is likely a key mechanism in animal models with low-level noise exposure [245] since the activation of FOXO3 by the calcium antagonist bepridil significantly improved several vital parameters, such as endothelial dysfunction and vascular/cerebral oxidative stress [16]. The adverse effects of noise, including hypertension, endothelial dysfunction, vascular and cerebral oxidative stress, and markers of inflammation were also prevented by induction of the antioxidant principle NRF-2 with dimethyl fumarate or direct stimulation of the antioxidant defense enzyme HO-1 by hemin [461]. Both drugs substantially increased HO-1 and the potent antioxidant bilirubin in noise-exposed mice as a potential mechanistic explanation of NRF-2-mediated protection. Studies have also found protective effects of NRF-2 activators against mental stress conditions [565], reflected by the beneficial action of CDDO-imidazole in a model of noise-induced hearing loss [566]. Of great interest are non-pharmacological mitigation strategies against noise-induced damage (e.g., physical exercise and intermittent fasting), conferring potent antioxidant and anti-inflammatory effects largely mediated by AMPK as shown for noise-exposed mice [567]. Of note, there is an important connection between NRF-2 and AMPK as the kinase phosphorylates NRF-2 and thereby causes activation of the transcription factor [568], also with high relevance for the protective effects of physical exercise [569] and intermittent fasting [570].

N-acetylcysteine therapy prevented oxidative stress envisaged by lipid peroxides in the brain, depressive phenotype, and anxiety-like behavior in mice exposed to loud noise [571]. Another study reported that changes in the neurotransmitters noradrenaline and serotonin, lipid peroxides, and antioxidant defense enzyme activities in the brain of rats exposed to loud noise were mostly mitigated by vitamin E treatment [572]. Also, the neuroprotective effects of sildenafil were observed in mice exposed to severe noise stress, which were characterized by protection against oxidative stress and memory dysfunction [573]. Rosuvastatin normalized oxidative stress plasma markers in response to loud noise in rats [574]. Summaries of antioxidant interventions against noise-mediated oxidative damage in brain tissues have previously been reported [531,575]. In addition, numerous antioxidant interventions were reported in models of noise-induced cochlear damage and hearing loss (reviewed in Ref. [67]), where the NOX-3 isoform seems to play a predominant role for ROS formation and oxidative damage [576,577].

### 2.6 Applying the oxidative stress concept to broader mental stress conditions

Transportation noise can act as a psychological (mental) stressor similar to other mental stressor, e.g., job-strain. Epidemiological studies have indicated that transportation noise may increase risk of anxiety and depression, though high-quality prospective studies on this are still needed [578]. Accordingly, the oxidative stress concept for noise exposure should be discussed in a broader context for all psychological stress conditions to provide a more general perspective applicable to different medical fields.

Psychosocial stress is a complex entity, comprised of many factors that can produce an emotionally- and physically-complicated response. Despite this, a series of prospective epidemiological studies have identified two components of this stressor that appear to have an outsized weight in stressful work environments – job strain and effort-reward imbalance. Job strain describes a working situation with high demand and pressure to perform but low control over the task. Employees in these circumstances have higher cardiovascular risk. Another work-related stressor that puts workers at elevated cardiovascular risk is effort-reward imbalance, where individuals expend high effort to achieve rewards (salary, promotion, recognition, security). Examples of occupational groups that suffer significantly from the two scenarios of work stress are nurses and teachers [579]. Results from >20 cohort studies demonstrate a 1.4-fold increased risk of coronary heart disease for individuals in high-vs. low-stress work [580,581], even after multivariable adjustment for other cardiovascular risks. Accordingly, mental stress is strongly associated with cardiovascular risk and other disease entities, which also holds true for interactions between road traffic and occupational noise exposure, as well as job-strain, in relation to the risk of myocardial infarction [582]. To bolster the epidemiological claims, these studies included measurement of job strain or effort-reward imbalance and reported associations with elevated autonomic nervous system activity indicators - plasma cortisol and noradrenaline, blood pressure, heart rate, and heart rate variability [583]. Enhanced autonomic nervous system activity, higher markers of oxidative stress in blood or vascular tissue and increased NFκB and pro-inflammatory activity were also documented [584]. Urinary 8OHdG and H_2_O_2_, two oxidative stress biomarkers, showed associations in human job stress settings [585], and anticipatory cortisol reactivity [586]. This was accompanied by an elevation of NOX-2 in the hypothalamus, mirroring the rat model of psychosocial stress [587]. These data indicate that oxidative stress represents a major pathomechanism initiated by mental stress.

Concluding with the notion that oxidative stress is a well-recognized trigger/promoter of cardiovascular disease provides a rational pathomechanism for higher cardiovascular risk observed under mental stress conditions. There is high-quality research investigating mental stress as a cardiovascular risk in animal studies [532] and preliminary evidence in studies on humans [345]. For example, H_2_O_2_-induced vasodilation was impaired in congestive heart disease due to switching potassium channels [588]. Clinical cardiovascular risk factors are also correlated in numerous CVD epidemiology studies. The current state of knowledge on CVD and oxidative stress has been recently reviewed [589,590], focusing on the major enzymatic sources of oxidants, NADPH oxidases, mitochondria, xanthine oxidase, lipoxygenase, and myeloperoxidase. While the connections have been forged, further research is challenged by the relative difficulty of closely monitoring the oxidative state in human subjects with job strain and psychosocial stress conditions. As one of the coauthors, Helmut Sies summarized, the situation requires enhanced interdisciplinary investigations among human populations applying advanced methods of molecular, biomedical, and epidemiological research [192]. A crucial future step will be fully elucidating the key role of oxidative stress in the stress-induced cascade of events, which will make preventative and protective measures possible to implement, measures urgently needed as the burden of chronic diseases grows in aging societies.

**Fig. 20:**
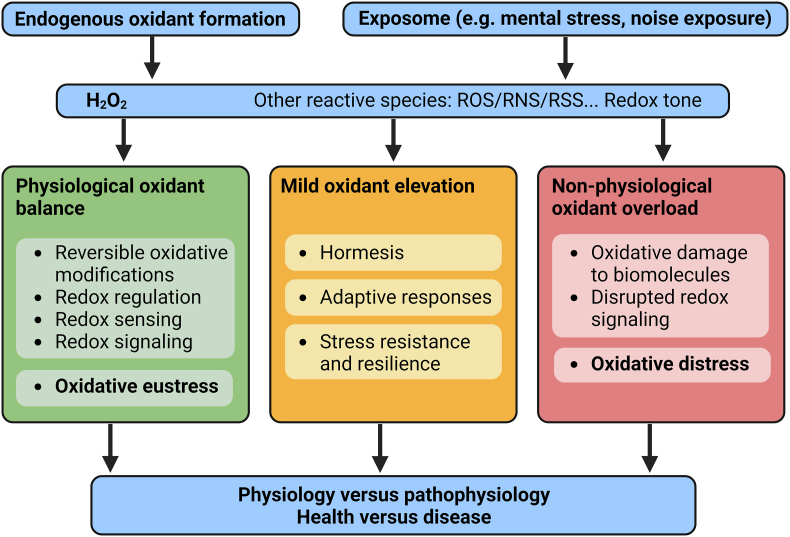
**Concept of oxidative eustress and distress in the context of environmental exposures.** Hydrogen peroxide and other reactive oxygen species (ROS) as well as reactive nitrogen species (RNS) and reactive sulfur species (RSS) are generated by various endogenous sources that are interact with each other, forming cellular signaling networks (see preceding sections). The totality of exposures to various chemical, physical and biologic agents - radiation, psychosocial components, nutrition, exercise, lifestyle and noise exposure - throughout the lifetime, termed exposome, has significant input into oxidant levels. Redox balance is essential for maintenance of homeostasis and for physiological cellular and organismal function. Physiological levels of oxidants support normal cellular processes (oxidative eustress), while excessive oxidant exposure causes damage (oxidative distress). Mild elevation of oxidant levels leads to adaptation to stress and resilience (the concept of hormesis). Redox medicine has the potential to modulate levels of oxidants for therapeutic benefit. It is desirable to control excessive levels of oxidants to prevent toxicity associated with oxidative distress (leading to cell death and tissue degeneration) and maintain proper redox balance. Modified from Ref. [191] with permission.

Taken together, the topic of health effects of noise falls with the concept of the exposome, which encompasses the totality of environmental exposures, a concept introduced by C.P. Wild in 2005 [17]. The main idea of this concept is that detrimental exposures cause biochemical changes and subsequent health or disease outcomes, as discussed above. Evidence supporting the exposome concept was reviewed for CVD [591], cancer [592], metabolic disease [593], pulmonary disease [594] and, in general, chronic non-communicable diseases [595]. Linkage of the exposome concept to redox medicine [596] and tools [597] are there for application to exposome research [190,191,598] (summarized in Fig. 20). In addition, preclinical data point towards a central role of adverse redox signaling for exposure-driven health risks, e.g., as shown for noise [16], air pollution [599], and metal toxicity [600]. This warrants a joint effort of health and redox experts and other disciplines for better understanding of environmental health effects [601,602] and overcoming the analytical challenges associated with exposome research [603].

## 3 Conclusions, redox outlook, and future perspectives

The evidence on health effects of road traffic noise has increased substantially since the evaluation conducted by a WHO appointed expert group in 2018. In contrast, although a few new studies have been published, less research progress has been observed in the railway and aircraft noise fields, and the evidence for these two exposures in relation to all health outcomes are still of very low to moderate quality. The evidence has strengthened for long-term exposure to road traffic noise as a risk factor for IHD, although the excess risk seems lower in recent studies compared to the risk estimate reported in the WHO report. Four outcomes that have recently received increased attention in the noise research field are incident heart failure, stroke, and type 2 diabetes, as well as all-cause mortality. While studies on heart failure, diabetes, and all-cause mortality consistently find associations with road traffic noise, results are less consistent for stroke. Although recent studies found road traffic noise associated with higher CVD mortality, others failed to see an association. Generally, studies assessing road traffic noise across the exposure range using high-quality input data and address-level precision find noise associated with a high risk of cardiometabolic diseases, highlighting the importance of an accurate noise exposure assessment in future studies. Emerging outcomes in a noise context include dementia, cancer, and tinnitus, which deserve more attention in future studies. Interestingly, for dementia, breast cancer, tinnitus, and diabetes, new studies suggest that noise at the least exposed façade, as an indicator for disturbance of sleep, is more strongly associated with these diseases. In contrast, noise at most exposed façade seems to be a more substantial (or similar size) risk factor for CVD.

The believed mechanisms behind the harmful effects of noise on the development of various diseases (e.g., CVD, diabetes, and dementia) include the well-defined “noise reaction model“, with neuronal activation involving the HPA axis and the sympathetic nervous system, followed by a classical stress response via cortisol and catecholamines. Furthermore, noise-induced annoyance (emotional perception), sleep deprivation and/or fragmentation can initiate the stress pathway. Major downstream pathophysiological processes of noise-induced stress are inflammation and oxidative stress induction. The most important sources of ROS (e.g., O_2_^•−^ and H_2_O_2_) formation are the phagocytic NADPH oxidase with a potential contribution of mitochondria and uncoupled NOS enzymes. In contrast, xanthine oxidase involvement has not been observed as part of the noise-induced stress response. Major oxidative damage pathways following noise exposure comprise the uncoupling of NOS enzymes (loss of protective nitric oxide), lipid peroxidation, oxidative DNA damage, and nitration of protein tyrosine residues. Based on preclinical data, noise-induced oxidative damage also represents a potential target of non-pharmacological and pharmacologic interventions (e.g., by antioxidant effects of physical exercise, intermittent fasting, and drug-based activation of antioxidant pathways centered on NRF-2/HO-1 or AMPK). Accordingly, advanced knowledge of adverse redox mechanisms and oxidative damage by noise exposure, e.g., including redox-dependent activation of inflammatory pathways or impairment of circadian rhythms, may allow the successful development of preventive strategies. However, it has to be mentioned that most mechanistic data stem from animal studies, which suffer from major limitations previously reviewed in detail [13]: differences in hearing range between species, difficulties to properly quantify noise annoyance or perception, and generally higher sound pressure levels applied in animal research. These limitations may complicate the comparison of noise effects in humans with those in animals.

More research at the preclinical and clinical level on the health effects of transportation noise and the mechanistic pathways behind them is urgently needed to construct a full picture of the health consequences of this widespread exposure (key points are summarized in Textbox 4). However, we already have extensive evidence showing that road traffic noise is associated with a higher risk of CVD and diabetes. New research has indicated effects on other significant diseases with massive personal and societal costs. Recently, the EU evaluated that approximately 20 % of the population was exposed to transportation noise levels exceeding 55 dB, which is very likely underestimated as the EU mainly estimates noise exposure in larger urban agglomerations. Due to this sizeable number of people exposed to high noise levels, recent calculations have shown that transportation noise contributes considerably to the environmental burden of disease [179]. Importantly, the calculated "burden of disease” will increase substantially if the emerging research on noise and major diseases, such as dementia, breast cancer, and depression, are confirmed in future studies. This stresses the importance of prioritizing actions to better protect the population from high levels of transportation noise via mitigation measures that include lowering speed limits and reducing traffic-flows, noise barriers along major roads, noise-reducing asphalt, low noise-emitting tires, and noise-reducing windows, in addition to enhanced focus on preventing future noise problems in urban planning. These mitigation strategies are especially important to protect the vulnerable groups, e.g. patients with pre-established chronic disease, in light of the data reported by Olbrich et al. indicating a higher aircraft noise-associated risk of recurrent cardiovascular events after acute coronary syndrome [604].

Textbox 4 **Key points of traffic noise health effects and redox-related changes.**

• Evidence of the health effects arising from road traffic noise has grown substantially during the last decade. In contrast, for railway and aircraft noise the evidence is still of very low to moderate quality for all outcomes.
• Several recent studies have investigated the effects of noise on heart failure, stroke, and type 2 diabetes. While studies on heart failure and diabetes find consistent associations with road traffic noise, results are less consistent for stroke.
• Two recent studies on the short-term exposure to aircraft suggested that noise may trigger cardiovascular disease (CVD) hospitalization and death. The study with the highest temporal resolution of the outcome found the strongest association, highlighting the importance of accurate information on the time of day of the CVD event in such studies.
• New outcomes in a noise context include dementia, breast cancer, and tinnitus, as they each have been associated with road traffic and railway noise in at least one high-quality study. These outcomes should be explored in future studies.
• Road traffic noise at the least exposed façade of the house, as an indicator of disturbance of sleep, has been found more strongly associated with diabetes, dementia, breast cancer, and tinnitus. In contrast, noise at the most exposed façade seems to be a stronger (or same size) risk factor for CVD. This suggests that noise may act through different pathways (stress versus sleep) for different diseases.
• Major pathophysiological mechanisms on the pathway between noise and disease are stress hormone and vasoconstrictor signaling, dysregulation of circadian rhythms, inflammation, and oxidative stress.
• Production of reactive oxygen species with subsequent posttranslational oxidative modifications of biomolecules and adverse redox signaling at the systemic level are hallmarks of traffic noise exposure. For examples, this is documented by prevention of adverse noise effects by genetic deletion of phagocytic NADPH oxidase or antioxidant pharmacological interventions.
• In the cardiovascular system and the brain, dysregulation of nitric oxide synthase function and impaired nitric oxide signaling represent key mechanisms of noise-inflicted damage.
• The present work provides a list of redox biomarkers and oxidative stress markers that are reported for noise-exposed animals and humans and provides quality scores for these markers.

Alt-text: Textbox 4

## Financial support

M.T.O. is supported in part by 10.13039/100000002National Institutes of Health K23HL151909 and 10.13039/100000968American Heart Association 23SCISA1143491. P.W., T.M. and T.G. are Principal Investigators and O.H., M.K. and A.D. are (Young) 10.13039/100010447Scientists of the German Center for Cardiovascular Research (DZHK) and were supported by 10.13039/100010447DZHK funding to the partner site Rhine Main (Mainz) and Berlin. R.S. is supported by 10.13039/501100001659Deutsche Forschungsgemeinschaft (DFG; German Research Foundation) [Project number 268555672—SFB 1213, Project B05].

## Declaration of competing interest

The authors declare that they have no known competing financial interests or personal relationships that could have appeared to influence the work reported in this paper.

## Data Availability

Data will be made available on request.

## Footnotes

2 Pink noise (1/f-random noise): swoosh with a decreasing amplitude at higher frequencies. In pink noise, each octave interval (halving or doubling in frequency) carries an equal amount of noise energy. In general, humans will perceive all hearable frequencies with the same loudness. Pink noise sounds like a waterfall and is one of the most commonly observed signals in biological systems.

## References

[1] European Environment Agency (2020).

[2] World Health Organization (2018).

[3] Liu C., Li W., Chen X., Liu M., Zuo L., Chen L., Chen H., Xu W., Hao G. (2023). Dose-response association between transportation noise exposure and type 2 diabetes: a systematic review and meta-analysis of prospective cohort studies. Diabetes Metab Res Rev.

[4] Roswall N., Pyko A., Ogren M., Oudin A., Rosengren A., Lager A., Poulsen A.H., Eriksson C., Segersson D., Rizzuto D., Andersson E.M., Aasvang G.M., Engstrom G., Jorgensen J.T., Selander J., Christensen J.H., Thacher J., Leander K., Overvad K., Eneroth K., Mattisson K., Barregard L., Stockfelt L., Albin M., Ketzel M., Simonsen M.K., Spanne M., Raaschou-Nielsen O., Magnusson P.K.E., Tiittanen P., Molnar P., Ljungman P., Lanki T., Lim Y.H., Andersen Z.J., Pershagen G., Sorensen M. (2021). Long-term exposure to transportation noise and risk of incident stroke: a pooled study of nine scandinavian cohorts. Environ Health Perspect.

[5] Sorensen M., Poulsen A.H., Hvidtfeldt U.A., Munzel T., Thacher J.D., Ketzel M., Brandt J., Christensen J.H., Levin G., Raaschou-Nielsen O. (2021). Transportation noise and risk of stroke: a nationwide prospective cohort study covering Denmark. Int J Epidemiol.

[6] Vienneau D., Saucy A., Schaffer B., Fluckiger B., Tangermann L., Stafoggia M., Wunderli J.M., Roosli M., S.N.C.s. group (2022). Transportation noise exposure and cardiovascular mortality: 15-years of follow-up in a nationwide prospective cohort in Switzerland. Environ Int.

[7] Sorensen M., Raaschou-Nielsen O., Poulsen A.H., Hvidtfeldt U.A., Brandt J., Khan J., Jensen S.S., Munzel T., Thacher J.D. (2023). Long-term exposure to residential transportation noise and mortality: a nationwide cohort study. Environ Pollut.

[8] Thacher J.D., Poulsen A.H., Raaschou-Nielsen O., Hvidtfeldt U.A., Brandt J., Christensen J.H., Khan J., Levin G., Munzel T., Sorensen M. (2022). Exposure to transportation noise and risk for cardiovascular disease in a nationwide cohort study from Denmark. Environ Res.

[9] Andersen Z.J., Jorgensen J.T., Elsborg L., Lophaven S.N., Backalarz C., Laursen J.E., Pedersen T.H., Simonsen M.K., Brauner E.V., Lynge E. (2018). Long-term exposure to road traffic noise and incidence of breast cancer: a cohort study. Breast Cancer Res.

[10] Sorensen M., Poulsen A.H., Kroman N., Hvidtfeldt U.A., Thacher J.D., Roswall N., Brandt J., Frohn L.M., Jensen S.S., Levin G., Raaschou-Nielsen O. (2021). Road and railway noise and risk for breast cancer: a nationwide study covering Denmark. Environ Res.

[11] Cantuaria M.L., Pedersen E.R., Poulsen A.H., Raaschou-Nielsen O., Hvidtfeldt U.A., Levin G., Jensen S.S., Schmidt J.H., Sorensen M. (2023). Transportation noise and risk of tinnitus: a nationwide cohort study from Denmark. Environ Health Perspect.

[12] Sliwinska-Kowalska M., Zaborowski K. (2017). WHO environmental noise guidelines for the European region: a systematic review on environmental noise and permanent hearing loss and tinnitus. Int J Environ Res Public Health.

[13] Munzel T., Sorensen M., Daiber A. (2021). Transportation noise pollution and cardiovascular disease. Nat Rev Cardiol.

[14] Munzel T., Daiber A., Steven S., Tran L.P., Ullmann E., Kossmann S., Schmidt F.P., Oelze M., Xia N., Li H., Pinto A., Wild P., Pies K., Schmidt E.R., Rapp S., Kroller-Schon S. (2017). Effects of noise on vascular function, oxidative stress, and inflammation: mechanistic insight from studies in mice. Eur Heart J.

[15] Kuntic M., Kuntic I., Krishnankutty R., Gericke A., Oelze M., Junglas T., Bayo Jimenez M.T., Stamm P., Nandudu M., Hahad O., Keppeler K., Daub S., Vujacic-Mirski K., Rajlic S., Strohm L., Ubbens H., Tang Q., Jiang S., Ruan Y., Macleod K.G., Steven S., Berkemeier T., Poschl U., Lelieveld J., Kleinert H., von Kriegsheim A., Daiber A., Munzel T. (2023). Co-exposure to urban particulate matter and aircraft noise adversely impacts the cerebro-pulmonary-cardiovascular axis in mice. Redox Biol.

[16] Kroller-Schon S., Daiber A., Steven S., Oelze M., Frenis K., Kalinovic S., Heimann A., Schmidt F.P., Pinto A., Kvandova M., Vujacic-Mirski K., Filippou K., Dudek M., Bosmann M., Klein M., Bopp T., Hahad O., Wild P.S., Frauenknecht K., Methner A., Schmidt E.R., Rapp S., Mollnau H., Munzel T. (2018). Crucial role for Nox2 and sleep deprivation in aircraft noise-induced vascular and cerebral oxidative stress, inflammation, and gene regulation. Eur Heart J.

[17] Wild C.P. (2005). Complementing the genome with an "exposome": the outstanding challenge of environmental exposure measurement in molecular epidemiology. Cancer Epidemiol Biomarkers Prev.

[18] de Bont J., Jaganathan S., Dahlquist M., Persson A., Stafoggia M., Ljungman P. (2022). Ambient air pollution and cardiovascular diseases: an umbrella review of systematic reviews and meta-analyses. J Intern Med.

[19] Blackburn H. (2012). 20th-Century "medical Marco Polos" in the origins of preventive cardiology and cardiovascular disease epidemiology. Am J Cardiol.

[20] Babisch W. (2003). Stress hormones in the research on cardiovascular effects of noise. Noise Health.

[21] Babisch W. (2014). Updated exposure-response relationship between road traffic noise and coronary heart diseases: a meta-analysis. Noise Health.

[22] Munzel T., Schmidt F.P., Steven S., Herzog J., Daiber A., Sorensen M. (2018). Environmental noise and the cardiovascular system. J Am Coll Cardiol.

[23] Babisch W., Pershagen G., Selander J., Houthuijs D., Breugelmans O., Cadum E., Vigna-Taglianti F., Katsouyanni K., Haralabidis A.S., Dimakopoulou K., Sourtzi P., Floud S., Hansell A.L. (2013). Noise annoyance--a modifier of the association between noise level and cardiovascular health?. Sci Total Environ.

[24] Hahad O., Beutel M., Gori T., Schulz A., Blettner M., Pfeiffer N., Rostock T., Lackner K., Sorensen M., Prochaska J.H., Wild P.S., Munzel T. (2018). Annoyance to different noise sources is associated with atrial fibrillation in the Gutenberg Health Study. Int J Cardiol.

[25] Basner M., Witte M., McGuire S. (2019). Aircraft noise effects on sleep—results of a pilot study near Philadelphia international airport. International Journal of Environmental Research and Public Health.

[26] Nassur A.M., Leger D., Lefevre M., Elbaz M., Mietlicki F., Nguyen P., Ribeiro C., Sineau M., Laumon B., Evrard A.S. (2019). Effects of aircraft noise exposure on heart rate during sleep in the population living near airports. Int J Environ Res Public Health.

[27] Van Hee V.C., Adar S.D., Szpiro A.A., Barr R.G., Bluemke D.A., Diez Roux A.V., Gill E.A., Sheppard L., Kaufman J.D. (2009). Exposure to traffic and left ventricular mass and function: the Multi-Ethnic Study of Atherosclerosis. Am J Respir Crit Care Med.

[28] Daiber A., Kroller-Schon S., Oelze M., Hahad O., Li H., Schulz R., Steven S., Munzel T. (2020). Oxidative stress and inflammation contribute to traffic noise-induced vascular and cerebral dysfunction via uncoupling of nitric oxide synthases. Redox Biol.

[29] Daiber A., Kroller-Schon S., Frenis K., Oelze M., Kalinovic S., Vujacic-Mirski K., Kuntic M., Bayo Jimenez M.T., Helmstadter J., Steven S., Korac B., Munzel T. (2019). Environmental noise induces the release of stress hormones and inflammatory signaling molecules leading to oxidative stress and vascular dysfunction-Signatures of the internal exposome. Biofactors.

[30] Schmidt F.P., Basner M., Kroger G., Weck S., Schnorbus B., Muttray A., Sariyar M., Binder H., Gori T., Warnholtz A., Munzel T. (2013). Effect of nighttime aircraft noise exposure on endothelial function and stress hormone release in healthy adults. Eur Heart J.

[31] Wallerath T., Witte K., Schafer S.C., Schwarz P.M., Prellwitz W., Wohlfart P., Kleinert H., Lehr H.A., Lemmer B., Forstermann U. (1999). Down-regulation of the expression of endothelial NO synthase is likely to contribute to glucocorticoid-mediated hypertension. Proc Natl Acad Sci U S A.

[32] Yang S., Zhang L. (2004). Glucocorticoids and vascular reactivity. Curr Vasc Pharmacol.

[33] Kim S., Ohta K., Hamaguchi A., Omura T., Yukimura T., Miura K., Inada Y., Ishimura Y., Chatani F., Iwao H. (1995). Angiotensin II type I receptor antagonist inhibits the gene expression of transforming growth factor-beta 1 and extracellular matrix in cardiac and vascular tissues of hypertensive rats. J Pharmacol Exp Ther.

[34] Kuang S.Q., Geng L., Prakash S.K., Cao J.M., Guo S., Villamizar C., Kwartler C.S., Peters A.M., Brasier A.R., Milewicz D.M. (2013). Aortic remodeling after transverse aortic constriction in mice is attenuated with AT1 receptor blockade. Arterioscler Thromb Vasc Biol.

[35] Nishida M., Okada Y., Akiyoshi K., Eshiro K., Takoaka M., Gariepy C.E., Yanagisawa M., Matsumura Y. (2004). Role of endothelin ETB receptor in the pathogenesis of monocrotaline-induced pulmonary hypertension in rats. Eur J Pharmacol.

[36] Steven S., Oelze M., Brandt M., Ullmann E., Kroller-Schon S., Heeren T., Tran L.P., Daub S., Dib M., Stalleicken D., Wenzel P., Munzel T., Daiber A. (2017). Pentaerythritol tetranitrate in vivo treatment improves oxidative stress and vascular dysfunction by suppression of endothelin-1 signaling in monocrotaline-induced pulmonary hypertension. Oxid Med Cell Longev.

[37] Iwanaga Y., Kihara Y., Hasegawa K., Inagaki K., Yoneda T., Kaburagi S., Araki M., Sasayama S. (1998). Cardiac endothelin-1 plays a critical role in the functional deterioration of left ventricles during the transition from compensatory hypertrophy to congestive heart failure in salt-sensitive hypertensive rats. Circulation.

[38] Booz G.W., Day J.N., Baker K.M. (2002). Interplay between the cardiac renin angiotensin system and JAK-STAT signaling: role in cardiac hypertrophy, ischemia/reperfusion dysfunction, and heart failure. J Mol Cell Cardiol.

[39] Ceriello A. (2006). Oxidative stress and diabetes-associated complications. Endocr Pract.

[40] Yuan T., Yang T., Chen H., Fu D., Hu Y., Wang J., Yuan Q., Yu H., Xu W., Xie X. (2019). New insights into oxidative stress and inflammation during diabetes mellitus-accelerated atherosclerosis. Redox Biol.

[41] Reuter S., Gupta S.C., Chaturvedi M.M., Aggarwal B.B. (2010). Oxidative stress, inflammation, and cancer: how are they linked?. Free Radic Biol Med.

[42] Simpson D.S.A., Oliver P.L. (2020). ROS generation in microglia: understanding oxidative stress and inflammation in neurodegenerative disease. Antioxidants (Basel).

[43] Xia N., Loneliness H. Li (2018). Social isolation, and cardiovascular health. Antioxid Redox Signal.

[44] Campos-Rodriguez R., Godinez-Victoria M., Abarca-Rojano E., Pacheco-Yepez J., Reyna-Garfias H., Barbosa-Cabrera R.E., Drago-Serrano M.E. (2013). Stress modulates intestinal secretory immunoglobulin A. Front Integr Neurosci.

[45] Daiber A., Kroller-Schon S., Oelze M., Hahad O., Li H., Schulz R., Steven S., Munzel T. (2020). Oxidative stress and inflammation contribute to traffic noise-induced vascular and cerebral dysfunction via uncoupling of nitric oxide synthases. Redox Biol.

[46] Osborne M.T., Radfar A., Hassan M.Z.O., Abohashem S., Oberfeld B., Patrich T., Tung B., Wang Y., Ishai A., Scott J.A., Shin L.M., Fayad Z.A., Koenen K.C., Rajagopalan S., Pitman R.K., Tawakol A. (2019). A neurobiological mechanism linking transportation noise to cardiovascular disease in humans. Eur Heart J.

[47] Osborne M.T., Radfar A., Hassan M.Z.O., Abohashem S., Oberfeld B., Patrich T., Tung B., Wang Y., Ishai A., Scott J.A., Shin L.M., Fayad Z.A., Koenen K.C., Rajagopalan S., Pitman R.K., Tawakol A. (2020). A neurobiological mechanism linking transportation noise to cardiovascular disease in humans. Eur Heart J.

[48] Munzel T., Steven S., Hahad O., Daiber A. (2020). The sixth sense is involved in noise-induced stress responses and vascular inflammation: evidence for heightened amygdalar activity in response to transport noise in man. Eur Heart J.

[49] Tawakol A., Ishai A., Takx R.A., Figueroa A.L., Ali A., Kaiser Y., Truong Q.A., Solomon C.J., Calcagno C., Mani V., Tang C.Y., Mulder W.J., Murrough J.W., Hoffmann U., Nahrendorf M., Shin L.M., Fayad Z.A., Pitman R.K. (2017). Relation between resting amygdalar activity and cardiovascular events: a longitudinal and cohort study. Lancet.

[50] Tawakol A., Osborne M.T., Wang Y., Hammed B., Tung B., Patrich T., Oberfeld B., Ishai A., Shin L.M., Nahrendorf M., Warner E.T., Wasfy J., Fayad Z.A., Koenen K., Ridker P.M., Pitman R.K., Armstrong K.A. (2019). Stress-associated neurobiological pathway linking socioeconomic disparities to cardiovascular disease. J Am Coll Cardiol.

[51] Munzel T., Sorensen M., Gori T., Schmidt F.P., Rao X., Brook J., Chen L.C., Brook R.D., Rajagopalan S. (2017). Environmental stressors and cardio-metabolic disease: part I-epidemiologic evidence supporting a role for noise and air pollution and effects of mitigation strategies. Eur Heart J.

[52] Heritier H., Vienneau D., Foraster M., Eze I.C., Schaffner E., Thiesse L., Ruzdik F., Habermacher M., Kopfli M., Pieren R., Schmidt-Trucksass A., Brink M., Cajochen C., Wunderli J.M., Probst-Hensch N., Roosli M., S.N.C.s. group (2018). Diurnal variability of transportation noise exposure and cardiovascular mortality: a nationwide cohort study from Switzerland. Int J Hyg Environ Health.

[53] Khosravipour M., Khanlari P. (2020). The association between road traffic noise and myocardial infarction: a systematic review and meta-analysis. Sci Total Environ.

[54] Dimakopoulou K., Koutentakis K., Papageorgiou I., Kasdagli M.I., Haralabidis A.S., Sourtzi P., Samoli E., Houthuijs D., Swart W., Hansell A.L., Katsouyanni K. (2017). Is aircraft noise exposure associated with cardiovascular disease and hypertension? Results from a cohort study in Athens, Greece. Occup Environ Med.

[55] Bai L., Shin S., Oiamo T.H., Burnett R.T., Weichenthal S., Jerrett M., Kwong J.C., Copes R., Kopp A., Chen H. (2020). Exposure to road traffic noise and incidence of acute myocardial infarction and congestive heart failure: a population-based cohort study in Toronto, Canada. Environ Health Perspect.

[56] Magnoni P., Murtas R., Russo A.G. (2021). Residential exposure to traffic-borne pollution as a risk factor for acute cardiocerebrovascular events: a population-based retrospective cohort study in a highly urbanized area. Int J Epidemiol.

[57] Yankoty L.I., Gamache P., Plante C., Goudreau S., Blais C., Perron S., Fournier M., Ragettli M., Fallah M., Hatzopoulou M., Liu Y., Smargiassi A. (2021). Long─term residential exposure to environmental/transportation noise and the incidence of myocardial infarction. Int J Hyg Environ Health.

[58] Bustaffa E., Curzio O., Donzelli G., Gorini F., Linzalone N., Redini M., Bianchi F., Minichilli F. (2022). Risk associations between vehicular traffic noise exposure and cardiovascular diseases: a residential retrospective cohort study. Int J Environ Res Public Health.

[59] Eminson K., Cai Y.S., Chen Y., Blackmore C., Rodgers G., Jones N., Gulliver J., Fenech B., Hansell A.L. (2023). Does air pollution confound associations between environmental noise and cardiovascular outcomes? - a systematic review. Environ Res.

[60] Hahad O., Rajagopalan S., Lelieveld J., Sorensen M., Frenis K., Daiber A., Basner M., Nieuwenhuijsen M., Brook R.D., Munzel T. (2023). Noise and air pollution as risk factors for hypertension: Part I-epidemiology. Hypertension.

[61] Hahad O., Rajagopalan S., Lelieveld J., Sorensen M., Kuntic M., Daiber A., Basner M., Nieuwenhuijsen M., Brook R.D., Munzel T. (2023). Noise and air pollution as risk factors for hypertension: Part II-pathophysiologic insight. Hypertension.

[62] Harbo Poulsen A., Sorensen M., Hvidtfeldt U.A., Ketzel M., Christensen J.H., Brandt J., Frohn L.M., Massling A., Khan J., Munzel T., Raaschou-Nielsen O. (2023). Concomitant exposure to air pollution, green space and noise, and risk of myocardial infarction. A Cohort study from Denmark. Eur J Prev Cardiol.

[63] Pyko A., Roswall N., Ogren M., Oudin A., Rosengren A., Eriksson C., Segersson D., Rizzuto D., Andersson E.M., Aasvang G.M., Engstrom G., Gudjonsdottir H., Jorgensen J.T., Selander J., Christensen J.H., Brandt J., Leander K., Overvad K., Eneroth K., Mattisson K., Barregard L., Stockfelt L., Albin M., Simonsen M.K., Tiittanen P., Molnar P., Ljungman P., Solvang Jensen S., Gustafsson S., Lanki T., Lim Y.H., Andersen Z.J., Sorensen M., Pershagen G. (2023). Long-term exposure to transportation noise and ischemic heart disease: a pooled analysis of nine scandinavian cohorts. Environ Health Perspect.

[64] Eriksson C., Selander J., Stucki L., Pershagen G. (2021).

[65] Seidler A., Wagner M., Schubert M., Droge P., Pons-Kuhnemann J., Swart E., Zeeb H. (2016). J. Hegewald. Myocardial infarction risk due to aircraft, road, and rail traffic noise. Dtsch Arztebl Int.

[66] Pyko A., Andersson N., Eriksson C., de Faire U., Lind T., Mitkovskaya N., Ogren M., Ostenson C.G., Pedersen N.L., Rizzuto D., Wallas A.K., Pershagen G. (2019). Long-term transportation noise exposure and incidence of ischaemic heart disease and stroke: a cohort study. Occup Environ Med.

[67] Munzel T., Sorensen M., Schmidt F., Schmidt E., Steven S., Kroller-Schon S., Daiber A. (2018). The adverse effects of environmental noise exposure on oxidative stress and cardiovascular risk. Antioxid Redox Signal.

[68] Munzel T., Hahad O., Sorensen M., Lelieveld J., Duerr G.D., Nieuwenhuijsen M., Daiber A. (2022). Environmental risk factors and cardiovascular diseases: a comprehensive expert review. Cardiovasc Res.

[69] van Kempen E., Casas M., Pershagen G., Foraster M. (2018). WHO environmental noise guidelines for the European region: a systematic review on environmental noise and cardiovascular and metabolic effects: a summary. Int J Environ Res Public Health.

[70] Sorensen M., Wendelboe Nielsen O., Sajadieh A., Ketzel M., Tjonneland A., Overvad K., Raaschou-Nielsen O. (2017). Long-term exposure to road traffic noise and nitrogen dioxide and risk of heart failure: a cohort study. Environ. Health Perspect..

[71] Seidler A., Wagner M., Schubert M., Droge P., Romer K., Pons-Kuhnemann J., Swart E., Zeeb H., Aircraft J. Hegewald (2016). Road and railway traffic noise as risk factors for heart failure and hypertensive heart disease-A case-control study based on secondary data. Int J Hyg Environ Health.

[72] Carey I.M., Anderson H.R., Atkinson R.W., Beevers S., Cook D.G., Dajnak D., Gulliver J., Kelly F.J. (2016). Traffic pollution and the incidence of cardiorespiratory outcomes in an adult cohort in London. Occup Environ Med.

[73] Lim Y.H., Jorgensen J.T., So R., Cole-Hunter T., Mehta A.J., Amini H., Brauner E.V., Westendorp R.G.J., Liu S., Mortensen L.H., Hoffmann B., Loft S., Ketzel M., Hertel O., Brandt J., Jensen S.S., Backalarz C., Simonsen M.K., Tasic N., Maric M., Andersen Z.J. (2021). Long-term exposure to air pollution, road traffic noise, and heart failure incidence: the Danish nurse cohort. J Am Heart Assoc.

[74] Collaborators G.B.D.S. (2021). Global, regional, and national burden of stroke and its risk factors, 1990-2019: a systematic analysis for the Global Burden of Disease Study 2019. Lancet Neurol.

[75] Sorensen M., Hvidberg M., Andersen Z.J., Nordsborg R.B., Lillelund K.G., Jakobsen J., Tjonneland A., Overvad K., Raaschou-Nielsen O. (2011). Road traffic noise and stroke: a prospective cohort study. Eur Heart J.

[76] Cai Y., Hodgson S., Blangiardo M., Gulliver J., Morley D., Fecht D., Vienneau D., de Hoogh K., Key T., Hveem K., Elliott P., Hansell A.L. (2018). Road traffic noise, air pollution and incident cardiovascular disease: a joint analysis of the HUNT, EPIC-Oxford and UK Biobank cohorts. Environ Int.

[77] Hao G., Zuo L., Weng X., Fei Q., Zhang Z., Chen L., Wang Z., Jing C. (2022). Associations of road traffic noise with cardiovascular diseases and mortality: longitudinal results from UK Biobank and meta-analysis. Environ Res.

[78] Seidler A.L., Hegewald J., Schubert M., Weihofen V.M., Wagner M., Droge P., Swart E., Zeeb H., Seidler A. (2018). The effect of aircraft, road, and railway traffic noise on stroke - results of a case-control study based on secondary data. Noise Health.

[79] Hoffmann B., Weinmayr G., Hennig F., Fuks K., Moebus S., Weimar C., Dragano N., Hermann D.M., Kalsch H., Mahabadi A.A., Erbel R., Jockel K.H. (2015). Air quality, stroke, and coronary events: results of the heinz nixdorf recall study from the ruhr region. Dtsch Arztebl Int.

[80] Poulsen A.H., Sorensen M., Hvidtfeldt U.A., Christensen J.H., Brandt J., Frohn L.M., Ketzel M., Andersen C., Jensen S.S., Munzel T., Raaschou-Nielsen O. (2023). Concomitant exposure to air pollution, green space, and noise and risk of stroke: a cohort study from Denmark. Lancet Reg Health Eur.

[81] Selander J., Nilsson M.E., Bluhm G., Rosenlund M., Lindqvist M., Nise G., Pershagen G. (2009). Long-term exposure to road traffic noise and myocardial infarction. Epidemiology.

[82] Gan W.Q., Davies H.W., Koehoorn M., Brauer M. (2012). Association of long-term exposure to community noise and traffic-related air pollution with coronary heart disease mortality. Am J Epidemiol.

[83] Sorensen M., Luhdorf P., Ketzel M., Andersen Z.J., Tjonneland A., Overvad K., Raaschou-Nielsen O. (2014). Combined effects of road traffic noise and ambient air pollution in relation to risk for stroke?. Environ Res.

[84] Beelen R., Hoek G., Houthuijs D., van den Brandt P.A., Goldbohm R.A., Fischer P., Schouten L.J., Armstrong B., Brunekreef B. (2009). The joint association of air pollution and noise from road traffic with cardiovascular mortality in a cohort study. Occup Environ Med.

[85] Huss A., Spoerri A., Egger M., Roosli M. (2010). Aircraft noise, air pollution, and mortality from myocardial infarction. Epidemiology.

[86] Hansell A.L., Blangiardo M., Fortunato L., Floud S., de H.K., Fecht D., Ghosh R.E., Laszlo H.E., Pearson C., Beale L., Beevers S., Gulliver J., Best N., Richardson S., Elliott P. (2013). Aircraft noise and cardiovascular disease near Heathrow airport in London: small area study. BMJ.

[87] van Poll R., Ameling C., Breugelmans O., Houthuijs D., van Kempen E., Marra M., Swart W. (2014).

[88] Cole-Hunter T., So R., Amini H., Backalarz C., Brandt J., Brauner E.V., Hertel O., Jensen S.S., Jorgensen J.T., Ketzel M., Laursen J.E., Lim Y.H., Loft S., Mehta A., Mortensen L.H., Simonsen M.K., Sisgaard T., Westendorp R., Andersen Z.J. (2022). Long-term exposure to road traffic noise and all-cause and cause-specific mortality: a Danish Nurse Cohort study. Sci Total Environ.

[89] Heritier H., Vienneau D., Foraster M., Eze I.C., Schaffner E., Thiesse L., Rudzik F., Habermacher M., Kopfli M., Pieren R., Brink M., Cajochen C., Wunderli J.M., Probst-Hensch N., Roosli M., S.N.C.s. group (2017). Transportation noise exposure and cardiovascular mortality: a nationwide cohort study from Switzerland. Eur J Epidemiol.

[90] Thacher J.D., Hvidtfeldt U.A., Poulsen A.H., Raaschou-Nielsen O., Ketzel M., Brandt J., Jensen S.S., Overvad K., Tjonneland A., Munzel T., Sorensen M. (2020). Long-term residential road traffic noise and mortality in a Danish cohort. Environ Res.

[91] Vienneau D., Heritier H., Foraster M., Eze I.C., Schaffner E., Thiesse L., Rudzik F., Habermacher M., Kopfli M., Pieren R., Brink M., Cajochen C., Wunderli J.M., Probst-Hensch N., Roosli M., SNC study group (2019). Facades, Floors and maps - influence of exposure measurement error on the association between transportation noise and myocardial infarction. Environ Int.

[92] Heritier H., Vienneau D., Foraster M., Eze I.C., Schaffner E., de Hoogh K., Thiesse L., Rudzik F., Habermacher M., Kopfli M., Pieren R., Brink M., Cajochen C., Wunderli J.M., Probst-Hensch N., Roosli M. (2019). A systematic analysis of mutual effects of transportation noise and air pollution exposure on myocardial infarction mortality: a nationwide cohort study in Switzerland. Eur Heart J.

[93] Vienneau D., Stafoggia M., Rodopoulou S., Chen J., Atkinson R.W., Bauwelinck M., Klompmaker J.O., Oftedal B., Andersen Z.J., Janssen N.A.H., So R., Lim Y.H., Fluckiger B., Ducret-Stich R., Roosli M., Probst-Hensch N., Kunzli N., Strak M., Samoli E., de Hoogh K., Brunekreef B., Hoek G. (2023). Association between exposure to multiple air pollutants, transportation noise and cause-specific mortality in adults in Switzerland. Environ Health.

[94] Stansfeld S., Clark C., Smuk M., Gallacher J., Babisch W. (2021). Road traffic noise, noise sensitivity, noise annoyance, psychological and physical health and mortality. Environ Health.

[95] Andersson E.M., Ogren M., Molnar P., Segersson D., Rosengren A., Stockfelt L. (2020). Road traffic noise, air pollution and cardiovascular events in a Swedish cohort. Environ Res.

[96] Klompmaker J.O., Hoek G., Bloemsma L.D., Marra M., Wijga A.H., van den Brink C., Brunekreef B., Lebret E., Gehring U., Janssen N.A.H. (2020). Surrounding green, air pollution, traffic noise exposure and non-accidental and cause-specific mortality. Environ Int.

[97] Grady S.T., Hart J.E., Laden F., Roscoe C., Nguyen D.D., Nelson E.J., Bozigar M., VoPham T., Manson J.E., Weuve J., Adar S.D., Forman J.P., Rexrode K., Levy J.I., Peters J.L. (2023). Associations between long-term aircraft noise exposure, cardiovascular disease, and mortality in US cohorts of female nurses. Environ Epidemiol.

[98] Vienneau D., Wicki B., Schaffer B., Wunderli J.M., Roosli M. (2023).

[99] Nawrot T.S., Perez L., Künzli N., Munters E., Nemery B. (2011). Public health importance of triggers of myocardial infarction: a comparative risk assessment. The Lancet.

[100] Bhaskaran K., Gasparrini A., Hajat S., Smeeth L., Armstrong B. (2013). Time series regression studies in environmental epidemiology. International Journal of Epidemiology.

[101] Gasparrini A. (2021). The case time series design. Epidemiology.

[102] Maclure M. (1991). The case-crossover design: a method for studying transient effects on the risk of acute events. Am J Epidemiol.

[103] Carracedo-Martínez E., Taracido M., Tobias A., Saez M., Figueiras A. (2010). Case-crossover analysis of air pollution health effects: a systematic review of methodology and application. Environmental Health Perspectives.

[104] Gasparrini A., Masselot P., Scortichini M., Schneider R., Mistry M.N., Sera F., Macintyre H.L., Phalkey R., Vicedo-Cabrera A.M. (2022). Small-area assessment of temperature-related mortality risks in England and Wales: a case time series analysis. Lancet Planet Health.

[105] Poulsen A.H., Raaschou-Nielsen O., Pena A., Hahmann A.N., Nordsborg R.B., Ketzel M., Brandt J., Sorensen M. (2018). Short-term nighttime wind turbine noise and cardiovascular events: a nationwide case-crossover study from Denmark. Environ Int.

[106] Saucy A., Schaffer B., Tangermann L., Vienneau D., Wunderli J.M., Roosli M. (2021). Does night-time aircraft noise trigger mortality? A case-crossover study on 24 886 cardiovascular deaths. Eur Heart J.

[107] Itzkowitz N., Gong X., Atilola G., Konstantinoudis G., Adams K., Jephcote C., Gulliver J., Hansell A.L., Blangiardo M. (2023). Aircraft noise and cardiovascular morbidity and mortality near Heathrow Airport: a case-crossover study. Environ Int.

[108] World Health Organization (2016).

[109] Clark C., Sbihi H., Tamburic L., Brauer M., Frank L.D., Davies H.W. (2017). Association of long-term exposure to transportation noise and traffic-related air pollution with the incidence of diabetes: a prospective cohort study. Environ Health Perspect.

[110] Eze I.C., Foraster M., Schaffner E., Vienneau D., Heritier H., Rudzik F., Thiesse L., Pieren R., Imboden M., von Eckardstein A., Schindler C., Brink M., Cajochen C., Wunderli J.M., Roosli M., Probst-Hensch N. (2017). Long-term exposure to transportation noise and air pollution in relation to incident diabetes in the SAPALDIA study. Int J Epidemiol.

[111] Roswall N., Raaschou-Nielsen O., Jensen S.S., Tjonneland A., Sorensen M. (2018). Long-term exposure to residential railway and road traffic noise and risk for diabetes in a Danish cohort. Environ Res.

[112] Ohlwein S., Hennig F., Lucht S., Matthiessen C., Pundt N., Moebus S., Jöckel K.H., Hoffmann B. (2019). Indoor and outdoor road traffic noise and incident diabetes mellitus: results from a longitudinal German cohort study. Environ Epidemiol.

[113] Jorgensen J.T., Brauner E.V., Backalarz C., Laursen J.E., Pedersen T.H., Jensen S.S., Ketzel M., Hertel O., Lophaven S.N., Simonsen M.K., Andersen Z.J. (2019). Long-term exposure to road traffic noise and incidence of diabetes in the Danish nurse cohort. Environ Health Perspect.

[114] Shin S., Bai L., Oiamo T.H., Burnett R.T., Weichenthal S., Jerrett M., Kwong J.C., Goldberg M.S., Copes R., Kopp A., Chen H. (2020). Association between road traffic noise and incidence of diabetes mellitus and hypertension in Toronto, Canada: a population-based cohort study. J Am Heart Assoc.

[115] Thacher J.D., Poulsen A.H., Hvidtfeldt U.A., Raaschou-Nielsen O., Brandt J., Geels C., Khan J., Munzel T., Sorensen M. (2021). Long-term exposure to transportation noise and risk for type 2 diabetes in a nationwide cohort study from Denmark. Environ Health Perspect.

[116] Sorensen M., Andersen Z.J., Nordsborg R.B., Becker T., Tjonneland A., Overvad K., Raaschou-Nielsen O. (2013). Long-term exposure to road traffic noise and incident diabetes: a cohort study. Environ Health Perspect.

[117] Sorensen M., Hvidtfeldt U.A., Poulsen A.H., Thygesen L.C., Frohn L.M., Khan J., Raaschou-Nielsen O. (2023). Long-term exposure to transportation noise and risk of type 2 diabetes: a cohort study. Environ Res.

[118] Christensen J.S., Raaschou-Nielsen O., Tjonneland A., Nordsborg R.B., Jensen S.S., Sorensen T.I., Sorensen M. (2015). Long-term exposure to residential traffic noise and changes in body weight and waist circumference: a cohort study. Environ Res.

[119] Pyko A., Eriksson C., Lind T., Mitkovskaya N., Wallas A., Ogren M., Ostenson C.G., Pershagen G. (2017). Long-term exposure to transportation noise in relation to development of obesity-a cohort study. Environ Health Perspect.

[120] Foraster M., Eze I.C., Vienneau D., Schaffner E., Jeong A., Heritier H., Rudzik F., Thiesse L., Pieren R., Brink M., Cajochen C., Wunderli J.M., Roosli M., Probst-Hensch N. (2018). Long-term exposure to transportation noise and its association with adiposity markers and development of obesity. Environ Int.

[121] Sorensen M., Sorensen T.I.A., Ketzel M., Raaschou-Nielsen O. (2020). Exposure to traffic noise and gestational weight gain and postpartum weight retention: a cohort study. Occup Environ Med.

[122] Sorensen M., Poulsen A.H., Hvidtfeldt U.A., Brandt J., Frohn L.M., Ketzel M., Christensen J.H., Im U., Khan J., Munzel T., Raaschou-Nielsen O. (2022). Air pollution, road traffic noise and lack of greenness and risk of type 2 diabetes: a multi-exposure prospective study covering Denmark. Environ Int.

[123] Livingston G., Huntley J., Sommerlad A., Ames D., Ballard C., Banerjee S., Brayne C., Burns A., Cohen-Mansfield J., Cooper C., Costafreda S.G., Dias A., Fox N., Gitlin L.N., Howard R., Kales H.C., Kivimaki M., Larson E.B., Ogunniyi A., Orgeta V., Ritchie K., Rockwood K., Sampson E.L., Samus Q., Schneider L.S., Selbaek G., Teri L., Mukadam N. (2020). Dementia prevention, intervention, and care: 2020 report of the Lancet Commission. Lancet.

[124] Brown R.C., Lockwood A.H., Sonawane B.R. (2005). Neurodegenerative diseases: an overview of environmental risk factors. Environ Health Perspect.

[125] Hahad O., Bayo Jimenez M.T., Kuntic M., Frenis K., Steven S., Daiber A., Munzel T. (2022). Cerebral consequences of environmental noise exposure. Environ Int.

[126] Hahad O., Lelieveld J., Birklein F., Lieb K., Daiber A., Munzel T. (2020). Ambient air pollution increases the risk of cerebrovascular and neuropsychiatric disorders through induction of inflammation and oxidative stress. Int J Mol Sci.

[127] Hegewald J., Schubert M., Freiberg A., Romero Starke K., Augustin F., Riedel-Heller S.G., Zeeb H., Seidler A. (2020). Traffic noise and mental health: a systematic review and meta-analysis. Int J Environ Res Public Health.

[128] Andersson J., Oudin A., Sundstrom A., Forsberg B., Adolfsson R., Nordin M. (2018). Road traffic noise, air pollution, and risk of dementia - results from the Betula project. Environ Res.

[129] Carey I.M., Anderson H.R., Atkinson R.W., Beevers S.D., Cook D.G., Strachan D.P., Dajnak D., Gulliver J., Kelly F.J. (2018). Are noise and air pollution related to the incidence of dementia? A cohort study in London, England. BMJ Open.

[130] Fuks K.B., Wigmann C., Altug H., Schikowski T. (2019). Road traffic noise at the residence, annoyance, and cognitive function in elderly women. Int J Environ Res Public Health.

[131] Tzivian L., Dlugaj M., Winkler A., Weinmayr G., Hennig F., Fuks K.B., Vossoughi M., Schikowski T., Weimar C., Erbel R., Jockel K.H., Moebus S., Hoffmann B., Heinz G. (2016). Nixdorf recall study investigative. Long-term air pollution and traffic noise exposures and mild cognitive impairment in older adults: a cross-sectional analysis of the heinz nixdorf recall study. Environ Health Perspect.

[132] Cantuaria M.L., Waldorff F.B., Wermuth L., Pedersen E.R., Poulsen A.H., Thacher J.D., Raaschou-Nielsen O., Ketzel M., Khan J., Valencia V.H., Schmidt J.H., Sorensen M. (2021). Residential exposure to transportation noise in Denmark and incidence of dementia: national cohort study. BMJ.

[133] Yu Y., Mayeda E.R., Paul K.C., Lee E., Jerrett M., Su J., Wu J., Shih I.F., Haan M., Ritz B. (2020). Traffic-related noise exposure and late-life dementia and cognitive impairment in Mexican-Americans. Epidemiology.

[134] Yuchi W., Sbihi H., Davies H., Tamburic L., Brauer M. (2020). Road proximity, air pollution, noise, green space and neurologic disease incidence: a population-based cohort study. Environ Health.

[135] Roswall N., Christensen J.S., Bidstrup P.E., Raaschou-Nielsen O., Jensen S.S., Tjonneland A., Sorensen M. (2018). Associations between residential traffic noise exposure and smoking habits and alcohol consumption-A population-based study. Environ Pollut.

[136] Eze I.C., Imboden M., Foraster M., Schaffner E., Kumar A., Vienneau D., Heritier H., Rudzik F., Thiesse L., Pieren R., von Eckardstein A., Schindler C., Brink M., Wunderli J.M., Cajochen C., Roosli M., Probst-Hensch N. (2017). Exposure to night-time traffic noise, melatonin-regulating gene variants and change in glycemia in adults. Int J Environ Res Public Health.

[137] Sorensen M., Ketzel M., Overvad K., Tjonneland A., Raaschou-Nielsen O. (2014). Exposure to road traffic and railway noise and postmenopausal breast cancer: a cohort study. Int J Cancer.

[138] Roswall N., Raaschou-Nielsen O., Ketzel M., Overvad K., Halkjaer J., Sorensen M. (2017). Modeled traffic noise at the residence and colorectal cancer incidence: a cohort study. Cancer Causes Control.

[139] Roswall N., Thacher J.D., Ogren M., Pyko A., Akesson A., Oudin A., Tjonneland A., Rosengren A., Poulsen A.H., Eriksson C., Segersson D., Rizzuto D., Helte E., Andersson E.M., Aasvang G.M., Gudjonsdottir H., Khan J., Selander J., Christensen J.H., Brandt J., Leander K., Mattisson K., Eneroth K., Stucki L., Barregard L., Stockfelt L., Albin M., Simonsen M.K., Spanne M., Jousilahti P., Tiittanen P., Molnar P., Ljungman P.L.S., Yli-Tuomi T., Cole-Hunter T., Lanki T., Hvidtfeldt U.A., Lim Y.H., Andersen Z.J., Pershagen G., Sorensen M. (2023). Long-term exposure to traffic noise and risk of incident colon cancer: a pooled study of eleven Nordic cohorts. Environ Res.

[140] Sorensen M., Poulsen A.H., Thacher J., Hvidtfeldt U.A., Ketzel M., Geels C., Jensen S.S., Valencia V.H., Raaschou-Nielsen O. (2021). Transportation noise and risk for colorectal cancer: a nationwide study covering Denmark. Cancer Causes Control.

[141] Hegewald J., Schubert M., Wagner M., Droge P., Prote U., Swart E., Mohler U., Zeeb H., Seidler A. (2017). Breast cancer and exposure to aircraft, road, and railway-noise: a case-control study based on health insurance records. Scand J Work Environ Health.

[142] Hansen J. (2017). Night shift work and risk of breast cancer. Curr Environ Health Rep.

[143] Sorensen M., Harbo Poulsen A., Ketzel M., Oksbjerg Dalton S., Friis S., Raaschou-Nielsen O. (2015). Residential exposure to traffic noise and risk for non-hodgkin lymphoma among adults. Environ Res.

[144] Roswall N., Eriksen K.T., Hjortebjerg D., Jensen S.S., Overvad K., Tjonneland A., Raaschou-Nielsen O., Sorensen M. (2015). Residential exposure to road and railway noise and risk of prostate cancer: a prospective cohort study. PLoS One.

[145] Erdmann F., Raaschou-Nielsen O., Hvidtfeldt U.A., Ketzel M., Brandt J., Khan J., Schuz J., Sorensen M. (2022). Residential road traffic and railway noise and risk of childhood cancer: a nationwide register-based case-control study in Denmark. Environ Res.

[146] Yamane H., Nakai Y., Takayama M., Iguchi H., Nakagawa T., Kojima A. (1995). Appearance of free radicals in the Guinea pig inner ear after noise-induced acoustic trauma. Eur Arch Otorhinolaryngol.

[147] Nelson D.I., Nelson R.Y., Concha-Barrientos M., Fingerhut M. (2005). The global burden of occupational noise-induced hearing loss. Am J Ind Med.

[148] Henderson D., Bielefeld E.C., Harris K.C., Hu B.H. (2006). The role of oxidative stress in noise-induced hearing loss. Ear Hear.

[149] McBride D.I., Williams S. (2001). Audiometric notch as a sign of noise induced hearing loss. Occup Environ Med.

[150] Dobie R.A., Clark W.W. (2014). Exchange rates for intermittent and fluctuating occupational noise: a systematic review of studies of human permanent threshold shift. Ear Hear.

[151] Moore B.C.J. (2020). Diagnosis and quantification of military noise-induced hearing loss. J Acoust Soc Am.

[152] Neitzel R., Seixas N., Goldman B., Daniell W. (2004). Contributions of non-occupational activities to total noise exposure of construction workers. Ann Occup Hyg.

[153] Paik C.B., Pei M., Oghalai J.S. (2022). Review of blast noise and the auditory system. Hear Res.

[154] Le Prell C.G., Dell S., Hensley B., Hall J.W., Campbell K.C., Antonelli P.J., Green G.E., Miller J.M., Guire K. (2012). Digital music exposure reliably induces temporary threshold shift in normal-hearing human subjects. Ear Hear.

[155] Ryan A.F., Kujawa S.G., Hammill T., Le Prell C., Kil J. (2016). Temporary and permanent noise-induced threshold shifts: a review of basic and clinical observations. Otol Neurotol.

[156] Kujawa S.G., Liberman M.C. (2009). Adding insult to injury: cochlear nerve degeneration after "temporary" noise-induced hearing loss. J Neurosci.

[157] Bramhall N., Beach E.F., Epp B., Le Prell C.G., Lopez-Poveda E.A., Plack C.J., Schaette R., Verhulst S., Canlon B. (2019). The search for noise-induced cochlear synaptopathy in humans: mission impossible?. Hear Res.

[158] Schaette R., McAlpine D. (2011). Tinnitus with a normal audiogram: physiological evidence for hidden hearing loss and computational model. J Neurosci.

[159] Baguley D., McFerran D., Hall D. (2013). Tinnitus. Lancet.

[160] Eggermont J.J., Roberts L.E. (2004). The neuroscience of tinnitus. Trends Neurosci.

[161] Lockwood A.H., Salvi R.J., Tinnitus R.F. Burkard (2002). N Engl J Med.

[162] Valderrama J.T., de la Torre A., McAlpine D. (2022). The hunt for hidden hearing loss in humans: from preclinical studies to effective interventions. Front Neurosci.

[163] Elarbed A., Fackrell K., Baguley D.M., Hoare D.J. (2021). Tinnitus and stress in adults: a scoping review. Int J Audiol.

[164] Al-Mana D., Ceranic B., Djahanbakhch O., Luxon L.M. (2008). Hormones and the auditory system: a review of physiology and pathophysiology. Neuroscience.

[165] McKenna L., Handscomb L., Hoare D.J., Hall D.A. (2014). A scientific cognitive-behavioral model of tinnitus: novel conceptualizations of tinnitus distress. Front Neurol.

[166] Bodin T., Bjork J., Ardo J., Albin M. (2015). Annoyance, sleep and concentration problems due to combined traffic noise and the benefit of quiet side. Int J Environ Res Public Health.

[167] Wallhausser-Franke E., Schredl M., Delb W. (2013). Tinnitus and insomnia: is hyperarousal the common denominator?. Sleep Med Rev.

[168] Bhatt J.M., Lin H.W., Bhattacharyya N. (2016). Prevalence, severity, exposures, and treatment patterns of tinnitus in the United States. JAMA Otolaryngol Head Neck Surg.

[169] Klompmaker J.O., Janssen N.A.H., Bloemsma L.D., Marra M., Lebret E., Gehring U., Hoek G. (2021). Effects of exposure to surrounding green, air pollution and traffic noise with non-accidental and cause-specific mortality in the Dutch national cohort. Environ Health.

[170] Thacher J.D., Roswall N., Damm P., Hvidtfeldt U.A., Poulsen A.H., Raaschou-Nielsen O., Ketzel M., Jensen S.S., Frohn L.M., Valencia V.H., Munzel T., Sorensen M. (2021). Transportation noise and gestational diabetes mellitus: a nationwide cohort study from Denmark. Int J Hyg Environ Health.

[171] Collaborators G.B.D.R.F. (2020). Global burden of 87 risk factors in 204 countries and territories, 1990-2019: a systematic analysis for the Global Burden of Disease Study 2019. Lancet.

[172] World Health Organization (2011).

[173] European Environment Agency (2020). EEA Report NO 21/2019.

[174] Eriksson C., Bodin T., Selander J. (2017). Burden of disease from road traffic and railway noise - a quantification of healthy life years lost in Sweden. Scand J Work Environ Health.

[175] Vienneau D., Perez L., Schindler C., Lieb C., Sommer H., Probst-Hensch N., Kunzli N., Roosli M. (2015). Years of life lost and morbidity cases attributable to transportation noise and air pollution: a comparative health risk assessment for Switzerland in 2010. Int J Hyg Environ Health.

[176] Hegewald J., Schubert M., Lochmann M., Seidler A. (2021). The burden of disease due to road traffic noise in hesse, Germany. Int J Environ Res Public Health.

[177] Tainio M. (2015). Burden of disease caused by local transport in Warsaw, Poland. J Transp Health.

[178] Tobollik M., Hintzsche M., Wothge J., Myck T., Plass D. (2019). Burden of disease due to traffic noise in Germany. Int J Environ Res Public Health.

[179] Aasvang G.M., Stockfelt L., Sorensen M., Turunen A.W., Roswall N., Yli-Tuomi T., Ogren M., Lanki T., Selander J., Vincens N., Pyko A., Pershagen G., Sulo G., Bolling A.K. (2023). Burden of disease due to transportation noise in the Nordic countries. Environ Res.

[180] Jephcote C., Clark S.N., Hansell A.L., Jones N., Chen Y., Blackmore C., Eminson K., Evans M., Gong X., Adams K., Rodgers G., Fenech B., Gulliver J. (2023). Spatial assessment of the attributable burden of disease due to transportation noise in England. Environ Int.

[181] Arana M., San Martin R., Salinas J.C. (2014). People exposed to traffic noise in European agglomerations from noise maps. A critical review. Noise Mapping.

[182] Babisch W. (2002). The noise/stress concept, risk assessment and research needs. Noise Health.

[183] Kraus K.S., Canlon B. (2012). Neuronal connectivity and interactions between the auditory and limbic systems. Effects of noise and tinnitus. Hear Res.

[184] Medic G., Wille M., Hemels M.E. (2017). Short- and long-term health consequences of sleep disruption. Nat Sci Sleep.

[185] Ortiz R., Kluwe B., Lazarus S., Teruel M.N., Joseph J.J. (2022). Cortisol and cardiometabolic disease: a target for advancing health equity. Trends Endocrinol Metab.

[186] Kivimaki M., Steptoe A. (2018). Effects of stress on the development and progression of cardiovascular disease. Nat Rev Cardiol.

[187] Frenis K., Kuntic M., Hahad O., Bayo Jimenez M.T., Oelze M., Daub S., Steven S., Munzel T., Daiber A. (2021). Redox switches in noise-induced cardiovascular and neuronal dysregulation. Front Mol Biosci.

[188] Sies H. (1985). Oxidative Stress.

[189] Jones D.P. (2006). Redefining oxidative stress. Antioxid Redox Signal.

[190] Sies H., Berndt C., Jones D.P. (2017). Oxidative stress. Annu Rev Biochem.

[191] Sies H., Jones D.P. (2020). Reactive oxygen species (ROS) as pleiotropic physiological signalling agents. Nat Rev Mol Cell Biol.

[192] Siegrist J., Sies H. (2017). Disturbed redox homeostasis in oxidative distress: a molecular link from chronic psychosocial work stress to coronary heart disease?. Circ Res.

[193] Sies H. (2017). Hydrogen peroxide as a central redox signaling molecule in physiological oxidative stress: oxidative eustress. Redox Biol.

[194] Ouyang J.S., Li Y.P., Li C.Y., Cai C., Chen C.S., Chen S.X., Chen Y.F., Yang L., Xie Y.P. (2012). Mitochondrial ROS-K+ channel signaling pathway regulated secretion of human pulmonary artery endothelial cells. Free Radic Res.

[195] Kahler J., Ewert A., Weckmuller J., Stobbe S., Mittmann C., Koster R., Paul M., Meinertz T., Munzel T. (2001). Oxidative stress increases endothelin-1 synthesis in human coronary artery smooth muscle cells. J Cardiovasc Pharmacol.

[196] Kahler J., Mendel S., Weckmuller J., Orzechowski H.D., Mittmann C., Koster R., Paul M., Meinertz T., Munzel T. (2000). Oxidative stress increases synthesis of big endothelin-1 by activation of the endothelin-1 promoter. J Mol Cell Cardiol.

[197] Daiber A., Di Lisa F., Oelze M., Kroller-Schon S., Steven S., Schulz E., Munzel T. (2017). Crosstalk of mitochondria with NADPH oxidase via reactive oxygen and nitrogen species signalling and its role for vascular function. Br J Pharmacol.

[198] Rajagopalan S., Laursen J.B., Borthayre A., Kurz S., Keiser J., Haleen S., Giaid A., Harrison D.G. (1997). Role for endothelin-1 in angiotensin II-mediated hypertension. Hypertension.

[199] Tran L.T., MacLeod K.M., McNeill J.H. (2009). Endothelin-1 modulates angiotensin II in the development of hypertension in fructose-fed rats. Mol Cell Biochem.

[200] Munzel T., Kroller-Schon S., Oelze M., Gori T., Schmidt F.P., Steven S., Hahad O., Roosli M., Wunderli J.M., Daiber A., Sorensen M. (2020). Adverse cardiovascular effects of traffic noise with a focus on nighttime noise and the new WHO noise guidelines. Annu Rev Public Health.

[201] Singewald N., Kouvelas D., Mostafa A., Sinner C., Philippu A. (2000). Release of glutamate and GABA in the amygdala of conscious rats by acute stress and baroreceptor activation: differences between SHR and WKY rats. Brain Res.

[202] Osborne M.T., Naddaf N., Abohashem S., Radfar A., Ghoneem A., Dar T., Wang Y., Patrich T., Oberfeld B., Tung B., Pitman R.K., Mehta N.N., Shin L.M., Lo J., Rajagopalan S., Koenen K.C., Grinspoon S.K., Fayad Z.A., Tawakol A. (2021). A neurobiological link between transportation noise exposure and metabolic disease in humans. Psychoneuroendocrinology.

[203] Hahad O., Daiber A., Munzel T. (2021). Heightened amygdalar activity mediates the cardiometabolic effects of transportation noise stress. Psychoneuroendocrinology.

[204] Yu J.F., Lee K.C., Hong H.H., Kuo S.B., Wu C.D., Wai Y.Y., Chen Y.F., Peng Y.C. (2015). Human amygdala activation by the sound produced during dental treatment: a fMRI study. Noise Health.

[205] Gannouni N., Mhamdi A., Tebourbi O., El May M., Sakly M., Rhouma K.B. (2013). Qualitative and quantitative assessment of noise at moderate intensities on extra-auditory system in adult rats. Noise Health.

[206] Said M.A., El-Gohary O.A. (2016). Effect of noise stress on cardiovascular system in adult male albino rat: implication of stress hormones, endothelial dysfunction and oxidative stress. Gen Physiol Biophys.

[207] Peterson E.A., Augenstein J.S., Tanis D.C., Augenstein D.G. (1981). Noise raises blood pressure without impairing auditory sensitivity. Science.

[208] Peterson E.A., Augenstein J.S., Hazelton C.L., Hetrick D., Levene R.M., Tanis D.C. (1984). Some cardiovascular effects of noise. J Aud Res.

[209] Herrmann H.J., Rohde H.G., Schulze W., Eichhorn C., Luft F.C. (1994). Effect of noise stress and ethanol intake on hearts of spontaneously hypertensive rats. Basic Res Cardiol.

[210] Lousinha A., Pereira G., Borrecho G., Brito J., Oliveira de Carvalho A., Freitas D., Oliveira P., Mj R.O., Antunes E. (2020). Atrial fibrosis and decreased connexin 43 in rat hearts after exposure to high-intensity infrasound. Exp Mol Pathol.

[211] Dai S., Mo Y., Wang Y., Xiang B., Liao Q., Zhou M., Li X., Li Y., Xiong W., Li G., Guo C., Zeng Z. (2020). Chronic stress promotes cancer development. Front Oncol.

[212] Ishai A., Osborne M.T., Tung B., Wang Y., Hammad B., Patrich T., Oberfeld B., Fayad Z.A., Giles J.T., Lo J., Shin L.M., Grinspoon S.K., Koenen K.C., Pitman R.K., Tawakol A. (2019). Amygdalar metabolic activity independently associates with progression of visceral adiposity. J Clin Endocrinol Metab.

[213] Osborne M.T., Ishai A., Hammad B., Tung B., Wang Y., Baruch A., Fayad Z.A., Giles J.T., Lo J., Shin L.M., Grinspoon S.K., Koenen K.C., Pitman R.K., Tawakol A. (2019). Amygdalar activity predicts future incident diabetes independently of adiposity. Psychoneuroendocrinology.

[214] Yamamura K., Maehara N., Sadamoto T., Harabuchi I. (1982). Effect of intermittent (traffic) noise on man--temporary threshold shift, and change in urinary 17-OHCS and saliva cortisol levels. Eur J Appl Physiol Occup Physiol.

[215] Babisch W., Gallacher J.E., Elwood P.C., Ising H. (1988). Traffic noise and cardiovascular risk. The Caerphilly study, first phase. Outdoor noise levels and risk factors. Arch Environ Health.

[216] Ising H., Ising M. (2002). Chronic cortisol increases in the first half of the night caused by road traffic noise. Noise Health.

[217] Selander J., Bluhm G., Theorell T., Pershagen G., Babisch W., Seiffert I., Houthuijs D., Breugelmans O., Vigna-Taglianti F., Antoniotti M.C., Velonakis E., Davou E., Dudley M.L., Jarup L., Consortium H. (2009). Saliva cortisol and exposure to aircraft noise in six European countries. Environ Health Perspect.

[218] Lefevre M., Carlier M.C., Champelovier P., Lambert J., Laumon B., Evrard A.S. (2017). Effects of aircraft noise exposure on saliva cortisol near airports in France. Occup Environ Med.

[219] Wallas A., Eriksson C., Gruzieva O., Lind T., Pyko A., Sjostrom M., Ogren M., Pershagen G. (2018). Road traffic noise and determinants of saliva cortisol levels among adolescents. Int J Hyg Environ Health.

[220] Thiesse L., Rudzik F., Kraemer J.F., Spiegel K., Leproult R., Wessel N., Pieren R., Heritier H., Eze I.C., Foraster M., Garbazza C., Vienneau D., Brink M., Wunderli J.M., Probst-Hensch N., Roosli M., Cajochen C. (2020). Transportation noise impairs cardiovascular function without altering sleep: the importance of autonomic arousals. Environ Res.

[221] Ising H., Braun C. (2000). Acute and chronic endocrine effects of noise: review of the research conducted at the institute for water, soil and air hygiene. Noise Health.

[222] Babisch W., Fromme H., Beyer A., Ising H. (2001). Increased catecholamine levels in urine in subjects exposed to road traffic noise: the role of stress hormones in noise research. Environ Int.

[223] Munzel T., Knorr M., Schmidt F., von Bardeleben S., Gori T., Schulz E. (2016). Airborne disease: a case of a Takotsubo cardiomyopathie as a consequence of nighttime aircraft noise exposure. Eur Heart J.

[224] Jansen G. (1964). [the effect of noise during physical work]. Int Z Angew Physiol.

[225] Jansen G., Klensch H. (1964). [Alteration of the ballistogram by sound impressions and by music]. Int Z Angew Physiol.

[226] Jansen G. (1968). Effects of noise on health. Ger Med Mon.

[227] Bagheri Hosseinabadi M., Khanjani N., Munzel T., Daiber A., Yaghmorloo M. (2019). Chronic occupational noise exposure: effects on DNA damage, blood pressure, and serum biochemistry. Mutat Res.

[228] Jarup L., Babisch W., Houthuijs D., Pershagen G., Katsouyanni K., Cadum E., Dudley M.L., Savigny P., Seiffert I., Swart W., Breugelmans O., Bluhm G., Selander J., Haralabidis A., Dimakopoulou K., Sourtzi P., Velonakis M., Vigna-Taglianti F., s. team H. (2008). Hypertension and exposure to noise near airports: the HYENA study. Environ Health Perspect.

[229] Dratva J., Phuleria H.C., Foraster M., Gaspoz J.-M., Keidel D., Künzli N., Liu L.J.S., Pons M., Zemp E., Gerbase M.W., Schindler C. (2011). Transportation noise and blood pressure in a population-based sample of adults. Environ Health Perspect.

[230] Haralabidis A.S., Dimakopoulou K., Vigna-Taglianti F., Giampaolo M., Borgini A., Dudley M.L., Pershagen G., Bluhm G., Houthuijs D., Babisch W., Velonakis M., Katsouyanni K., Jarup L., Consortium H. (2008). Acute effects of night-time noise exposure on blood pressure in populations living near airports. Eur Heart J.

[231] Beutel M.E., Junger C., Klein E.M., Wild P., Lackner K., Blettner M., Binder H., Michal M., Wiltink J., Brahler E., Munzel T. (2016). Noise annoyance is associated with depression and anxiety in the general population- the contribution of aircraft noise. PLoS One.

[232] Beutel M.E., Brahler E., Ernst M., Klein E., Reiner I., Wiltink J., Michal M., Wild P.S., Schulz A., Munzel T., Hahad O., Konig J., Lackner K.J., Pfeiffer N., Tibubos A.N. (2020). Noise annoyance predicts symptoms of depression, anxiety and sleep disturbance 5 years later. Findings from the Gutenberg Health Study. Eur J Public Health.

[233] Hahad O., Wild P.S., Prochaska J.H., Schulz A., Lackner K.J., Pfeiffer N., Schmidtmann I., Michal M., Beutel M., Daiber A., Munzel T. (2021). Midregional pro atrial natriuretic peptide: a novel important biomarker for noise annoyance-induced cardiovascular morbidity and mortality?. Clin Res Cardiol.

[234] Herzog J., Schmidt F.P., Hahad O., Mahmoudpour S.H., Mangold A.K., Garcia Andreo P., Prochaska J., Koeck T., Wild P.S., Sorensen M., Daiber A., Munzel T. (2019). Acute exposure to nocturnal train noise induces endothelial dysfunction and pro-thromboinflammatory changes of the plasma proteome in healthy subjects. Basic Res Cardiol.

[235] Heitzer T., Schlinzig T., Krohn K., Meinertz T., Munzel T. (2001). Endothelial dysfunction, oxidative stress, and risk of cardiovascular events in patients with coronary artery disease. Circulation.

[236] Foraster M., Eze I.C., Schaffner E., Vienneau D., Heritier H., Endes S., Rudzik F., Thiesse L., Pieren R., Schindler C., Schmidt-Trucksass A., Brink M., Cajochen C., Marc Wunderli J., Roosli M., Probst-Hensch N. (2017). Exposure to road, railway, and aircraft noise and arterial stiffness in the SAPALDIA study: annual average noise levels and temporal noise characteristics. Environ Health Perspect.

[237] Schmidt F., Kolle K., Kreuder K., Schnorbus B., Wild P., Hechtner M., Binder H., Gori T., Munzel T. (2015). Nighttime aircraft noise impairs endothelial function and increases blood pressure in patients with or at high risk for coronary artery disease. Clin Res Cardiol.

[238] Schmidt F.P., Herzog J. (2020). The impact of aircraft noise on vascular and cardiac function in relation to noise event number – a randomized trial. Cardiovasc Res.

[239] Hahad O., Wild P.S., Prochaska J.H., Schulz A., Hermanns I., Lackner K.J., Pfeiffer N., Schmidtmann I., Beutel M., Gori T., Deanfield J.E., Munzel T. (2019). Endothelial function assessed by digital volume plethysmography predicts the development and progression of type 2 diabetes mellitus. J Am Heart Assoc.

[240] Hahad O., Daiber A., Munzel T. (2021). Heightened amygdalar activity mediates the cardiometabolic effects of transportation noise stress. Psychoneuroendocrinology.

[241] Munzel T. (2008). [Endothelial dysfunction: pathophysiology, diagnosis and prognosis]. Dtsch Med Wochenschr.

[242] Altura B.M., Altura B.T., Gebrewold A., Ising H., Gunther T. (1992). Noise-induced hypertension and magnesium in rats: relationship to microcirculation and calcium. J Appl Physiol.

[243] Wu C.C., Chen S.J., Yen M.H. (1992). Effects of noise on blood pressure and vascular reactivities. Clin Exp Pharmacol Physiol.

[244] Wu C.C., Chen S.J., Yen M.H. (1994). Attenuation of endothelium-dependent relaxation in mesenteric artery during noise-induced hypertension. J Biomed Sci.

[245] Frenis K., Kalinovic S., Ernst B.P., Kvandova M., Al Zuabi A., Kuntic M., Oelze M., Stamm P., Bayo Jimenez M.T., Kij A., Keppeler K., Klein V., Strohm L., Ubbens H., Daub S., Hahad O., Kroller-Schon S., Schmeisser M.J., Chlopicki S., Eckrich J., Strieth S., Daiber A., Steven S., Munzel T. (2021). Long-term effects of aircraft noise exposure on vascular oxidative stress, endothelial function and blood pressure: No evidence for adaptation or tolerance development. Front Mol Biosci.

[246] Bayo Jimenez M.T., Gericke A., Frenis K., Rajlic S., Kvandova M., Kroller-Schon S., Oelze M., Kuntic M., Kuntic I., Mihalikova D., Tang Q., Jiang S., Ruan Y., Duerr G.D., Steven S., Schmeisser M.J., Hahad O., Li H., Daiber A., Munzel T. (2023). Effects of aircraft noise cessation on blood pressure, cardio- and cerebrovascular endothelial function, oxidative stress, and inflammation in an experimental animal model. Sci Total Environ.

[247] Steven S., Frenis K., Kalinovic S., Kvandova M., Oelze M., Helmstadter J., Hahad O., Filippou K., Kus K., Trevisan C., Schluter K.D., Boengler K., Chlopicki S., Frauenknecht K., Schulz R., Sorensen M., Daiber A., Kroller-Schon S., Munzel T. (2020). Exacerbation of adverse cardiovascular effects of aircraft noise in an animal model of arterial hypertension. Redox Biol.

[248] Molitor M., Bayo-Jimenez M.T., Hahad O., Witzler C., Finger S., Garlapati V.S., Rajlic S., Knopp T., Bieler T.K., Aluia M., Wild J., Lagrange J., Blessing R., Rapp S., Schulz A., Kleinert H., Karbach S., Steven S., Ruf W., Wild P., Daiber A., Munzel T., Wenzel P. (2023). Aircraft noise exposure induces pro-inflammatory vascular conditioning and amplifies vascular dysfunction and impairment of cardiac function after myocardial infarction. Cardiovasc Res.

[249] Arnsten A.F., Goldman-Rakic P.S. (1998). Noise stress impairs prefrontal cortical cognitive function in monkeys: evidence for a hyperdopaminergic mechanism. Arch Gen Psychiatry.

[250] McEwen B.S., Weiss J.M., Schwartz L.S. (1968). Selective retention of corticosterone by limbic structures in rat brain. Nature.

[251] Saljo A., Bao F., Shi J., Hamberger A., Hansson H.A., Haglid K.G. (2002). Expression of c-Fos and c-Myc and deposition of beta-APP in neurons in the adult rat brain as a result of exposure to short-lasting impulse noise. J Neurotrauma.

[252] Cheng L., Wang S.H., Chen Q.C., Liao X.M. (2011). Moderate noise induced cognition impairment of mice and its underlying mechanisms. Physiol Behav.

[253] Zhang Y., Zhu M., Sun Y., Tang B., Zhang G., An P., Cheng Y., Shan Y., Merzenich M.M., Zhou X. (2021). Environmental noise degrades hippocampus-related learning and memory. Proc Natl Acad Sci U S A.

[254] Jafari Z., Kolb B.E., Mohajerani M.H. (2018). Chronic traffic noise stress accelerates brain impairment and cognitive decline in mice. Exp Neurol.

[255] Epel E., Lapidus R., McEwen B., Brownell K. (2001). Stress may add bite to appetite in women: a laboratory study of stress-induced cortisol and eating behavior. Psychoneuroendocrinology.

[256] Oliver G., Wardle J., Gibson E.L. (2000). Stress and food choice: a laboratory study. Psychosom Med.

[257] Zellner D.A., Loaiza S., Gonzalez Z., Pita J., Morales J., Pecora D., Wolf A. (2006). Food selection changes under stress. Physiol Behav.

[258] Dallman M.F., Pecoraro N., Akana S.F., La Fleur S.E., Gomez F., Houshyar H., Bell M.E., Bhatnagar S., Laugero K.D., Manalo S. (2003). Chronic stress and obesity: a new view of "comfort food". Proc Natl Acad Sci U S A.

[259] la Fleur S.E., Houshyar H., Roy M., Dallman M.F. (2005). Choice of lard, but not total lard calories, damps adrenocorticotropin responses to restraint. Endocrinology.

[260] Pecoraro N., Reyes F., Gomez F., Bhargava A., Dallman M.F. (2004). Chronic stress promotes palatable feeding, which reduces signs of stress: feedforward and feedback effects of chronic stress. Endocrinology.

[261] Jafari Z., Faraji J., Mirza Agha B., Metz G.A.S., Kolb B.E., Mohajerani M.H. (2017). The adverse effects of auditory stress on mouse uterus receptivity and behaviour. Sci Rep.

[262] Hao G., Zuo L., Xiong P., Chen L., Liang X., Jing C. (2022). Associations of PM2.5 and road traffic noise with mental health: evidence from UK Biobank. Environ Res.

[263] Eze I.C., Foraster M., Schaffner E., Vienneau D., Pieren R., Imboden M., Wunderli J.M., Cajochen C., Brink M., Röösli M., Probst-Hensch N. (2020). Incidence of depression in relation to transportation noise exposure and noise annoyance in the SAPALDIA study. Environ Int.

[264] Roswall N., Ammitzboll G., Christensen J.S., Raaschou-Nielsen O., Jensen S.S., Tjonneland A., Sorensen M. (2017). Residential exposure to traffic noise and leisure-time sports - a population-based study. Int J Hyg Environ Health.

[265] Paul K.C., Haan M., Mayeda E.R., Ritz B.R. (2019). Ambient air pollution, noise, and late-life cognitive decline and dementia risk. Annu Rev Public Health.

[266] Alonso A.D., Cohen L.S., Corbo C., Morozova V., ElIdrissi A., Phillips G., Kleiman F.E. (2018). Hyperphosphorylation of tau associates with changes in its function beyond microtubule stability. Front Cell Neurosci.

[267] Kao Y.C., Ho P.C., Tu Y.K., Jou I.M., Tsai K.J. (2020). Lipids and Alzheimer's disease. Int J Mol Sci.

[268] Hicks D.A., Nalivaeva N.N., Turner A.J. (2012). Lipid rafts and Alzheimer's disease: protein-lipid interactions and perturbation of signaling. Front Physiol.

[269] Dugger B.N., Dickson D.W. (2017). Pathology of neurodegenerative diseases. Cold Spring Harb Perspect Biol.

[270] Cui B., Li K., Gai Z., She X., Zhang N., Xu C., Chen X., An G., Ma Q., Wang R. (2015). Chronic noise exposure acts cumulatively to exacerbate Alzheimer's disease-like amyloid-beta pathology and neuroinflammation in the rat Hippocampus. Sci Rep.

[271] Gai Z., Su D., Wang Y., Li W., Cui B., Li K., She X., Wang R. (2017). Effects of chronic noise on the corticotropin-releasing factor system in the rat hippocampus: relevance to Alzheimer's disease-like tau hyperphosphorylation. Environ Health Prev Med.

[272] Rodrigue K.M., Kennedy K.M., Park D.C. (2009). Beta-amyloid deposition and the aging brain. Neuropsychol Rev.

[273] Jafari Z., Kolb B.E., Mohajerani M.H. (2020). Noise exposure accelerates the risk of cognitive impairment and Alzheimer's disease: adulthood, gestational, and prenatal mechanistic evidence from animal studies. Neurosci Biobehav Rev.

[274] Akyazi I., Eraslan E. (2014). Transmission of stress between cagemates: a study in rats. Physiol Behav.

[275] Manikandan S., Padma M.K., Srikumar R., Jeya Parthasarathy N., Muthuvel A., Sheela Devi R. (2006). Effects of chronic noise stress on spatial memory of rats in relation to neuronal dendritic alteration and free radical-imbalance in hippocampus and medial prefrontal cortex. Neurosci Lett.

[276] Cui B., Wu M., She X. (2009). Effects of chronic noise exposure on spatial learning and memory of rats in relation to neurotransmitters and NMDAR2B alteration in the hippocampus. J Occup Health.

[277] Jafari Z., Okuma M., Karem H., Mehla J., Kolb B.E., Mohajerani M.H. (2019). Prenatal noise stress aggravates cognitive decline and the onset and progression of beta amyloid pathology in a mouse model of Alzheimer's disease. Neurobiol Aging.

[278] Samson J., Sheela Devi R., Ravindran R., Senthilvelan M. (2005). Effect of noise stress on free radical scavenging enzymes in brain. Environ Toxicol Pharmacol.

[279] Sundareswaran L., Srinivasan S., Wankhar W., Sheeladevi R. (2017). Effect of Scoparia dulcis on noise stress induced adaptive immunity and cytokine response in immunized Wistar rats. J Ayurveda Integr Med.

[280] Wankhar W., Srinivasan S., Sundareswaran L., Wankhar D., Rajan R., Sheeladevi R. (2017). Role of Scoparia dulcis linn on noise-induced nitric oxide synthase (NOS) expression and neurotransmitter assessment on motor function in Wistar albino rats. Biomed Pharmacother.

[281] Frenis K., Helmstadter J., Ruan Y., Schramm E., Kalinovic S., Kroller-Schon S., Bayo Jimenez M.T., Hahad O., Oelze M., Jiang S., Wenzel P., Sommer C.J., Frauenknecht K.B.M., Waisman A., Gericke A., Daiber A., Munzel T., Steven S. (2021). Ablation of lysozyme M-positive cells prevents aircraft noise-induced vascular damage without improving cerebral side effects. Basic Res Cardiol.

[282] Koutsaliaris I.K., Moschonas I.C., Pechlivani L.M., Tsouka A.N., Tselepis A.D. (2022). Inflammation, oxidative stress, vascular aging and atherosclerotic ischemic stroke. Curr Med Chem.

[283] Yang Q., Huang Q., Hu Z., Tang X. (2019). Potential neuroprotective treatment of stroke: targeting excitotoxicity, oxidative stress, and inflammation. Front Neurosci.

[284] Wang X., Lai Y., Zhang X., Zhao J. (2018). Effect of low-frequency but high-intensity noise exposure on swine brain blood barrier permeability and its mechanism of injury. Neurosci Lett.

[285] Hahad O., Prochaska J.H., Daiber A., Muenzel T. (2019). Environmental noise-induced effects on stress hormones, oxidative stress, and vascular dysfunction: key factors in the relationship between cerebrocardiovascular and psychological disorders. Oxid Med Cell Longev.

[286] Stansfeld S.A., Berglund B., Clark C., Lopez-Barrio I., Fischer P., Ohrstrom E., Haines M.M., Head J., Hygge S., van Kamp I., Berry B.F., team R.s. (2005). Aircraft and road traffic noise and children's cognition and health: a cross-national study. Lancet.

[287] Clark C., Paunovic K. (2018). WHO environmental noise guidelines for the European region: a systematic review on environmental noise and cognition. Int J Environ Res Public Health.

[288] Seidler A., Hegewald J., Seidler A.L., Schubert M., Wagner M., Droge P., Haufe E., Schmitt J., Swart E., Zeeb H. (2017). Association between aircraft, road and railway traffic noise and depression in a large case-control study based on secondary data. Environ Res.

[289] Dzhambov A.M., Lercher P. (2019). Road traffic noise exposure and depression/anxiety: an updated systematic review and meta-analysis. Int J Environ Res Public Health.

[290] Sorensen M., Poulsen A.H., Hvidtfeldt U.A., Munzel T., Thacher J.D., Ketzel M., Brandt J., Christensen J.H., Levin G., Raaschou-Nielsen O. (2021). Transportation noise and risk of stroke: a nationwide prospective cohort study covering Denmark. Int J Epidemiol.

[291] Cai Y., Zijlema W.L., Sorgjerd E.P., Doiron D., de Hoogh K., Hodgson S., Wolffenbuttel B., Gulliver J., Hansell A.L., Nieuwenhuijsen M., Rahimi K., Kvaloy K. (2020). Impact of road traffic noise on obesity measures: observational study of three European cohorts. Environ Res.

[292] Muzet A. (2007). Environmental noise, sleep and health. Sleep Med Rev.

[293] Freeman D., Sheaves B., Waite F., Harvey A.G., Harrison P.J. (2020). Sleep disturbance and psychiatric disorders. Lancet Psychiatry.

[294] Anderson K.N., Bradley A.J. (2013). Sleep disturbance in mental health problems and neurodegenerative disease. Nat Sci Sleep.

[295] Lechat B., Scott H., Decup F., Hansen K.L., Micic G., Dunbar C., Liebich T., Catcheside P., Zajamsek B. (2021). Environmental noise-induced cardiovascular responses during sleep. Sleep.

[296] Coborn J.E., Lessie R.E., Sinton C.M., Rance N.E., Perez-Leighton C.E., Teske J.A. (2019). Noise-induced sleep disruption increases weight gain and decreases energy metabolism in female rats. Int J Obes (Lond).

[297] Kim C.S., Grady S.T., Hart J.E., Laden F., VoPham T., Nguyen D.D., Manson J.E., James P., Forman J.P., Rexrode K.M., Levy J.I., Peters J.L. (2022). Long-term aircraft noise exposure and risk of hypertension in the Nurses' Health Studies. Environ Res.

[298] Kourieh A., Giorgis-Allemand L., Bouaoun L., Lefevre M., Champelovier P., Lambert J., Laumon B., Evrard A.S. (2022). Incident hypertension in relation to aircraft noise exposure: results of the DEBATS longitudinal study in France. Occup Environ Med.

[299] Nguyen D.D., Whitsel E.A., Wellenius G.A., Levy J.I., Leibler J.H., Grady S.T., Stewart J.D., Fox M.P., Collins J.M., Eliot M.N., Malwitz A., Manson J.E., Peters J.L. (2023). Long-term aircraft noise exposure and risk of hypertension in postmenopausal women. Environ Res.

[300] Spiegel K., Tasali E., Leproult R., Van Cauter E. (2009). Effects of poor and short sleep on glucose metabolism and obesity risk. Nat Rev Endocrinol.

[301] Miller M.A., Cappuccio F.P. (2007). Inflammation, sleep, obesity and cardiovascular disease. Curr Vasc Pharmacol.

[302] Calvin A.D., Covassin N., Kremers W.K., Adachi T., Macedo P., Albuquerque F.N., Bukartyk J., Davison D.E., Levine J.A., Singh P., Wang S., Somers V.K. (2014). Experimental sleep restriction causes endothelial dysfunction in healthy humans. J Am Heart Assoc.

[303] Carreras A., Zhang S.X., Peris E., Qiao Z., Gileles-Hillel A., Li R.C., Wang Y., Gozal D. (2014). Chronic sleep fragmentation induces endothelial dysfunction and structural vascular changes in mice. Sleep.

[304] Cappuccio F.P., Cooper D., D'Elia L., Strazzullo P., Miller M.A. (2011). Sleep duration predicts cardiovascular outcomes: a systematic review and meta-analysis of prospective studies. Eur Heart J.

[305] Kan H., Hu W., Wang Y., Wu W., Yin Y., Liang Y., Wang C., Huang D., Li W. (2015). NADPH oxidase-derived production of reactive oxygen species is involved in learning and memory impairments in 16-month-old female rats. Mol Med Rep.

[306] Nair D., Zhang S.X., Ramesh V., Hakim F., Kaushal N., Wang Y., Gozal D. (2011). Sleep fragmentation induces cognitive deficits via nicotinamide adenine dinucleotide phosphate oxidase-dependent pathways in mouse. Am J Respir Crit Care Med.

[307] Zhang S.X., Khalyfa A., Wang Y., Carreras A., Hakim F., Neel B.A., Brady M.J., Qiao Z., Hirotsu C., Gozal D. (2014). Sleep fragmentation promotes NADPH oxidase 2-mediated adipose tissue inflammation leading to insulin resistance in mice. Int J Obes (Lond).

[308] Kanazawa L.K., Vecchia D.D., Wendler E.M., Hocayen P.A., Dos Reis Livero F.A., Stipp M.C., Barcaro I.M., Acco A., Andreatini R. (2016). Quercetin reduces manic-like behavior and brain oxidative stress induced by paradoxical sleep deprivation in mice. Free Radic Biol Med.

[309] Alzoubi K.H., Khabour O.F., Albawaana A.S., Alhashimi F.H., Athamneh R.Y. (2016). Tempol prevents chronic sleep-deprivation induced memory impairment. Brain Res Bull.

[310] Schiavone S., Jaquet V., Trabace L., Krause K.H. (2013). Severe life stress and oxidative stress in the brain: from animal models to human pathology. Antioxid Redox Signal.

[311] McAlpine C.S., Kiss M.G., Rattik S., He S., Vassalli A., Valet C., Anzai A., Chan C.T., Mindur J.E., Kahles F., Poller W.C., Frodermann V., Fenn A.M., Gregory A.F., Halle L., Iwamoto Y., Hoyer F.F., Binder C.J., Libby P., Tafti M., Scammell T.E., Nahrendorf M., Swirski F.K. (2019). Sleep modulates haematopoiesis and protects against atherosclerosis. Nature.

[312] Moller H., Pedersen C.S. (2011). Low-frequency noise from large wind turbines. J Acoust Soc Am.

[313] Jakobsen J. (2012). Danish regulation of low frequency noise from wind turbines. Journal of low frequency noise, vibration and active control.

[314] Michaud D.S., Keith S.E., Feder K., Voicescu S.A., Marro L., Than J., Guay M., Bower T., Denning A., Lavigne E., Whelan C., Janssen S.A., Leroux T., van den Berg F. (2016). Personal and situational variables associated with wind turbine noise annoyance. J Acoust Soc Am.

[315] van Kamp I., van den Berg F. (2021). Health effects related to wind turbine sound: an update. Int J Environ Res Public Health.

[316] Freiberg A., Schefter C., Girbig M., Murta V.C., Seidler A. (2019). Health effects of wind turbines on humans in residential settings: results of a scoping review. Environ Res.

[317] Janssen S.A., Vos H., Eisses A.R., Pedersen E. (2011). A comparison between exposure-response relationships for wind turbine annoyance and annoyance due to other noise sources. J Acoust Soc Am.

[318] Michaud D.S., Marro L., McNamee J. (2018). Derivation and application of a composite annoyance reaction construct based on multiple wind turbine features. Can J Public Health.

[319] Ascone L., Kling C., Wieczorek J., Koch C., Kuhn S. (2021). A longitudinal, randomized experimental pilot study to investigate the effects of airborne infrasound on human mental health, cognition, and brain structure. Sci Rep.

[320] Jones R.M., Neish A.S. (2017). Redox signaling mediated by the gut microbiota. Free Radic Biol Med.

[321] Campbell E.L., Colgan S.P. (2019). Control and dysregulation of redox signalling in the gastrointestinal tract. Nat Rev Gastroenterol Hepatol.

[322] Cryan J.F., Dinan T.G. (2012). Mind-altering microorganisms: the impact of the gut microbiota on brain and behaviour. Nat Rev Neurosci.

[323] Collins S.M., Surette M., Bercik P. (2012). The interplay between the intestinal microbiota and the brain. Nat Rev Microbiol.

[324] Cui B., Su D., Li W., She X., Zhang M., Wang R., Zhai Q. (2018). Effects of chronic noise exposure on the microbiome-gut-brain axis in senescence-accelerated prone mice: implications for Alzheimer's disease. J Neuroinflammation.

[325] Chi H., Cao W., Zhang M., Su D., Yang H., Li Z., Li C., She X., Wang K., Gao X., Ma K., Zheng P., Li X., Cui B. (2021). Environmental noise stress disturbs commensal microbiota homeostasis and induces oxi-inflammmation and AD-like neuropathology through epithelial barrier disruption in the EOAD mouse model. J Neuroinflammation.

[326] Cui B., Gai Z., She X., Wang R., Xi Z. (2016). Effects of chronic noise on glucose metabolism and gut microbiota-host inflammatory homeostasis in rats. Sci Rep.

[327] Zymantiene J., Zelvyte R., Pampariene I., Aniuliene A., Juodziukyniene N., Kantautaite J., Oberauskas V. (2017). Effects of long-term construction noise on health of adult female Wistar rats. Pol J Vet Sci.

[328] Hadizadeh M., Hamidi G.A., Salami M. (2019). Probiotic supplementation improves the cognitive function and the anxiety-like behaviors in the stressed rats. Iran J Basic Med Sci.

[329] Li X., Zheng P., Cao W., Cao Y., She X., Yang H., Ma K., Wu F., Gao X., Fu Y., Yin J., Wei F., Jiang S., Cui B. (2023). Lactobacillus rhamnosus GG ameliorates noise-induced cognitive deficits and systemic inflammation in rats by modulating the gut-brain axis. Front Cell Infect Microbiol.

[330] Berlow M., Wada H., Derryberry E.P. (2022). Experimental exposure to noise alters gut microbiota in a captive songbird. Microb Ecol.

[331] Karl J.P., Hatch A.M., Arcidiacono S.M., Pearce S.C., Pantoja-Feliciano I.G., Doherty L.A., Soares J.W. (2018). Effects of psychological, environmental and physical stressors on the gut microbiota. Front Microbiol.

[332] Zhang A., Zou T., Guo D., Wang Q., Shen Y., Hu H., Ye B., Xiang M. (2020). The immune system can hear noise. Front Immunol.

[333] Hemmingsen J.G., Moller P., Jantzen K., Jonsson B.A., Albin M., Wierzbicka A., Gudmundsson A., Loft S., Rissler J. (2015). Controlled exposure to diesel exhaust and traffic noise--Effects on oxidative stress and activation in mononuclear blood cells. Mutat Res.

[334] Ersoy A., Koc E.R., Sahin S., Duzgun U., Acar B., Ilhan A. (2014). Possible effects of rosuvastatin on noise-induced oxidative stress in rat brain. Noise Health.

[335] Hahad O., Frenis K., Kuntic M., Daiber A., Munzel T. (2021). Accelerated aging and age-related diseases (CVD and neurological) due to air pollution and traffic noise exposure. Int J Mol Sci.

[336] Wieczerzak K.B., Patel S.V., MacNeil H., Scott K.E., Schormans A.L., Hayes S.H., Herrmann B., Allman B.L. (2021). Differential plasticity in auditory and prefrontal cortices, and cognitive-behavioral deficits following noise-induced hearing loss. Neuroscience.

[337] Patel S.V., DeCarlo C.M., Book S.A., Schormans A.L., Whitehead S.N., Allman B.L., Hayes S.H. (2022). Noise exposure in early adulthood causes age-dependent and brain region-specific impairments in cognitive function. Front Neurosci.

[338] Su D., Li W., She X., Chen X., Zhai Q., Cui B., Wang R. (2018). Chronic noise exposure exacerbates AD-like neuropathology in SAMP8 mice in relation to Wnt signaling in the PFC and hippocampus. Sci Rep.

[339] Fernandez K.A., Jeffers P.W., Lall K., Liberman M.C., Kujawa S.G. (2015). Aging after noise exposure: acceleration of cochlear synaptopathy in "recovered" ears. J Neurosci.

[340] Keithley E.M. (2020). Pathology and mechanisms of cochlear aging. J Neurosci Res.

[341] Sergeyenko Y., Lall K., Liberman M.C., Kujawa S.G. (2013). Age-related cochlear synaptopathy: an early-onset contributor to auditory functional decline. J Neurosci.

[342] Fetoni A.R., Pisani A., Rolesi R., Paciello F., Viziano A., Moleti A., Sisto R., Troiani D., Paludetti G., Grassi C. (2022). Early noise-induced hearing loss accelerates presbycusis altering aging processes in the cochlea. Front Aging Neurosci.

[343] Griendling K.K., FitzGerald G.A. (2003). Oxidative stress and cardiovascular injury: Part I: basic mechanisms and in vivo monitoring of ROS. Circulation.

[344] Griendling K.K., FitzGerald G.A. (2003). Oxidative stress and cardiovascular injury: Part II: animal and human studies. Circulation.

[345] Daiber A., Hahad O., Andreadou I., Steven S., Daub S., Munzel T. (2021). Redox-related biomarkers in human cardiovascular disease - classical footprints and beyond. Redox Biol.

[346] Ischiropoulos H., Beckman J.S. (2003). Oxidative stress and nitration in neurodegeneration: cause, effect, or association?. J Clin Invest.

[347] Gupta S.C., Hevia D., Patchva S., Park B., Koh W., Aggarwal B.B. (2012). Upsides and downsides of reactive oxygen species for cancer: the roles of reactive oxygen species in tumorigenesis, prevention, and therapy. Antioxid Redox Signal.

[348] Heusch G., Andreadou I., Bell R., Bertero E., Botker H.E., Davidson S.M., Downey J., Eaton P., Ferdinandy P., Gersh B.J., Giacca M., Hausenloy D.J., Ibanez B., Krieg T., Maack C., Schulz R., Sellke F., Shah A.M., Thiele H., Yellon D.M., Di Lisa F. (2023). Health position paper and redox perspectives on reactive oxygen species as signals and targets of cardioprotection. Redox Biol.

[349] Lin M.I., Fulton D., Babbitt R., Fleming I., Busse R., Pritchard K.A., Sessa W.C. (2003). Phosphorylation of threonine 497 in endothelial nitric-oxide synthase coordinates the coupling of L-arginine metabolism to efficient nitric oxide production. J Biol Chem.

[350] Fisslthaler B., Loot A.E., Mohamed A., Busse R., Fleming I. (2008). Inhibition of endothelial nitric oxide synthase activity by proline-rich tyrosine kinase 2 in response to fluid shear stress and insulin. Circ Res.

[351] Loot A.E., Schreiber J.G., Fisslthaler B., Fleming I. (2009). Angiotensin II impairs endothelial function via tyrosine phosphorylation of the endothelial nitric oxide synthase. J Exp Med.

[352] Schulz E., Wenzel P., Munzel T., Daiber A. (2014). Mitochondrial redox signaling: interaction of mitochondrial reactive oxygen species with other sources of oxidative stress. Antioxid Redox Signal.

[353] Nguyen T.T., Stevens M.V., Kohr M., Steenbergen C., Sack M.N., Murphy E. (2011). Cysteine 203 of cyclophilin D is critical for cyclophilin D activation of the mitochondrial permeability transition pore. J Biol Chem.

[354] Chan S.L., Baumbach G.L. (2013). Nox2 deficiency prevents hypertension-induced vascular dysfunction and hypertrophy in cerebral arterioles. Int J Hypertens.

[355] Landmesser U., Dikalov S., Price S.R., McCann L., Fukai T., Holland S.M., Mitch W.E., Harrison D.G. (2003). Oxidation of tetrahydrobiopterin leads to uncoupling of endothelial cell nitric oxide synthase in hypertension. J Clin Invest.

[356] Murdoch C.E., Alom-Ruiz S.P., Wang M., Zhang M., Walker S., Yu B., Brewer A., Shah A.M. (2011). Role of endothelial Nox2 NADPH oxidase in angiotensin II-induced hypertension and vasomotor dysfunction. Basic Res Cardiol.

[357] Landmesser U., Cai H., Dikalov S., McCann L., Hwang J., Jo H., Holland S.M., Harrison D.G. (2002). Role of p47(phox) in vascular oxidative stress and hypertension caused by angiotensin II. Hypertension.

[358] Guzik T.J., Sadowski J., Guzik B., Jopek A., Kapelak B., Przybylowski P., Wierzbicki K., Korbut R., Harrison D.G., Channon K.M. (2006). Coronary artery superoxide production and nox isoform expression in human coronary artery disease. Arterioscler Thromb Vasc Biol.

[359] Sorescu D., Weiss D., Lassegue B., Clempus R.E., Szocs K., Sorescu G.P., Valppu L., Quinn M.T., Lambeth J.D., Vega J.D., Taylor W.R., Griendling K.K. (2002). Superoxide production and expression of nox family proteins in human atherosclerosis. Circulation.

[360] Vlajkovic S.M., Lin S.C., Wong A.C., Wackrow B., Thorne P.R. (2013). Noise-induced changes in expression levels of NADPH oxidases in the cochlea. Hear Res.

[361] Bielefeld E.C. (2013). Reduction in impulse noise-induced permanent threshold shift with intracochlear application of an NADPH oxidase inhibitor. J Am Acad Audiol.

[362] Shih C.P., Kuo C.Y., Lin Y.Y., Lin Y.C., Chen H.K., Wang H., Chen H.C., Wang C.H. (2021). Inhibition of cochlear HMGB1 expression attenuates oxidative stress and inflammation in an experimental murine model of noise-induced hearing loss. Cells.

[363] Eckrich J., Frenis K., Rodriguez-Blanco G., Ruan Y., Jiang S., Bayo Jimenez M.T., Kuntic M., Oelze M., Hahad O., Li H., Gericke A., Steven S., Strieth S., von Kriegsheim A., Munzel T., Ernst B.P., Daiber A. (2021). Aircraft noise exposure drives the activation of white blood cells and induces microvascular dysfunction in mice. Redox Biol.

[364] Jimenez M.T.B., Gericke A., Frenis K., Rajlic S., Kvandova M., Kroller-Schon S., Oelze M., Kuntic M., Kuntic I., Mihalikova D., Tang Q., Jiang S., Ruan Y., Duerr G.D., Steven S., Schmeisser M.J., Hahad O., Li H., Daiber A., Munzel T. (2023). Effects of aircraft noise cessation on blood pressure, cardio- and cerebrovascular endothelial function, oxidative stress, and inflammation in an experimental animal model. Sci Total Environ.

[365] Chen D.D., Dong Y.G., Yuan H., Chen A.F. (2012). Endothelin 1 activation of endothelin A receptor/NADPH oxidase pathway and diminished antioxidants critically contribute to endothelial progenitor cell reduction and dysfunction in salt-sensitive hypertension. Hypertension.

[366] Duerrschmidt N., Wippich N., Goettsch W., Broemme H.J., Morawietz H. (2000). Endothelin-1 induces NAD(P)H oxidase in human endothelial cells. Biochem Biophys Res Commun.

[367] Cerrato R., Cunnington C., Crabtree M.J., Antoniades C., Pernow J., Channon K.M., Bohm F. (2012). Endothelin-1 increases superoxide production in human coronary artery bypass grafts. Life Sci.

[368] Steven S., Oelze M., Hausding M., Roohani S., Kashani F., Kroller-Schon S., Helmstadter J., Jansen T., Baum C., Iglarz M., Schulz E., Munzel T., Daiber A. (2018). The endothelin receptor antagonist macitentan improves isosorbide-5-mononitrate (ISMN) and isosorbide dinitrate (ISDN) induced endothelial dysfunction, oxidative stress, and vascular inflammation. Oxid Med Cell Longev.

[369] Li L., Watts S.W., Banes A.K., Galligan J.J., Fink G.D., Chen A.F. (2003). NADPH oxidase-derived superoxide augments endothelin-1-induced venoconstriction in mineralocorticoid hypertension. Hypertension.

[370] Li L., Chu Y., Fink G.D., Engelhardt J.F., Heistad D.D., Chen A.F. (2003). Endothelin-1 stimulates arterial VCAM-1 expression via NADPH oxidase-derived superoxide in mineralocorticoid hypertension. Hypertension.

[371] Li L., Fink G.D., Watts S.W., Northcott C.A., Galligan J.J., Pagano P.J., Chen A.F. (2003). Endothelin-1 increases vascular superoxide via endothelin(A)-NADPH oxidase pathway in low-renin hypertension. Circulation.

[372] Lee H.Y., Lee J.S., Kim H.G., Kim W.Y., Lee S.B., Choi Y.H., Son C.G. (2017). The ethanol extract of Aquilariae Lignum ameliorates hippocampal oxidative stress in a repeated restraint stress mouse model. BMC Complement Altern Med.

[373] Boengler K., Lochnit G., Schulz R. (2018). Mitochondria "THE" target of myocardial conditioning. Am J Physiol Heart Circ Physiol.

[374] Davidson S.M., Ferdinandy P., Andreadou I., Botker H.E., Heusch G., Ibanez B., Ovize M., Schulz R., Yellon D.M., Hausenloy D.J., Garcia-Dorado D., Action C.C. (2019). Multitarget strategies to reduce myocardial ischemia/reperfusion injury: JACC review topic of the week. J Am Coll Cardiol.

[375] Dikalova A.E., Bikineyeva A.T., Budzyn K., Nazarewicz R.R., McCann L., Lewis W., Harrison D.G., Dikalov S.I. (2010). Therapeutic targeting of mitochondrial superoxide in hypertension. Circ Res.

[376] Dikalov S.I., Nazarewicz R.R., Bikineyeva A., Hilenski L., Lassegue B., Griendling K.K., Harrison D.G., Dikalova A.E. (2014). Nox2-Induced production of mitochondrial superoxide in angiotensin II-mediated endothelial oxidative stress and hypertension. Antioxid Redox Signal.

[377] Antunes E., Borrecho G., Oliveira P., Alves de Matos A.P., Brito J., Aguas A., Martins dos Santos J. (2013). Effects of low-frequency noise on cardiac collagen and cardiomyocyte ultrastructure: an immunohistochemical and electron microscopy study. Int J Clin Exp Pathol.

[378] Boengler K., Stahlhofen S., van de Sand A., Gres P., Ruiz-Meana M., Garcia-Dorado D., Heusch G., Schulz R. (2009). Presence of connexin 43 in subsarcolemmal, but not in interfibrillar cardiomyocyte mitochondria. Basic Res Cardiol.

[379] Heinzel F.R., Luo Y., Li X., Boengler K., Buechert A., Garcia-Dorado D., Di Lisa F., Schulz R., Heusch G. (2005). Impairment of diazoxide-induced formation of reactive oxygen species and loss of cardioprotection in connexin 43 deficient mice. Circ Res.

[380] Lenzi P., Frenzilli G., Gesi M., Ferrucci M., Lazzeri G., Fornai F., Nigro M. (2003). DNA damage associated with ultrastructural alterations in rat myocardium after loud noise exposure. Environ Health Perspect.

[381] Li H., Kilgallen A.B., Munzel T., Wolf E., Lecour S., Schulz R., Daiber A., Van Laake L.W. (2020). Influence of mental stress and environmental toxins on circadian clocks: implications for redox regulation of the heart and cardioprotection. Br J Pharmacol.

[382] Salvetti F., Chelli B., Gesi M., Pellegrini A., Giannaccini G., Lucacchini A., Martini C. (2000). Effect of noise exposure on rat cardiac peripheral benzodiazepine receptors. Life Sci.

[383] Schulz R., Schluter K.D. (2023). Importance of mitochondria in cardiac pathologies: focus on uncoupling proteins and monoamine oxidases. Int J Mol Sci.

[384] Knittel J., Itani N., Schreckenberg R., Heger J., Rohrbach S., Schulz R., Schluter K.D. (2023). Monoamine oxidase A contributes to serotonin-but not norepinephrine-dependent damage of rat ventricular myocytes. Biomolecules.

[385] Ullrich O., Grune T., Henke W., Esterbauer H., Siems W.G. (1994). Identification of metabolic pathways of the lipid peroxidation product 4-hydroxynonenal by mitochondria isolated from rat kidney cortex. FEBS Lett.

[386] Munzel T., Daiber A. (2018). The potential of aldehyde dehydrogenase 2 as a therapeutic target in cardiovascular disease. Expert Opin Ther Targets.

[387] Rieder M., Gauchel N., Bode C., Duerschmied D. (2021). Serotonin: a platelet hormone modulating cardiovascular disease. J Thromb Thrombolysis.

[388] Nigmatullina R.R., Kirillova V.V., Jourjikiya R.K., Mukhamedyarov M.A., Kudrin V.S., Klodt P.M., Palotas A. (2009). Disrupted serotonergic and sympathoadrenal systems in patients with chronic heart failure may serve as new therapeutic targets and novel biomarkers to assess severity, progression and response to treatment. Cardiology.

[389] He G., Hu J., Li T., Ma X., Meng J., Jia M., Lu J., Ohtsu H., Chen Z., Luo X. (2012). Arrhythmogenic effect of sympathetic histamine in mouse hearts subjected to acute ischemia. Mol Med.

[390] Genovese A., Spadaro G. (1997). Highlights in cardiovascular effects of histamine and H1-receptor antagonists. Allergy.

[391] He Z., Ma C., Yu T., Song J., Leng J., Gu X., Li J. (2019). Activation mechanisms and multifaceted effects of mast cells in ischemia reperfusion injury. Exp Cell Res.

[392] Sturza A., Leisegang M.S., Babelova A., Schroder K., Benkhoff S., Loot A.E., Fleming I., Schulz R., Muntean D.M., Brandes R.P. (2013). Monoamine oxidases are mediators of endothelial dysfunction in the mouse aorta. Hypertension.

[393] Heger J., Hirschhauser C., Bornbaum J., Sydykov A., Dempfle A., Schneider A., Braun T., Schluter K.D., Schulz R. (2021). Cardiomyocytes-specific deletion of monoamine oxidase B reduces irreversible myocardial ischemia/reperfusion injury. Free Radic Biol Med.

[394] Heger J., Szabados T., Brosinsky P., Bencsik P., Ferdinandy P., Schulz R. (2023). Sex difference in cardioprotection against acute myocardial infarction in MAO-B knockout mice in vivo. Int J Mol Sci.

[395] Fetoni A.R., Eramo S.L., Paciello F., Rolesi R., Samengo D., Paludetti G., Troiani D., Pani G. (2016). The redox protein p66(shc) mediates cochlear vascular dysfunction and transient noise-induced hearing loss. Sci Rep.

[396] Gierhardt M., Pak O., Sydykov A., Kraut S., Schaffer J., Garcia C., Veith C., Zeidan E.M., Brosien M., Quanz K., Esfandiary A., Saraji A., Hadzic S., Kojonazarov B., Wilhelm J., Ghofrani H.A., Schermuly R.T., Seeger W., Grimminger F., Herden C., Schulz R., Weissmann N., Heger J., Sommer N. (2022). Genetic deletion of p66shc and/or cyclophilin D results in decreased pulmonary vascular tone. Cardiovasc Res.

[397] Garcia Castro C.F., Nardiello C., Hadzic S., Kojonazarov B., Kraut S., Gierhardt M., Schaffer J., Bednorz M., Quanz K., Heger J., Korfei M., Wilhelm J., Hecker M., Bartkuhn M., Arnhold S., Guenther A., Seeger W., Schulz R., Weissmann N., Sommer N., Pak O. (2023). The role of the redox enzyme p66Shc in biological aging of the lung. Aging Dis.

[398] Boengler K., Bencsik P., Paloczi J., Kiss K., Pipicz M., Pipis J., Ferdinandy P., Schluter K.D., Schulz R. (2017). Lack of contribution of p66shc and its mitochondrial translocation to ischemia-reperfusion injury and cardioprotection by ischemic preconditioning. Front Physiol.

[399] Di Lisa F., Giorgio M., Ferdinandy P., Schulz R. (2017). New aspects of p66Shc in ischaemia reperfusion injury and other cardiovascular diseases. Br J Pharmacol.

[400] Boengler K., Bornbaum J., Schluter K.D., Schulz R. (2019). P66shc and its role in ischemic cardiovascular diseases. Basic Res Cardiol.

[401] Neri M., Cerretani D., Fiaschi A.I., Laghi P.F., Lazzerini P.E., Maffione A.B., Micheli L., Bruni G., Nencini C., Giorgi G., D'Errico S., Fiore C., Pomara C., Riezzo I., Turillazzi E., Fineschi V. (2007). Correlation between cardiac oxidative stress and myocardial pathology due to acute and chronic norepinephrine administration in rats. J Cell Mol Med.

[402] Raval A.P., Dave K.R., DeFazio R.A., Perez-Pinzon M.A. (2007). epsilonPKC phosphorylates the mitochondrial K(+) (ATP) channel during induction of ischemic preconditioning in the rat hippocampus. Brain Res.

[403] Waza A.A., Andrabi K., Hussain M.U. (2014). Protein kinase C (PKC) mediated interaction between conexin43 (Cx43) and K(+)(ATP) channel subunit (Kir6.1) in cardiomyocyte mitochondria: implications in cytoprotection against hypoxia induced cell apoptosis. Cell Signal.

[404] Daiber A. (2010). Redox signaling (cross-talk) from and to mitochondria involves mitochondrial pores and reactive oxygen species. Biochim Biophys Acta.

[405] Munzel T., Daiber A. (2023). Vascular redox signaling, endothelial nitric oxide synthase uncoupling, and endothelial dysfunction in the setting of transportation noise exposure or chronic treatment with organic nitrates. Antioxid Redox Signal.

[406] Heinrich U.R., Schmidtmann I., Meuser R., Ernst B.P., Wunsch D., Siemer S., Gribko A., Stauber R.H., Strieth S. (2019). Early alterations of endothelial nitric oxide synthase expression patterns in the Guinea pig cochlea after noise exposure. J Histochem Cytochem.

[407] Komeima K., Hayashi Y., Naito Y., Watanabe Y. (2000). Inhibition of neuronal nitric-oxide synthase by calcium/calmodulin-dependent protein kinase IIalpha through Ser847 phosphorylation in NG108-15 neuronal cells. J Biol Chem.

[408] Kasamatsu S., Watanabe Y., Sawa T., Akaike T., Ihara H. (2014). Redox signal regulation via nNOS phosphorylation at Ser847 in PC12 cells and rat cerebellar granule neurons. Biochem J.

[409] Zielonka J., Kalyanaraman B. (2010). Hydroethidine- and MitoSOX-derived red fluorescence is not a reliable indicator of intracellular superoxide formation: another inconvenient truth. Free Radic Biol Med.

[410] Michalski R., Thiebaut D., Michałowski B., Ayhan M.M., Hardy M., Ouari O., Rostkowski M., Smulik-Izydorczyk R., Artelska A., Marcinek A., Zielonka J., Kalyanaraman B., Sikora A. (2020). Oxidation of ethidium-based probes by biological radicals: mechanism, kinetics and implications for the detection of superoxide. Sci Rep.

[411] Zielonka J., Srinivasan S., Hardy M., Ouari O., Lopez M., Vasquez-Vivar J., Avadhani N.G., Kalyanaraman B. (2008). Cytochrome c-mediated oxidation of hydroethidine and mito-hydroethidine in mitochondria: identification of homo- and heterodimers. Free Radic Biol Med.

[412] Zielonka J., Vasquez-Vivar J., Kalyanaraman B. (2008). Detection of 2-hydroxyethidium in cellular systems: a unique marker product of superoxide and hydroethidine. Nat Protoc.

[413] Zielonka J., Zielonka M., Kalyanaraman B. (2019). HPLC-based monitoring of oxidation of hydroethidine for the detection of NADPH oxidase-derived superoxide radical anion. Methods Mol Biol.

[414] Robinson K.M., Janes M.S., Pehar M., Monette J.S., Ross M.F., Hagen T.M., Murphy M.P., Beckman J.S. (2006). Selective fluorescent imaging of superoxide in vivo using ethidium-based probes. Proc Natl Acad Sci U S A.

[415] Michalski R., Zielonka J., Hardy M., Joseph J., Hydropropidine B. Kalyanaraman (2013). A novel, cell-impermeant fluorogenic probe for detecting extracellular superoxide. Free Radic Biol Med.

[416] Shchepinova M.M., Cairns A.G., Prime T.A., Logan A., James A.M., Hall A.R., Vidoni S., Arndt S., Caldwell S.T., Prag H.A., Pell V.R., Krieg T., Mulvey J.F., Yadav P., Cobley J.N., Bright T.P., Senn H.M., Anderson R.F., Murphy M.P., MitoNeoD R.C. Hartley (2017). A mitochondria-targeted superoxide probe. Cell Chem Biol.

[417] Cheng G., Zielonka M., Dranka B., Kumar S.N., Myers C.R., Bennett B., Garces A.M., Dias Duarte Machado L.G., Thiebaut D., Ouari O., Hardy M., Zielonka J., Kalyanaraman B. (2018). Detection of mitochondria-generated reactive oxygen species in cells using multiple probes and methods: potentials, pitfalls, and the future. J Biol Chem.

[418] Hardy M., Zielonka J., Karoui H., Sikora A., Michalski R., Podsiadły R., Lopez M., Vasquez-Vivar J., Kalyanaraman B., Ouari O. (2018). Detection and characterization of reactive oxygen and nitrogen species in biological systems by monitoring species-specific products. Antioxid Redox Signal.

[419] Münzel T., Afanas'ev I.B., Kleschyov A.L., Harrison D.G. (2002). Detection of superoxide in vascular tissue. Arterioscler Thromb Vasc Biol.

[420] Kalinovic S., Stamm P., Oelze M., Steven S., Kröller-Schön S., Kvandova M., Zielonka J., Münzel T., Daiber A. (2021). Detection of extracellular superoxide in isolated human immune cells and in an animal model of arterial hypertension using hydropropidine probe and HPLC analysis. Free Radic Biol Med.

[421] Wardman P., Burkitt M.J., Patel K.B., Lawrence A., Jones C.M., Everett S.A., Vojnovic B. (2002). Pitfalls in the use of common luminescent probes for oxidative and nitrosative stress. Journal of Fluorescence.

[422] Thayer W.S. (1990). Superoxide-dependent and superoxide-independent pathways for reduction of nitroblue tetrazolium in isolated rat cardiac myocytes. Arch Biochem Biophys.

[423] Tan A.S., Berridge M.V. (2000). Superoxide produced by activated neutrophils efficiently reduces the tetrazolium salt, WST-1 to produce a soluble formazan: a simple colorimetric assay for measuring respiratory burst activation and for screening anti-inflammatory agents. J Immunol Methods.

[424] Stockert J.C., Horobin R.W., Colombo L.L., Blázquez-Castro A. (2018). Tetrazolium salts and formazan products in Cell Biology: viability assessment, fluorescence imaging, and labeling perspectives. Acta Histochem.

[425] Auclair C., Voisin E., Greenwald R.A. (1985). CRC Handbook of Methods for Oxygen Radical Research.

[426] Zielonka J. (2023). M. Juric Methods to Measure Reactive Oxygen Species Production by NADPH Oxidases.

[427] Zielonka J., Kalyanaraman B. (2018). Small-molecule luminescent probes for the detection of cellular oxidizing and nitrating species. Free Radic Biol Med.

[428] Jiang X., Li M., Wang Y., Wang C., Wang Y., Shen T., Shen L., Liu X., Wang Y., Li X. (2023). 1,2,4,5-Tetrazine-tethered probes for fluorogenically imaging superoxide in live cells with ultrahigh specificity. Nat Commun.

[429] Dikalov S., Griendling K.K., Harrison D.G. (2007). Measurement of reactive oxygen species in cardiovascular studies. Hypertension.

[430] Wardman P. (2007). Fluorescent and luminescent probes for measurement of oxidative and nitrosative species in cells and tissues: progress, pitfalls, and prospects. Free Radical Biology and Medicine.

[431] Mohanty J.G., Jaffe J.S., Schulman E.S., Raible D.G. (1997). A highly sensitive fluorescent micro-assay of H2O2 release from activated human leukocytes using a dihydroxyphenoxazine derivative. Journal of Immunological Methods.

[432] Lawrence A., Jones C.M., Wardman P., Burkitt M.J. (2003). Evidence for the role of a peroxidase compound I-type intermediate in the oxidation of glutathione, NADH, ascorbate, and dichlorofluorescin by cytochrome c/H2O2. Implications for oxidative stress during apoptosis. J Biol Chem.

[433] Folkes L.K., Patel K.B., Wardman P., Wrona M. (2009). Kinetics of reaction of nitrogen dioxide with dihydrorhodamine and the reaction of the dihydrorhodamine radical with oxygen: implications for quantifying peroxynitrite formation in cells. Arch Biochem Biophys.

[434] Brewer T.F., Garcia F.J., Onak C.S., Carroll K.S., Chang C.J. (2015). Chemical approaches to discovery and study of sources and targets of hydrogen peroxide redox signaling through NADPH oxidase proteins. Annu Rev Biochem.

[435] Sikora A., Zielonka J., Dębowska K., Michalski R., Smulik-Izydorczyk R., Pięta J., Podsiadły R., Artelska A., Pierzchała K., Kalyanaraman B. (2020). Boronate-based probes for biological oxidants: a novel class of molecular tools for redox biology. Frontiers in Chemistry.

[436] Zielonka J., Sikora A., Hardy M., Joseph J., Dranka B.P., Kalyanaraman B. (2012). Boronate probes as diagnostic tools for real time monitoring of peroxynitrite and hydroperoxides. Chem Res. Toxicol.

[437] Zielonka J., Sikora A., Podsiadly R., Hardy M., Kalyanaraman B. (2021). Identification of peroxynitrite by profiling oxidation and nitration products from mitochondria-targeted arylboronic acid. Methods Mol Biol.

[438] Zielonka J., Zielonka M., VerPlank L., Cheng G., Hardy M., Ouari O., Ayhan M.M., Podsiadły R., Sikora A., Lambeth J.D., Kalyanaraman B. (2016). Mitigation of NADPH oxidase 2 activity as a strategy to inhibit peroxynitrite formation. J Biol Chem.

[439] Van de Bittner G.C., Dubikovskaya E.A., Bertozzi C.R., Chang C.J. (2010). In vivo imaging of hydrogen peroxide production in a murine tumor model with a chemoselective bioluminescent reporter. Proc Natl Acad Sci U S A.

[440] Szala M., Grzelakowska A., Modrzejewska J., Siarkiewicz P., Słowiński D., Świerczyńska M., Zielonka J., Podsiadły R. (2020). Characterization of the reactivity of luciferin boronate - a probe for inflammatory oxidants with improved stability. Dyes and Pigments.

[441] Daiber A., Oelze M., August M., Wendt M., Sydow K., Wieboldt H., Kleschyov A.L., Munzel T. (2004). Detection of superoxide and peroxynitrite in model systems and mitochondria by the luminol analogue L-012. Free Radic Res.

[442] Zielonka J., Lambeth J.D., Kalyanaraman B. (2013). On the use of L-012, a luminol-based chemiluminescent probe, for detecting superoxide and identifying inhibitors of NADPH oxidase: a reevaluation. Free Radic Biol Med.

[443] Dikalov S.I., Harrison D.G. (2014). Methods for detection of mitochondrial and cellular reactive oxygen species. Antioxid Redox Signal.

[444] Griendling K.K., Touyz R.M., Zweier J.L., Dikalov S., Chilian W., Chen Y.R., Harrison D.G., A. Bhatnagar S. (2016). American heart association council on basic cardiovascular. Measurement of reactive oxygen species, reactive nitrogen species, and redox-dependent signaling in the cardiovascular system: a scientific statement from the American heart association. Circ Res.

[445] Campeau S., Nyhuis T.J., Sasse S.K., Kryskow E.M., Herlihy L., Masini C.V., Babb J.A., Greenwood B.N., Fleshner M., Day H.E. (2010). Hypothalamic pituitary adrenal axis responses to low-intensity stressors are reduced after voluntary wheel running in rats. J Neuroendocrinol.

[446] Burow A., Day H.E., Campeau S. (2005). A detailed characterization of loud noise stress: intensity analysis of hypothalamo-pituitary-adrenocortical axis and brain activation. Brain Res.

[447] Eraslan E., Akyazi I., Erg L.E.E., Matur E. (2015). Noise stress changes mRNA expressions of corticotropin-releasing hormone, its receptors in amygdala, and anxiety-related behaviors. Noise Health.

[448] Smith M.G., Cordoza M., Basner M. (2022). Environmental noise and effects on sleep: an update to the WHO systematic review and meta-analysis. Environ Health Perspect.

[449] Wright J.W., Dengerink H.A., Miller J.M., Goodwin P.C. (1985). Potential role of angiotensin II in noise-induced increases in inner ear blood flow. Hear Res.

[450] Ye S., Zhong H., Yanamadala S., Campese V.M. (2006). Oxidative stress mediates the stimulation of sympathetic nerve activity in the phenol renal injury model of hypertension. Hypertension.

[451] Lob H.E., Marvar P.J., Guzik T.J., Sharma S., McCann L.A., Weyand C., Gordon F.J., Harrison D.G. (2010). Induction of hypertension and peripheral inflammation by reduction of extracellular superoxide dismutase in the central nervous system. Hypertension.

[452] Grande M.T., Pascual G., Riolobos A.S., Clemente-Lorenzo M., Bardaji B., Barreiro L., Tornavaca O., Meseguer A., Lopez-Novoa J.M. (2011). Increased oxidative stress, the renin-angiotensin system, and sympathetic overactivation induce hypertension in kidney androgen-regulated protein transgenic mice. Free Radic Biol Med.

[453] Rajagopalan S., Kurz S., Munzel T., Tarpey M., Freeman B.A., Griendling K.K., Harrison D.G. (1996). Angiotensin II-mediated hypertension in the rat increases vascular superoxide production via membrane NADH/NADPH oxidase activation. Contribution to alterations of vasomotor tone. J Clin Invest.

[454] Mollnau H., Oelze M., August M., Wendt M., Daiber A., Schulz E., Baldus S., Kleschyov A.L., Materne A., Wenzel P., Hink U., Nickenig G., Fleming I., Munzel T. (2005). Mechanisms of increased vascular superoxide production in an experimental model of idiopathic dilated cardiomyopathy. Arterioscler Thromb Vasc Biol.

[455] Chen X., Gianferante D., Hanlin L., Fiksdal A., Breines J.G., Thoma M.V., Rohleder N. (2017). HPA-axis and inflammatory reactivity to acute stress is related with basal HPA-axis activity. Psychoneuroendocrinology.

[456] Meyer T., Wirtz P.H. (2018). Mechanisms of mitochondrial redox signaling in psychosocial stress-responsive systems: new insights into an old story. Antioxid Redox Signal.

[457] Wenzel P., Kossmann S., Munzel T., Daiber A. (2017). Redox regulation of cardiovascular inflammation - immunomodulatory function of mitochondrial and Nox-derived reactive oxygen and nitrogen species. Free Radic Biol Med.

[458] Steven S., Frenis K., Oelze M., Kalinovic S., Kuntic M., Bayo Jimenez M.T., Vujacic-Mirski K., Helmstadter J., Kroller-Schon S., Munzel T., Daiber A. (2019). Vascular inflammation and oxidative stress: major triggers for cardiovascular disease. Oxid Med Cell Longev.

[459] Eze I.C., Jeong A., Schaffner E., Rezwan F.I., Ghantous A., Foraster M., Vienneau D., Kronenberg F., Herceg Z., Vineis P., Brink M., Wunderli J.M., Schindler C., Cajochen C., Roosli M., Holloway J.W., Imboden M., Probst-Hensch N. (2020). Genome-wide DNA methylation in peripheral blood and long-term exposure to source-specific transportation noise and air pollution: the SAPALDIA study. Environ Health Perspect.

[460] Cai Y., Hansell A.L., Blangiardo M., Burton P.R., BioShaRe K. de Hoogh, Doiron D., Fortier I., Gulliver J., Hveem K., Mbatchou S., Morley D.W., Stolk R.P., Zijlema W.L., Elliott P., Hodgson S. (2017). Long-term exposure to road traffic noise, ambient air pollution, and cardiovascular risk factors in the HUNT and lifelines cohorts. Eur Heart J.

[461] Bayo Jimenez M.T., Frenis K., Kroller-Schon S., Kuntic M., Stamm P., Kvandova M., Oelze M., Li H., Steven S., Munzel T., Daiber A. (2021). Noise-induced vascular dysfunction, oxidative stress, and inflammation are improved by pharmacological modulation of the NRF2/HO-1 Axis. Antioxidants (Basel).

[462] Lin F., Zheng Y., Pan L., Zuo Z. (2020). Attenuation of noisy environment-induced neuroinflammation and dysfunction of learning and memory by minocycline during perioperative period in mice. Brain Res Bull.

[463] Kvandova M., Filippou K., Steven S., Oelze M., Kalinovic S., Stamm P., Frenis K., Vujacic-Mirski K., Sakumi K., Nakabeppu Y., Bagheri Hosseinabadi M., Dovinova I., Epe B., Munzel T., Kroller-Schon S., Daiber A. (2020). Environmental aircraft noise aggravates oxidative DNA damage, granulocyte oxidative burst and nitrate resistance in Ogg1(-/-) mice. Free Radic Res.

[464] Sun G., Lin X., Yi X., Zhang P., Liu R., Fu B., Sun Y., Li J., Jiao S., Tian T., Xu X.M., Tseng K.W., Lin C.H. (2021). Aircraft noise, like heat stress, causes cognitive impairments via similar mechanisms in male mice. Chemosphere.

[465] Wenzel P., Knorr M., Kossmann S., Stratmann J., Hausding M., Schuhmacher S., Karbach S.H., Schwenk M., Yogev N., Schulz E., Oelze M., Grabbe S., Jonuleit H., Becker C., Daiber A., Waisman A., Munzel T. (2011). Lysozyme M-positive monocytes mediate angiotensin II-induced arterial hypertension and vascular dysfunction. Circulation.

[466] Setiadi A., Korim W.S., Elsaafien K., Yao S.T. (2018). The role of the blood-brain barrier in hypertension. Exp Physiol.

[467] Liu L., Fang C., Yang J., Zhang H., Huang Y., Xuan C., Wang Y., Li S., Sha J., Zha M., Guo M. (2018). The effect of noise exposure on insulin sensitivity in mice may be mediated by the JNK/IRS1 pathway. Environ Health Prev Med.

[468] Gregory G.E., Munro K.J., Couper K.N., Pathmanaban O.N., Brough D. (2023). The NLRP3 inflammasome as a target for sensorineural hearing loss. Clin Immunol.

[469] Frye M.D., Ryan A.F., Kurabi A. (2019). Inflammation associated with noise-induced hearing loss. J Acoust Soc Am.

[470] Kim A., Sung J.H., Bang J.H., Cho S.W., Lee J., Sim C.S. (2017). Effects of self-reported sensitivity and road-traffic noise levels on the immune system. PLoS One.

[471] Kupcikova Z., Fecht D., Ramakrishnan R., Clark C., Cai Y.S. (2021). Road traffic noise and cardiovascular disease risk factors in UK Biobank. Eur Heart J.

[472] Kalsch H., Hennig F., Moebus S., Mohlenkamp S., Dragano N., Jakobs H., Memmesheimer M., Erbel R., Jockel K.H., Hoffmann B., Heinz G. (2014). Nixdorf recall study investigative. Are air pollution and traffic noise independently associated with atherosclerosis: the heinz nixdorf recall study. Eur Heart J.

[473] Hennig F., Moebus S., Reinsch N., Budde T., Erbel R., Jockel K.H., Lehmann N., Hoffmann B., Kalsch H., Heinz G. (2020). Nixdorf Recall Study Investigative. Investigation of air pollution and noise on progression of thoracic aortic calcification: results of the Heinz Nixdorf Recall Study. Eur J Prev Cardiol.

[474] Osborne M.T., Abohashem S., Naddaf N., Abbasi T., Zureigat H., Mezue K., Ghoneem A., Dar T., Cardeiro A.J., Mehta N.N., Rajagopalan S., Fayad Z.A., Tawakol A. (2023). The combined effect of air and transportation noise pollution on atherosclerotic inflammation and risk of cardiovascular disease events. J Nucl Cardiol.

[475] Kim J.M., Lee R., Kim Y., Jeong H.B., Seong Lee E., Ryoun Kim H., Park K.Y., Won Seok J. (2023). Impact of metabolic activity of vertebra and amygdala on stroke recurrence: a prospective cohort study. Circ Cardiovasc Imaging.

[476] Nagueh S.F. (2020). Left ventricular diastolic function: understanding pathophysiology, diagnosis, and prognosis with echocardiography. JACC Cardiovasc Imaging.

[477] Kang D.O., Eo J.S., Park E.J., Nam H.S., Song J.W., Park Y.H., Park S.Y., Na J.O., Choi C.U., Kim E.J., Rha S.W., Park C.G., Seo H.S., Kim C.K., Yoo H., Kim J.W. (2021). Stress-associated neurobiological activity is linked with acute plaque instability via enhanced macrophage activity: a prospective serial 18F-FDG-PET/CT imaging assessment. Eur Heart J.

[478] Dai N., Tang X., Weng X., Cai H., Zhuang J., Yang G., Zhou F., Wu P., Liu B., Duan S., Yu Y., Guo W., Ju Z., Zhang L., Wang Z., Wang Y., Lu B., Shi H., Qian J., Ge J. (2023). Stress-related neural activity associates with coronary plaque vulnerability and subsequent cardiovascular events. JACC Cardiovasc Imaging.

[479] Van Laake L.W., Luscher T.F., Young M.E. (2018). The circadian clock in cardiovascular regulation and disease: lessons from the Nobel Prize in Physiology or Medicine 2017. Eur Heart J.

[480] Furlan R., Barbic F., Piazza S., Tinelli M., Seghizzi P., Malliani A. (2000). Modifications of cardiac autonomic profile associated with a shift schedule of work. Circulation.

[481] Thosar S.S., Butler M.P., Shea S.A. (2018). Role of the circadian system in cardiovascular disease. J Clin Invest.

[482] Morris C.J., Purvis T.E., Hu K., Scheer F.A. (2016). Circadian misalignment increases cardiovascular disease risk factors in humans. Proc Natl Acad Sci U S A.

[483] Harma M., Ojajarvi A., Koskinen A., Lie J.A., Hansen J. (2023). Shift work with and without night shifts and breast cancer risk in a cohort study from Finland. Occup Environ Med.

[484] WHO (2020).

[485] Crnko S., Du Pre B.C., Sluijter J.P.G., Van Laake L.W. (2019). Circadian rhythms and the molecular clock in cardiovascular biology and disease. Nat Rev Cardiol.

[486] Jorgensen J.T., Rozing M.P., Westendorp R.G.J., Hansen J., Stayner L.T., Simonsen M.K., Andersen Z.J. (2021). Shift work and incidence of psychiatric disorders: the Danish Nurse Cohort study. J Psychiatr Res.

[487] Schmalen I., Reischl S., Wallach T., Klemz R., Grudziecki A., Prabu J.R., Benda C., Kramer A., Wolf E. (2014). Interaction of circadian clock proteins CRY1 and PER2 is modulated by zinc binding and disulfide bond formation. Cell.

[488] Putker M., O'Neill J.S. (2016). Reciprocal control of the circadian clock and cellular redox state - a critical appraisal. Mol Cells.

[489] Daiber A., Frenis K., Kuntic M., Li H., Wolf E., Kilgallen A.B., Lecour S., Van Laake L.W., Schulz R., Hahad O., Munzel T. (2022). Redox regulatory changes of circadian rhythm by the environmental risk factors traffic noise and air pollution. Antioxid Redox Signal.

[490] Park J.S., Cederroth C.R., Basinou V., Meltser I., Lundkvist G., Canlon B. (2016). Identification of a circadian clock in the inferior colliculus and its dysregulation by noise exposure. J Neurosci.

[491] Jensen K., Hahn N.E., Palme R., Saxton K., Francis D.D. (2010). Vacuum-cleaner noise and acute stress responses in female C57BL/6 mice (Mus musculus). J Am Assoc Lab Anim Sci.

[492] Versteegh C.P.C., Tserga E., Fontana J.M., Moreno-Paublete R., Sarlus H., Zisiadis G.A., Cederroth C.R., Canlon B. (2022). Differential effects of noise exposure between substrains of CBA mice. Hear Res.

[493] Fontana J.M., Tserga E., Sarlus H., Canlon B., Cederroth C. (2019). Impact of noise exposure on the circadian clock in the auditory system. J Acoust Soc Am.

[494] Li S., Zheng H., Xing Z., Liu Y., Han L., Wang Z., Yu L. (2022). The circadian timing of noise exposure influences noise-induced inflammatory responses in the mouse cochlea. Braz J Otorhinolaryngol.

[495] Merbitz-Zahradnik T., Wolf E. (2015). How is the inner circadian clock controlled by interactive clock proteins?: structural analysis of clock proteins elucidates their physiological role. FEBS Lett.

[496] Oteiza P.I. (2012). Zinc and the modulation of redox homeostasis. Free Radic Biol Med.

[497] Czarna A., Berndt A., Singh H.R., Grudziecki A., Ladurner A.G., Timinszky G., Kramer A., Wolf E. (2013). Structures of Drosophila cryptochrome and mouse cryptochrome1 provide insight into circadian function. Cell.

[498] Lamia K.A., Sachdeva U.M., DiTacchio L., Williams E.C., Alvarez J.G., Egan D.F., Vasquez D.S., Juguilon H., Panda S., Shaw R.J., Thompson C.B., Evans R.M. (2009). AMPK regulates the circadian clock by cryptochrome phosphorylation and degradation. Science.

[499] Lee Y., Kim E.K. (2013). AMP-activated protein kinase as a key molecular link between metabolism and clockwork. Exp Mol Med.

[500] Jordan S.D., Lamia K.A. (2013). AMPK at the crossroads of circadian clocks and metabolism. Mol Cell Endocrinol.

[501] Sanada K., Harada Y., Sakai M., Todo T., Fukada Y. (2004). Serine phosphorylation of mCRY1 and mCRY2 by mitogen-activated protein kinase. Genes Cells.

[502] Asher G., Reinke H., Altmeyer M., Gutierrez-Arcelus M., Hottiger M.O., Schibler U. (2010). Poly(ADP-ribose) polymerase 1 participates in the phase entrainment of circadian clocks to feeding. Cell.

[503] Reinke H., Asher G. (2019). Crosstalk between metabolism and circadian clocks. Nat Rev Mol Cell Biol.

[504] Zheng X., Yang Z., Yue Z., Alvarez J.D., Sehgal A. (2007). FOXO and insulin signaling regulate sensitivity of the circadian clock to oxidative stress. Proc Natl Acad Sci U S A.

[505] Chaves I., van der Horst G.T., Schellevis R., Nijman R.M., Koerkamp M.G., Holstege F.C., Smidt M.P., Hoekman M.F. (2014). Insulin-FOXO3 signaling modulates circadian rhythms via regulation of clock transcription. Curr Biol.

[506] Nakahata Y., Kaluzova M., Grimaldi B., Sahar S., Hirayama J., Chen D., Guarente L.P., Sassone-Corsi P. (2008). The NAD+-dependent deacetylase SIRT1 modulates CLOCK-mediated chromatin remodeling and circadian control. Cell.

[507] Asher G., Gatfield D., Stratmann M., Reinke H., Dibner C., Kreppel F., Mostoslavsky R., Alt F.W., Schibler U. (2008). SIRT1 regulates circadian clock gene expression through PER2 deacetylation. Cell.

[508] Rey G., Reddy A.B. (2015). Interplay between cellular redox oscillations and circadian clocks. Diabetes Obes Metab.

[509] Kinoshita C., Aoyama K., Nakaki T. (2018). Neuroprotection afforded by circadian regulation of intracellular glutathione levels: a key role for miRNAs. Free Radic Biol Med.

[510] Ordovas J.M., Smith C.E. (2010). Epigenetics and cardiovascular disease. Nat Rev Cardiol.

[511] Kuznetsova T., Prange K.H.M., Glass C.K., de Winther M.P.J. (2020). Transcriptional and epigenetic regulation of macrophages in atherosclerosis. Nat Rev Cardiol.

[512] Mikhed Y., Gorlach A., Knaus U.G., Daiber A. (2015). Redox regulation of genome stability by effects on gene expression, epigenetic pathways and DNA damage/repair. Redox Biol.

[513] Kietzmann T., Petry A., Shvetsova A., Gerhold J.M., Gorlach A. (2017). The epigenetic landscape related to reactive oxygen species formation in the cardiovascular system. Br J Pharmacol.

[514] Leisegang M.S., Schroder K., Brandes R.P. (2017).

[515] Greco C.M., Condorelli G. (2015). Epigenetic modifications and noncoding RNAs in cardiac hypertrophy and failure. Nat Rev Cardiol.

[516] Guo L., Li P.H., Li H., Colicino E., Colicino S., Wen Y., Zhang R., Feng X., Barrow T.M., Cayir A., Baccarelli A.A., Byun H.M. (2017). Effects of environmental noise exposure on DNA methylation in the brain and metabolic health. Environ Res.

[517] Lavinsky J., Kasperbauer G., Bento R.F., Mendonca A., Wang J., Crow A.L., Allayee H., Friedman R.A. (2021). Noise exposure and distortion product otoacoustic emission suprathreshold amplitudes: a genome-wide association study. Audiol Neurootol.

[518] Wei W., Shi X., Xiong W., He L., Du Z.D., Qu T., Qi Y., Gong S.S., Liu K., Ma X. (2020). RNA-Seq profiling and Co-expression network analysis of long noncoding RNAs and mRNAs reveal novel pathogenesis of noise-induced hidden hearing loss. Neuroscience.

[519] Miguel V., Cui J.Y., Daimiel L., Espinosa-Diez C., Fernandez-Hernando C., Kavanagh T.J., Lamas S. (2018). The role of MicroRNAs in environmental risk factors, noise-induced hearing loss, and mental stress. Antioxid Redox Signal.

[520] Miguel V., Lamas S., Espinosa-Diez C. (2020). Role of non-coding-RNAs in response to environmental stressors and consequences on human health. Redox Biol.

[521] Meerson A., Cacheaux L., Goosens K.A., Sapolsky R.M., Soreq H., Kaufer D. (2010). Changes in brain MicroRNAs contribute to cholinergic stress reactions. J Mol Neurosci.

[522] Leso V., Fontana L., Finiello F., De Cicco L., Luigia Ercolano M., Iavicoli I. (2020). Noise induced epigenetic effects: a systematic review. Noise Health.

[523] Daiber A., Lelieveld J., Steven S., Oelze M., Kroller-Schon S., Sorensen M., Munzel T. (2019). The "exposome" concept - how environmental risk factors influence cardiovascular health. Acta Biochim Pol.

[524] Munzel T., Sorensen M., Hahad O., Nieuwenhuijsen M., Daiber A. (2023). The contribution of the exposome to the burden of cardiovascular disease. Nat Rev Cardiol.

[525] Maitre L., de Bont J., Casas M., Robinson O., Aasvang G.M., Agier L., Andrusaityte S., Ballester F., Basagana X., Borras E., Brochot C., Bustamante M., Carracedo A., de Castro M., Dedele A., Donaire-Gonzalez D., Estivill X., Evandt J., Fossati S., Giorgis-Allemand L., Granum R.G.J.B., Grazuleviciene R., Bjerve Gutzkow K., Smastuen Haug L., Hernandez-Ferrer C., Heude B., Ibarluzea J., Julvez J., Karachaliou M., Keun H.C., Hjertager Krog N., Lau C.E., Leventakou V., Lyon-Caen S., Manzano C., Mason D., McEachan R., Meltzer H.M., Petraviciene I., Quentin J., Roumeliotaki T., Sabido E., Saulnier P.J., Siskos A.P., Siroux V., Sunyer J., Tamayo I., Urquiza J., Vafeiadi M., van Gent D., Vives-Usano M., Waiblinger D., Warembourg C., Chatzi L., Coen M., van den Hazel P., Nieuwenhuijsen M.J., Slama R., Thomsen C., Wright J., Vrijheid M. (2018). Human Early Life Exposome (HELIX) study: a European population-based exposome cohort. BMJ Open.

[526] de Prado-Bert P., Ruiz-Arenas C., Vives-Usano M., Andrusaityte S., Cadiou S., Carracedo A., Casas M., Chatzi L., Dadvand P., Gonzalez J.R., Grazuleviciene R., Gutzkow K.B., Haug L.S., Hernandez-Ferrer C., Keun H.C., Lepeule J., Maitre L., McEachan R., Nieuwenhuijsen M.J., Pelegri D., Robinson O., Slama R., Vafeiadi M., Sunyer J., Vrijheid M., Bustamante M. (2021). The early-life exposome and epigenetic age acceleration in children. Environ Int.

[527] Strain J., Spaans F., Serhan M., Davidge S.T., Connor K.L. (2022). Programming of weight and obesity across the lifecourse by the maternal metabolic exposome: a systematic review. Mol Aspects Med.

[528] Shankar K., Pivik R.T., Johnson S.L., van Ommen B., Demmer E., Murray R. (2018). Environmental forces that shape early development: what we know and still need to know. Curr Dev Nutr.

[529] Li S., Wang W., Zhang D., Li W., Lund J., Kruse T., Mengel-From J., Christensen K., Tan Q. (2021). Differential regulation of the DNA methylome in adults born during the Great Chinese Famine in 1959-1961. Genomics.

[530] Ramirez V., Bautista R.J., Frausto-Gonzalez O., Rodriguez-Pena N., Betancourt E.T., Bautista C.J. (2023). Developmental programming in animal models: critical evidence of current environmental negative changes. Reprod Sci.

[531] Choi S.H., Choi C.H. (2015). Noise-induced neural degeneration and therapeutic effect of antioxidant drugs. J Audiol Otol.

[532] Daiber A., Chlopicki S. (2020). Revisiting pharmacology of oxidative stress and endothelial dysfunction in cardiovascular disease: evidence for redox-based therapies. Free Radic Biol Med.

[533] Murphy M.P., Bayir H., Belousov V., Chang C.J., Davies K.J.A., Davies M.J., Dick T.P., Finkel T., Forman H.J., Janssen-Heininger Y., Gems D., Kagan V.E., Kalyanaraman B., Larsson N.G., Milne G.L., Nyström T., Poulsen H.E., Radi R., Van Remmen H., Schumacker P.T., Thornalley P.J., Toyokuni S., Winterbourn C.C., Yin H., Halliwell B. (2022). Guidelines for measuring reactive oxygen species and oxidative damage in cells and in vivo. Nat Metab.

[534] Sies H., Belousov V.V., Chandel N.S., Davies M.J., Jones D.P., Mann G.E., Murphy M.P., Yamamoto M., Winterbourn C. (2022). Defining roles of specific reactive oxygen species (ROS) in cell biology and physiology. Nat Rev Mol Cell Biol.

[535] Daiber A., Oelze M., Steven S., Kroller-Schon S., Munzel T. (2017). Taking up the cudgels for the traditional reactive oxygen and nitrogen species detection assays and their use in the cardiovascular system. Redox Biol.

[536] Frijhoff J., Winyard P.G., Zarkovic N., Davies S.S., Stocker R., Cheng D., Knight A.R., Taylor E.L., Oettrich J., Ruskovska T., Gasparovic A.C., Cuadrado A., Weber D., Poulsen H.E., Grune T., Schmidt H.H., Ghezzi P. (2015). Clinical relevance of biomarkers of oxidative stress. Antioxid Redox Signal.

[537] Ghezzi P. (2020). Environmental risk factors and their footprints in vivo - a proposal for the classification of oxidative stress biomarkers. Redox Biol.

[538] Giustarini D., Colombo G., Garavaglia M.L., Astori E., Portinaro N.M., Reggiani F., Badalamenti S., Aloisi A.M., Santucci A., Rossi R., Milzani A., Dalle-Donne I. (2017). Assessment of glutathione/glutathione disulphide ratio and S-glutathionylated proteins in human blood, solid tissues, and cultured cells. Free Radic Biol Med.

[539] Monostori P., Wittmann G., Karg E., Túri S. (2009). Determination of glutathione and glutathione disulfide in biological samples: an in-depth review. J Chromatogr B Analyt Technol Biomed Life Sci.

[540] Lykkesfeldt J. (2012). Ascorbate and dehydroascorbic acid as biomarkers of oxidative stress: validity of clinical data depends on vacutainer system used. Nutr Res.

[541] Buettner G.R., Jurkiewicz B.A. (1993). Ascorbate free radical as a marker of oxidative stress: an EPR study. Free Radic Biol Med.

[542] Ito F., Sono Y., Ito T. (2019). Measurement and clinical significance of lipid peroxidation as a biomarker of oxidative stress: oxidative stress in diabetes, atherosclerosis, and chronic inflammation. Antioxidants (Basel).

[543] Niki E. (2014). Biomarkers of lipid peroxidation in clinical material. Biochim Biophys Acta.

[544] Yin H., Xu L., Porter N.A. (2011). Free radical lipid peroxidation: mechanisms and analysis. Chem Rev.

[545] Li L., Zhong S., Shen X., Li Q., Xu W., Tao Y., Yin H. (2019). Recent development on liquid chromatography-mass spectrometry analysis of oxidized lipids. Free Radic Biol Med.

[546] Milne G.L., Musiek E.S., Morrow J.D. (2005). F2-isoprostanes as markers of oxidative stress in vivo: an overview. Biomarkers.

[547] Van't Erve T.J., Lih F.B., Jelsema C., Deterding L.J., Eling T.E., Mason R.P., Kadiiska M.B. (2016). Reinterpreting the best biomarker of oxidative stress: the 8-iso-prostaglandin F2α/prostaglandin F2α ratio shows complex origins of lipid peroxidation biomarkers in animal models. Free Radic Biol Med.

[548] Hawkins C.L., Davies M.J. (2019). Detection, identification, and quantification of oxidative protein modifications. J Biol Chem.

[549] Kehm R., Baldensperger T., Raupbach J., Höhn A. (2021). Protein oxidation - formation mechanisms, detection and relevance as biomarkers in human diseases. Redox Biol.

[550] Manta B., Gladyshev V.N. (2017). Regulated methionine oxidation by monooxygenases. Free Radic Biol Med.

[551] Larsen E.L., Weimann A., Poulsen H.E. (2019). Interventions targeted at oxidatively generated modifications of nucleic acids focused on urine and plasma markers. Free Radic Biol Med.

[552] Graille M., Wild P., Sauvain J.-J., Hemmendinger M., Guseva Canu I., Hopf N.B. (2020). Urinary 8-OHdG as a biomarker for oxidative stress: a systematic literature review and meta-analysis. International Journal of Molecular Sciences.

[553] Henriksen T., Weimann A., Larsen E.L., Poulsen H.E. (2021). Quantification of 8-oxo-7,8-dihydro-2'-deoxyguanosine and 8-oxo-7,8-dihydro-guanosine concentrations in urine and plasma for estimating 24-h urinary output. Free Radic Biol Med.

[554] Cuadrado A., Manda G., Hassan A., Alcaraz M.J., Barbas C., Daiber A., Ghezzi P., León R., López M.G., Oliva B., Pajares M., Rojo A.I., Robledinos-Antón N., Valverde A.M., Guney E., Schmidt H. (2018). Transcription factor NRF2 as a therapeutic target for chronic diseases: a systems medicine approach. Pharmacol Rev.

[555] Mondal N.K., Saha H., Mukherjee B., Tyagi N., Ray M.R. (2018). Inflammation, oxidative stress, and higher expression levels of Nrf2 and NQO1 proteins in the airways of women chronically exposed to biomass fuel smoke. Mol Cell Biochem.

[556] Siems W., Grune T. (2003). Intracellular metabolism of 4-hydroxynonenal. Mol Aspects Med.

[557] Castro J.P., Jung T., Grune T., Siems W. (2017). 4-Hydroxynonenal (HNE) modified proteins in metabolic diseases. Free Radic Biol Med.

[558] Pickering A.M., Koop A.L., Teoh C.Y., Ermak G., Grune T., Davies K.J. (2010). The immunoproteasome, the 20S proteasome and the PA28alphabeta proteasome regulator are oxidative-stress-adaptive proteolytic complexes. Biochem J.

[559] Grimm S., Ott C., Horlacher M., Weber D., Hohn A., Grune T. (2012). Advanced-glycation-end-product-induced formation of immunoproteasomes: involvement of RAGE and Jak2/STAT1. Biochem J.

[560] Savas J.N. (2023). The cochlea is built to last a lifetime. Hear Res.

[561] Jongkamonwiwat N., Ramirez M.A., Edassery S., Wong A.C.Y., Yu J., Abbott T., Pak K., Ryan A.F., Savas J.N. (2020). Noise exposures causing hearing loss generate proteotoxic stress and activate the proteostasis network. Cell Rep.

[562] Grune T., Berger M.M. (2007). Markers of oxidative stress in ICU clinical settings: present and future. Curr Opin Clin Nutr Metab Care.

[563] Lichtenberg D., Pinchuk I., Yonassi E., Weber D., Grune T. (2023). Oxidative stress is a concept, not an indication for selective antioxidant treatment. Antioxidants (Basel).

[564] Egea J., Fabregat I., Frapart Y.M., Ghezzi P., Gorlach A., Kietzmann T., Kubaichuk K., Knaus U.G., Lopez M.G., Olaso-Gonzalez G., Petry A., Schulz R., Vina J., Winyard P., Abbas K., Ademowo O.S., Afonso C.B., Andreadou I., Antelmann H., Antunes F., Aslan M., Bachschmid M.M., Barbosa R.M., Belousov V., Berndt C., Bernlohr D., Bertran E., Bindoli A., Bottari S.P., Brito P.M., Carrara G., Casas A.I., Chatzi A., Chondrogianni N., Conrad M., Cooke M.S., Costa J.G., Cuadrado A., My-Chan Dang P., De Smet B., Debelec-Butuner B., Dias I.H.K., Dunn J.D., Edson A.J., El Assar M., El-Benna J., Ferdinandy P., Fernandes A.S., Fladmark K.E., Forstermann U., Giniatullin R., Giricz Z., Gorbe A., Griffiths H., Hampl V., Hanf A., Herget J., Hernansanz-Agustin P., Hillion M., Huang J., Ilikay S., Jansen-Durr P., Jaquet V., Joles J.A., Kalyanaraman B., Kaminskyy D., Karbaschi M., Kleanthous M., Klotz L.O., Korac B., Korkmaz K.S., Koziel R., Kracun D., Krause K.H., Kren V., Krieg T., Laranjinha J., Lazou A., Li H., Martinez-Ruiz A., Matsui R., McBean G.J., Meredith S.P., Messens J., Miguel V., Mikhed Y., Milisav I., Milkovic L., Miranda-Vizuete A., Mojovic M., Monsalve M., Mouthuy P.A., Mulvey J., Munzel T., Muzykantov V., Nguyen I.T.N., Oelze M., Oliveira N.G., Palmeira C.M., Papaevgeniou N., Pavicevic A., Pedre B., Peyrot F., Phylactides M., Pircalabioru G.G., Pitt A.R., Poulsen H.E., Prieto I., Rigobello M.P., Robledinos-Anton N., Rodriguez-Manas L., Rolo A.P., Rousset F., Ruskovska T., Saraiva N., Sasson S., Schroder K., Semen K., Seredenina T., Shakirzyanova A., Smith G.L., Soldati T., Sousa B.C., Spickett C.M., Stancic A., Stasia M.J., Steinbrenner H., Stepanic V., Steven S., Tokatlidis K., Tuncay E., Turan B., Ursini F., Vacek J., Vajnerova O., Valentova K., Van Breusegem F., Varisli L., Veal E.A., Yalcin A.S., Yelisyeyeva O., Zarkovic N., Zatloukalova M., Zielonka J., Touyz R.M., Papapetropoulos A., Grune T., Lamas S., Schmidt H., Di Lisa F., Daiber A. (2017). European contribution to the study of ROS: a summary of the findings and prospects for the future from the COST action BM1203 (EU-ROS). Redox Biol.

[565] Bayo Jimenez M.T., Frenis K., Hahad O., Steven S., Cohen G., Cuadrado A., Munzel T., Daiber A. (2022). Protective actions of nuclear factor erythroid 2-related factor 2 (NRF2) and downstream pathways against environmental stressors. Free Radic Biol Med.

[566] Honkura Y., Matsuo H., Murakami S., Sakiyama M., Mizutari K., Shiotani A., Yamamoto M., Morita I., Shinomiya N., Kawase T., Katori Y., Motohashi H. (2016). NRF2 is a key target for prevention of noise-induced hearing loss by reducing oxidative damage of cochlea. Sci Rep.

[567] Kvandova M., Rajlic S., Stamm P., Schmal I., Mihalikova D., Kuntic M., Bayo Jimenez M.T., Hahad O., Kollarova M., Ubbens H., Strohm L., Frenis K., Duerr G.D., Foretz M., Viollet B., Ruan Y., Jiang S., Tang Q., Kleinert H., Rapp S., Gericke A., Schulz E., Oelze M., Keaney J.F., Daiber A., Kroller-Schon S., Jansen T., Munzel T. (2023). Mitigation of aircraft noise-induced vascular dysfunction and oxidative stress by exercise, fasting, and pharmacological alpha1AMPK activation: molecular proof of a protective key role of endothelial alpha1AMPK against environmental noise exposure. Eur J Prev Cardiol.

[568] Matzinger M., Fischhuber K., Poloske D., Mechtler K., Heiss E.H. (2020). AMPK leads to phosphorylation of the transcription factor Nrf2, tuning transactivation of selected target genes. Redox Biol.

[569] Fasipe B., Laher I. (2023). Nrf2 modulates the benefits of evening exercise in type 2 diabetes. Sports Med Health Sci.

[570] Golbidi S., Daiber A., Korac B., Li H., Essop M.F., Laher I. (2017). Health benefits of fasting and caloric restriction. Curr Diab Rep.

[571] Mahmoodzadeh Y., Mahmoudi J., Gorgani-Firuzjaee S., Mohtavinejad N., Namvaran A. (2021). Effects of N-acetylcysteine on noise exposure-induced oxidative stress and depressive- and anxiety-like behaviors in adult male mice. Basic Clin Neurosci.

[572] Sakuma N. (1984). [Changes of neurotransmitter, lipid peroxide and their metabolic related enzyme activities in the brain of rats exposed to noise and vitamin E]. Hokkaido Igaku Zasshi.

[573] Sikandaner H.E., Park S.Y., Kim M.J., Park S.N., Yang D.W. (2017). Neuroprotective effects of sildenafil against oxidative stress and memory dysfunction in mice exposed to noise stress. Behav Brain Res.

[574] Koc E.R., Ersoy A., Ilhan A., Erken H.A., Sahin S. (2015). Is rosuvastatin protective against on noise-induced oxidative stress in rat serum?. Noise Health.

[575] Molina S.J., Miceli M., Guelman L.R. (2016). Noise exposure and oxidative balance in auditory and extra-auditory structures in adult and developing animals. Pharmacological approaches aimed to minimize its effects. Pharmacol Res.

[576] Lavinsky J., Crow A.L., Pan C., Wang J., Aaron K.A., Ho M.K., Li Q., Salehide P., Myint A., Monges-Hernadez M., Eskin E., Allayee H., Lusis A.J., Friedman R.A. (2015). Genome-wide association study identifies nox3 as a critical gene for susceptibility to noise-induced hearing loss. PLoS Genet.

[577] Rousset F., Nacher-Soler G., Kokje V.B.C., Sgroi S., Coelho M., Krause K.H., Senn P. (2022). NADPH oxidase 3 deficiency protects from noise-induced sensorineural hearing loss. Front Cell Dev Biol.

[578] Clark C., Paunovic K. (2018). WHO environmental noise guidelines for the European region: a systematic review on environmental noise and quality of life, wellbeing and mental health. Int J Environ Res Public Health.

[579] Diekmann K., Bockelmann I., Karlsen H.R., Lux A., Thielmann B. (2020). Effort-reward imbalance, mental health and burnout in occupational groups that face mental stress. J Occup Environ Med.

[580] Steptoe A., Kivimaki M. (2013). Stress and cardiovascular disease: an update on current knowledge. Annu Rev Public Health.

[581] Dragano N., Siegrist J., Nyberg S.T., Lunau T., Fransson E.I., Alfredsson L., Bjorner J.B., Borritz M., Burr H., Erbel R., Fahlen G., Goldberg M., Hamer M., Heikkila K., Jockel K.H., Knutsson A., Madsen I.E.H., Nielsen M.L., Nordin M., Oksanen T., Pejtersen J.H., Pentti J., Rugulies R., Salo P., Schupp J., Singh-Manoux A., Steptoe A., Theorell T., Vahtera J., Westerholm P.J.M., Westerlund H., Virtanen M., Zins M., Batty G.D., Kivimaki M., consortium I.P.-W. (2017). Effort-reward imbalance at work and incident coronary heart disease: a multicohort study of 90,164 individuals. Epidemiology.

[582] Selander J., Bluhm G., Nilsson M., Hallqvist J., Theorell T., Willix P., Pershagen G. (2013). Joint effects of job strain and road-traffic and occupational noise on myocardial infarction. Scand J Work Environ Health.

[583] Jarczok M.N., Jarczok M., Mauss D., Koenig J., Li J., Herr R.M., Thayer J.F. (2013). Autonomic nervous system activity and workplace stressors--a systematic review. Neurosci Biobehav Rev.

[584] Bierhaus A., Wolf J., Andrassy M., Rohleder N., Humpert P.M., Petrov D., Ferstl R., von Eynatten M., Wendt T., Rudofsky G., Joswig M., Morcos M., Schwaninger M., McEwen B., Kirschbaum C., Nawroth P.P. (2003). A mechanism converting psychosocial stress into mononuclear cell activation. Proc Natl Acad Sci U S A.

[585] Takaki J. (2013). Associations of job stress indicators with oxidative biomarkers in Japanese men and women. Int J Environ Res Public Health.

[586] Aschbacher K., O'Donovan A., Wolkowitz O.M., Dhabhar F.S., Su Y., Epel E. (2013). Good stress, bad stress and oxidative stress: insights from anticipatory cortisol reactivity. Psychoneuroendocrinology.

[587] Colaianna M., Schiavone S., Zotti M., Tucci P., Morgese M.G., Backdahl L., Holmdahl R., Krause K.H., Cuomo V., Trabace L. (2013). Neuroendocrine profile in a rat model of psychosocial stress: relation to oxidative stress. Antioxid Redox Signal.

[588] Nishijima Y., Cao S., Chabowski D.S., Korishettar A., Ge A., Zheng X., Sparapani R., Gutterman D.D., Zhang D.X. (2017). Contribution of K(V)1.5 channel to hydrogen peroxide-induced human arteriolar dilation and its modulation by coronary artery disease. Circ Res.

[589] Cervantes Gracia K., Llanas-Cornejo D., Husi H. (2017). CVD and oxidative stress. J Clin Med.

[590] Steven S., Daiber A., Dopheide J.F., Munzel T., Espinola-Klein C. (2017). Peripheral artery disease, redox signaling, oxidative stress - basic and clinical aspects. Redox Biol.

[591] Munzel T., Sorensen M., Hahad O., Nieuwenhuijsen M., Daiber A. (2023). The contribution of the exposome to the burden of cardiovascular disease. Nat Rev Cardiol.

[592] Wild C.P., Scalbert A., Herceg Z. (2013). Measuring the exposome: a powerful basis for evaluating environmental exposures and cancer risk. Environ Mol Mutagen.

[593] Beulens J.W.J., Pinho M.G.M., Abreu T.C., den Braver N.R., Lam T.M., Huss A., Vlaanderen J., Sonnenschein T., Siddiqui N.Z., Yuan Z., Kerckhoffs J., Zhernakova A., Brandao Gois M.F., Vermeulen R.C.H. (2022). Environmental risk factors of type 2 diabetes-an exposome approach. Diabetologia.

[594] Vlaanderen J., de Hoogh K., Hoek G., Peters A., Probst-Hensch N., Scalbert A., Melen E., Tonne C., de Wit G.A., Chadeau-Hyam M., Katsouyanni K., Esko T., Jongsma K.R., Vermeulen R. (2021). Developing the building blocks to elucidate the impact of the urban exposome on cardiometabolic-pulmonary disease: the EU EXPANSE project. Environ Epidemiol.

[595] Ronkainen J., Nedelec R., Atehortua A., Balkhiyarova Z., Cascarano A., Ngoc Dang V., Elhakeem A., van Enckevort E., Goncalves Soares A., Haakma S., Halonen M., Heil K.F., Heiskala A., Hyde E., Jacquemin B., Keikkala E., Kerckhoffs J., Klavus A., Kopinska J.A., Lepeule J., Marazzi F., Motoc I., Naatanen M., Ribbenstedt A., Rundblad A., Savolainen O., Simonetti V., de Toro Eadie N., Tzala E., Ulrich A., Wright T., Zarei I., d'Amico E., Belotti F., Brunius C., Castleton C., Charles M.A., Gaillard R., Hanhineva K., Hoek G., Holven K.B., Jaddoe V.W.V., Kaakinen M.A., Kajantie E., Kavousi M., Lakka T., Matthews J., Piano Mortari A., Vaarasmaki M., Voortman T., Webster C., Zins M., Atella V., Bulgheroni M., Chadeau-Hyam M., Conti G., Evans J., Felix J.F., Heude B., Jarvelin M.R., Kolehmainen M., Landberg R., Lekadir K., Parusso S., Prokopenko I., de Rooij S.R., Roseboom T., Swertz M., Timpson N., Ulven S.M., Vermeulen R., Juola T., LongITools S. Sebert (2022). Dynamic longitudinal exposome trajectories in cardiovascular and metabolic noncommunicable diseases. Environ Epidemiol.

[596] Niedzwiecki M.M., Walker D.I., Vermeulen R., Chadeau-Hyam M., Jones D.P., Miller G.W. (2019). The exposome: molecules to populations. Annu Rev Pharmacol Toxicol.

[597] Hu X., Walker D.I., Liang Y., Smith M.R., Orr M.L., Juran B.D., Ma C., Uppal K., Koval M., Martin G.S., Neujahr D.C., Marsit C.J., Go Y.M., Pennell K.D., Miller G.W., Lazaridis K.N., Jones D.P. (2021). A scalable workflow to characterize the human exposome. Nat Commun.

[598] Jones D.P. (2016). Sequencing the exposome: a call to action. Toxicol Rep.

[599] Kampfrath T., Maiseyeu A., Ying Z., Shah Z., Deiuliis J.A., Xu X., Kherada N., Brook R.D., Reddy K.M., Padture N.P., Parthasarathy S., Chen L.C., Moffatt-Bruce S., Sun Q., Morawietz H., Rajagopalan S. (2011). Chronic fine particulate matter exposure induces systemic vascular dysfunction via NADPH oxidase and TLR4 pathways. Circ Res.

[600] Tyagi A., Chandrasekaran B., Navin A.K., Shukla V., Baby B.V., Ankem M.K., Damodaran C. (2023). Molecular interplay between NOX1 and autophagy in cadmium-induced prostate carcinogenesis. Free Radic Biol Med.

[601] Sainani K. (2016).

[602] Vermeulen R., Schymanski E.L., Barabasi A.L., Miller G.W. (2020). The exposome and health: where chemistry meets biology. Science.

[603] Rappaport S.M., Barupal D.K., Wishart D., Vineis P., Scalbert A. (2014). The blood exposome and its role in discovering causes of disease. Environ Health Perspect.

[604] Olbrich H.G., Roosli M., Herrmann E., Maschke C., Schadow K., Hahnel T., Rupprecht H.J., Kaltenbach M. (2023). Aircraft noise exposure and risk for recurrent cardiovascular events after acute coronary syndrome: a prospective patient cohort study. Environ Res.

[605] Ceylan N., Kaba S., Karaman K., Celiker M., Basbugan Y., Demir N. (2016). Investigation of the effect of the efficiency of noise at different intensities on the DNA of the newborns. Noise Health.

[606] Frenzilli G., Lenzi P., Scarcelli V., Fornai F., Pellegrini A., Soldani P., Paparelli A., Nigro M. (2004). Effects of loud noise exposure on DNA integrity in rat adrenal gland. Environ Health Perspect.

[607] Shi X., Han W., Yamamoto H., Omelchenko I., Nuttall A. (2007). Nitric oxide and mitochondrial status in noise-induced hearing loss. Free Radic Res.

